# NET-Related Gene as Potential Diagnostic Biomarkers for Diabetic Tubulointerstitial Injury

**DOI:** 10.1155/2024/4815488

**Published:** 2024-05-10

**Authors:** Yufeng Liang, Jiaqun Lin, Binsan Huang, Mengjie Weng, Tingting Zhen, Liyan Yang, Yongping Chen, Qiu Li, Jianxin Wan

**Affiliations:** ^1^Department of Nephrology, Blood Purification Research Center, The First Affiliated Hospital, Fujian Medical University, Fuzhou 350005, China; ^2^Department of Nephrology, The Second Hospital of Longyan, Longyan, Fujian 364000, China; ^3^Fujian Clinical Research Center for Metabolic Chronic Kidney Disease, The First Affiliated Hospital, Fujian Medical University, Fuzhou 350005, China; ^4^Department of Nephrology, National Regional Medical Center, Binhai Campus of the First Affiliated Hospital, Fujian Medical University, Fuzhou 350005, China

**Keywords:** bioinformatics, diabetic kidney disease, immune characteristics, NETosis, tubulointerstitial injury

## Abstract

**Background:** Tubulointerstitial injury plays a pivotal role in the progression of diabetic kidney disease (DKD), yet the link between neutrophil extracellular traps (NETs) and diabetic tubulointerstitial injury is still unclear.

**Methods:** We analyzed microarray data (GSE30122) from the Gene Expression Omnibus (GEO) database to identify differentially expressed genes (DEGs) associated with DKD's tubulointerstitial injury. Functional and pathway enrichment analyses were conducted to elucidate the involved biological processes (BP) and pathways. Weighted gene coexpression network analysis (WGCNA) identified modules associated with DKD. LASSO regression and random forest selected NET-related characteristic genes (NRGs) related to DKD tubulointerstitial injury.

**Results:** Eight hundred ninety-eight DEGs were identified from the GSE30122 dataset. A significant module associated with diabetic tubulointerstitial injury overlapped with 15 NRGs. The hub genes, CASP1 and LYZ, were identified as potential biomarkers. Functional enrichment linked these genes with immune cell trafficking, metabolic alterations, and inflammatory responses. NRGs negatively correlated with glomerular filtration rate (GFR) in the Neph v5 database. Immunohistochemistry (IHC) validated increased NRGs in DKD tubulointerstitial injury.

**Conclusion:** Our findings suggest that the CASP1 and LYZ genes may serve as potential diagnostic biomarkers for diabetic tubulointerstitial injury. Furthermore, NRGs involved in diabetic tubulointerstitial injury could emerge as prospective targets for the diagnosis and treatment of DKD.

## 1. Introduction

Diabetic kidney disease (DKD) has emerged as a primary cause of end-stage renal disease (ESRD) worldwide [1, 2], with rising incidence and considerable societal burden. However, despite advancements in treatment options [1, 3, 4], effective prevention and management of DKD remain limited, and reliable biomarkers to predict disease progression are lacking. Although recent research has mainly concentrated on glomerular diseases, emerging evidence indicates that tubulointerstitial injury may independently contribute to renal disease progression [5, 6]. More importantly, tubular damage mediated by inflammation seems to predominate over glomerular injury in early DKD [7]. Inflammation has been associated with tubulointerstitial injury through various pathways, thereby contributing to the progression of DKD [8, 9]. Neutrophil extracellular traps (NETs) are net-like DNA structures studded with histones and antimicrobial proteins that are released from activated neutrophils via NETosis, a unique cell death process. However, in addition to their antimicrobial roles, NETs have been implicated in numerous inflammatory diseases, particularly by driving autoimmunity [10]. Recent studies have highlighted increased NET deposition within the glomeruli of DKD patients, pointing to possible pathogenic roles beyond their traditional antimicrobial functions in DKD [11–13]. Immunohistochemistry (IHC) revealed increased NET deposition within glomeruli of DKD patients relative to nondiabetic controls [13, 14]. Supporting the histological findings, biochemical assays have shown increased levels of NET components, including myeloperoxidase (MPO)-DNA or neutrophil elastase (NE)-DNA complexes, and have been detected in blood and urine samples from DKD patients [13]. Notably, heightened NET levels exhibited positive correlations with clinical parameters of DKD severity, including declining glomerular filtration rate (GFR) and glomerulosclerosis [15]. These findings highlight the potential role of NETs in mediating progressive tubulointerstitial damage in DKD. In vitro studies have shown that NETs can exacerbate DKD via oxidative stress, apoptosis, and abnormal proliferation in renal tubular epithelial cells. The expression of NETs in tubular epithelium also differs at various stages of DKD, with gradual upregulation as DKD advances, likely due to increased NET formation and/or impaired NET degradation. NET expression levels within these cells also fluctuate across different DKD stages [15]. These results indicate that NETs participate in mediating tubular epithelial cell injury in DKD. Nevertheless, the exact mechanisms underlying NET-mediated tubular injury remain to be elucidated. Considering the dynamic changes of NETs during DKD progression, elucidating the key genes governing NET-mediated tubular injury may offer new perspectives into the intricate pathogenesis underlying DKD.

Bioinformatics approaches have been increasingly utilized to identify differentially expressed genes (DEGs) from gene microarrays and high-throughput sequencing data. Recent efforts [6, 16] have identified DEGs associated with tubulointerstitial injury in DKD, potentially obtaining a novel, effective approach to elucidate the pathogenic mechanisms of DKD and uncover new therapeutic targets. This study is aimed at comprehensively analyzing the association between NET-related genes (NRGs) [17] and the DEGs to discern DKD patients effectively. DEGs in DKD patients will be sourced from databases and previous literature reports. We screened differentially expressed NRGs (DE-NRGs) in DKD using various bioinformatics methods. These feature genes demonstrate notable diagnostic potential. Their expression patterns and GFR correlations will be analyzed using the Nephroseq v5 database. We also performed pathway enrichment analysis and immune cell infiltration analysis. Finally, key findings will be experimentally validated in a mouse DKD model, to provide novel insights into DKD diagnosis and treatment.

## 2. Method

### 2.1. Data Download

Access Gene Expression Omnibus (GEO) database: gene expression data were obtained from the National Center for Biotechnology Information's GEO public repository. Search query: use the search bar at the top of the page to search for diabetic nephropathy (DN) and DKD. Download dataset: locate the dataset GSE30122 [18] (GPL571, Affymetrix Human Genome U133A 2.0 Array) in the search results. This dataset consists of 10 DKD interstitial tubular samples and 24 control renal interstitial tubular samples. DE-NRGs: based on previous research, identify the gene set associated with NETs within the downloaded data [17](Frontiers|Identification of renal ischemia reperfusion injury subtypes and predictive strategies for delayed graft function and graft survival based on NRGs (http://frontiersin.org)).

### 2.2. Data Processing and Identification of Differential Expression

The raw data were transformed using log2 transformation and *Z*-score normalization. Based on the GEO database, we identified 898 DEGs associated with DKD tubular interstitial injury. The DEGs between renal tubular interstitial tissues of DKD patients and healthy controls were calculated using the R software limma package with adjusted *p* values of 0.05 and log FC (fold change) greater than 1. Subsequently, a volcano plot was generated to visualize the DEGs, and a heat map was employed to display the top 50 upregulated and top 50 downregulated DEGs. Both the volcano plot and heat map were drawn utilizing the ggplot2 package in R software.

### 2.3. Functional and Pathway Enrichment Analyses

The cluster Profiler package in R enables functional enrichment analysis of DEGs in the context of Gene Ontology (GO) and the Kyoto Encyclopedia of Genes and Genomes (KEGG) [19]. GO analysis [20] probes into the biological processes (BP) of DEGs, encompassing the categories of BP, cellular components (CC), and molecular functions (MF). Concurrently, KEGG analysis is employed to explore potential signaling pathways that are pertinent to these DEGs.

### 2.4. Weighted Gene Coexpression Network Analysis (WGCNA)

The WGCNA method is deployed to structure the GSE310122 cohort into distinct modules, utilizing the pick soft threshold function from the WGCNA software package [21]. The topological overlap matrix (TOM) and the dynamic tree cutting algorithm are applied to discern clusters (referred to as modules) of highly correlated genes. Following this, module membership (MM) values are utilized to pinpoint the final module that is significantly associated with DKD. Genes contained within this specific module are then selected for subsequent in-depth scholarly examination.

### 2.5. Screening and Validation of Signature Genes by Machine Learning Methods

To screen and validate signature genes, we employed two machine learning algorithms—the least absolute shrinkage and selection operator (LASSO) and the random forest method—to minimize the risk of prediction bias. We conducted LASSO regression using the glmnet package in R, implementing 10-fold cross-validation [22]. Simultaneously, we utilized the support vector machine-recursive feature elimination (SVM-RFE) to pinpoint optimal predictive features [23]. Additionally, we executed the random forest algorithm via the random forest package in R, determining the most suitable number of features based on the average error rate amongst candidate genes. Following modeling, genes with importance values exceeding 25 were selected. The intersecting genes identified by both the LASSO and random forest algorithms were considered core drivers of DKD tubulointerstitial injury and NET formation. The diagnostic efficacy of these genes was evaluated using the area under the receiver operating characteristic curve (AUC), with AUC values above 0.7 indicating strong diagnostic performance.

### 2.6. Gene Set Enrichment Analysis (GSEA) of Biological Functions and Pathways

To investigate the connection between hub genes and signaling pathways, we segmented the samples into high-expression and low-expression groups according to the mean expression values of hub genes. Subsequently, we conducted GSEA [24] between these two subgroups to pinpoint pathways enriched with statistical disparities (using an adjusted *p* value <0.05 as the threshold). This analysis is aimed at shedding light on the potential pathological mechanisms that hub genes may play in the progression of DKD.

### 2.7. Immune Cell Infiltration Analysis

To investigate differences in immune cell populations between patients with NET-associated DKD tubulointerstitial injury and healthy samples, we employed the CIBERSORT computational method to analyze the GSE30122 dataset [25]. The algorithm was run for 500 iterations to estimate the proportions of 22 distinct immune cell types. Following this, samples were carefully selected based on a statistical significance threshold of *p* < 0.05. This facilitated the calculation of the percentage representation of each immune cell type within the chosen samples. To further analyze the disparities in immune infiltration levels between the two cohorts, we utilized the vioplot package in R software.

### 2.8. Validation of Key Genes in Online Database

The mRNA expression profiles of NET genes in renal tubules of DKD patients were analyzed using the Nephroseq v5 database (http://www.nephroseq.org). Pearson's correlation analyses were conducted to examine associations between the screened key genes and patient GFR using this database. At last, we conducted immunology in a DKD mouse. Formalin-fixed, paraffin-embedded kidney sections were deparaffinized, rehydrated through graded ethanol, and subjected to microwave-mediated antigen retrieval in double-distilled water. Endogenous peroxidase activity was quenched with 3% hydrogen peroxide prior to blocking with 3% bovine serum albumin. Sections were incubated overnight at 4°C with primary antibodies against CASP1 (1 : 1000 dilution; Servicebio, Wuhan, China) and lysozyme (LYZ) (1 : 1000; Servicebio). An HRP-conjugated secondary antibody was then applied for 50 minutes at room temperature. Immunoperoxidase staining was developed using 3,3′-diaminobenzidine as the chromogen, followed by hematoxylin counterstaining of nuclei. Stained sections were visualized and photographed under a light microscope. Quantitative immunohistochemical analysis was performed by measuring optical density in ImageJ software (National Institutes of Health, Bethesda, MD, USA).

### 2.9. Validation of Feature Genes in DKD Mouse Model

To validate the identified hub genes in vivo, DKD was induced in 6 male DKD mice and 6 age-matched nondiabetic controls.

#### 2.9.1. Animal Preparation

Seven-week-old male C57BL/6J mice were purchased from Gempharmatech Co., Ltd (Jiangsu, China). At 8 weeks of age, the mice were intraperitoneally injected with multiple low doses of streptozotocin(STZ, Sigma-Aldrich, 50 mg/kg) for five consecutive days to induce diabetes. Control mice received citrate buffer only. Body weight and fasting blood glucose levels were monitored biweekly. Fasting blood glucose ≥ 15 mmol/L was considered indicative of successful model establishment.

#### 2.9.2. Immunohistochemical Staining

After kidney harvest and fixation in 4% paraformaldehyde, paraffin-embedded sections (3 *μ*m) underwent antigen retrieval and incubation with primary antibodies for CASP1 (1 : 1000; Servicebio) and LYZ (1 : 1000; Servicebio) overnight at 4°C. Sections were then incubated with secondary antibodies for 1 h at room temperature, followed by 3,3′-diaminobenzidine to visualize immunohistochemical staining. Stained sections were imaged under a light microscope (Olympus) and quantitatively analyzed using ImageJ software (NIH). Animal procedures were conducted in accordance with institutional guidelines and were approved by the Institutional Animal Care and Use Committee at Fujian Medical University (FJMU IACUC 2020-0104).

### 2.10. Statistical Analysis

Differences between the two groups were analyzed using unpaired Student's *t*-tests and Wilcoxon's rank-sum tests. Associations between variables were evaluated by the Pearson or Spearman correlation analyses. Statistical analyses were conducted using R software (version 4.3.1). Differences were considered statistically significant at *p* < 0.05 unless otherwise stated.

## 3. Result

### 3.1. Identification of DEGs Between Diabetic Tubulointerstitial Injury and Control Group

**Figure 1:**
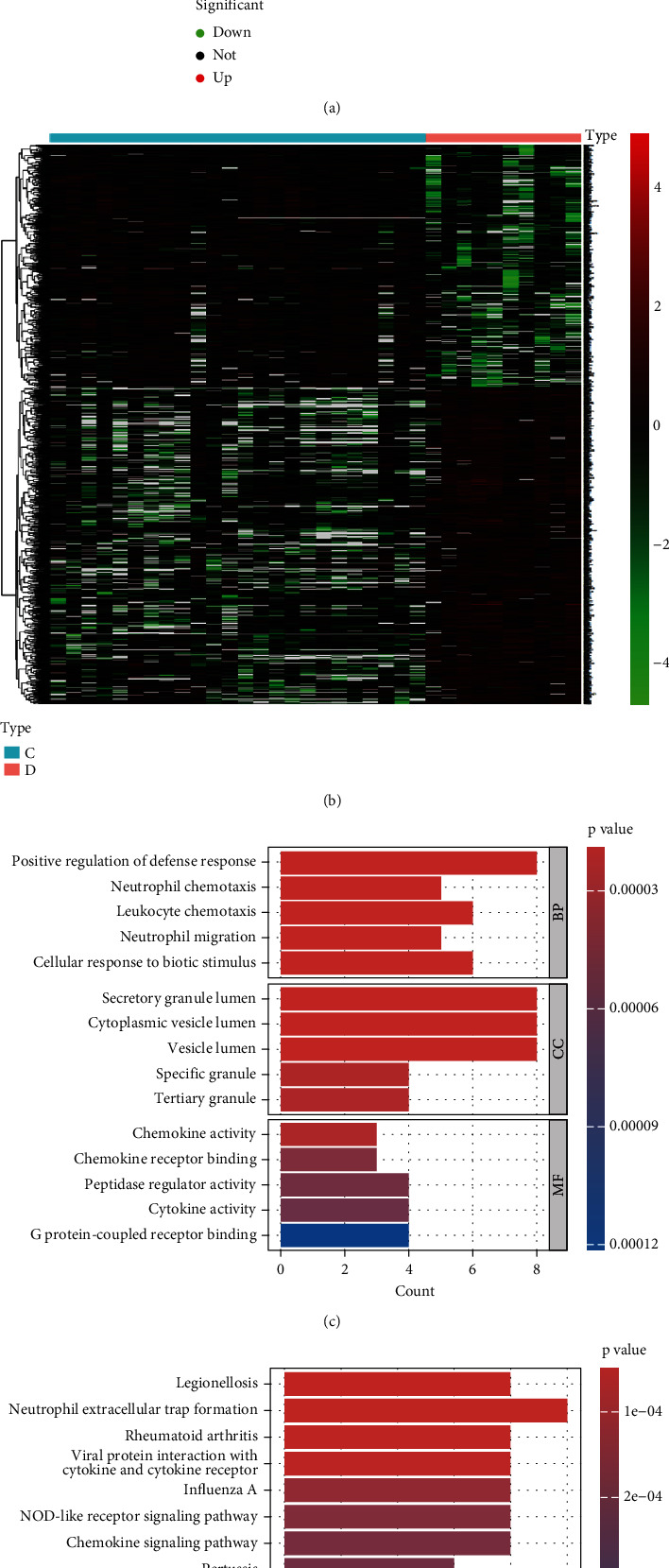
Differential expression analysis in DKD tubulointerstitial injury. (a) Volcano plot depicting the DEG profiles between diabetic tubulointerstitial injury samples and healthy controls. (b) Heat map representation of the 50 most upregulated and 50 most downregulated DEGs. Enrichment analysis of functional annotations for DEGs. (c) Top 5 enriched functional terms in biological processes (BP), cellular components (CC), and molecular functions (MF). (d) Pathway enrichment analysis using KEGG for DEGs.

DEGs from diabetic tubulointerstitial injury samples and controls were analyzed rigorously using the “limma” package in the R software for statistical analysis. The R language code for DEGs is provided in Supporting Information S1. Transcriptomic analysis identified 898 DEGs between DKD tubulointerstitial injury samples and controls, including 379 upregulated and 519 downregulated genes (Figure 1(a)). The DEGs are listed in Table S2. A heat map shows the top 50 up- and downregulated DEGs distinguishing tubulointerstitial injury samples from controls (Figure 1(b)).

### 3.2. Functional and Pathway Enrichment Analyses

Our GO analysis assessed three categories: BP, CC, and MF (Figure 1(c); Table S3). The BP category revealed significant enrichment in processes including positive regulation of defense response, neutrophil chemotaxis, leukocyte chemotaxis, neutrophil migration, and cellular response to biotic stimulus.CC enrichment analysis demonstrated that DEGs were significantly enriched in multiple secretory vesicle components, including secretory granule lumen, cytoplasmic vesicle lumen, vesicle lumen, specific granules, and tertiary granules. In the MF category, chemokine activity, chemokine receptor binding, peptidase regulator activity, cytokine activity, and G protein-coupled receptor binding were identified as playing essential roles. The KEGG pathway analysis highlighted NET formation as the most significant pathway, with *Legionellosis*, rheumatoid arthritis, and viral protein interaction as other major enriched pathways (Figure 1(d); Table S4).

### 3.3. Weighted Gene Coexpression Network Construction

**Figure 2:**
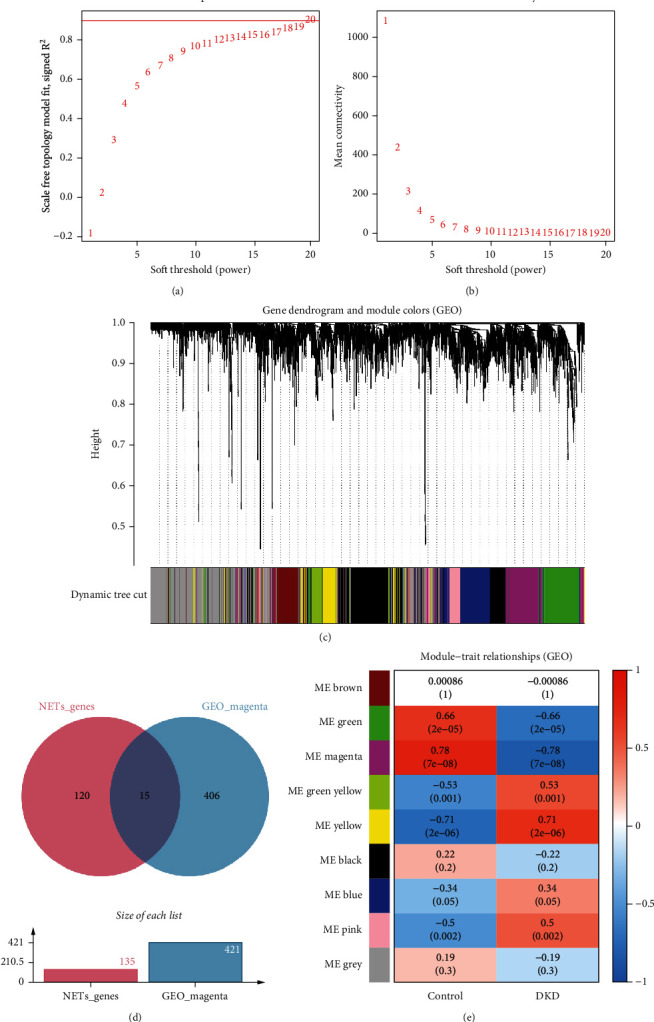
Network construction and module detection in GSE30122 using WGCNA. (a) Selection of the soft-thresholding power in WGCNA. (b) Analysis of network connectivity within WGCNA. (c) The WGCNA generated a cluster dendrogram. Identification of candidate hub genes. (d) The Venn diagram illustrating the overlap between the magenta module and NRGs. (e) Cluster dendrogram of gene modules from WGCNA.

The scale-free topology index reached its peak at a soft-thresholding power of 10 (Figure 2(a)). The mean connectivity plateaued at approximately one (Figure 2(b)), facilitating the comparison of networks. A dendrogram was formed based on topological overlap through hierarchical clustering (Figure 2(c)). Colored modules were assigned through dynamic tree cutting. The heat map indicates that the magenta module showed the highest positive correlation with DKD tubulointerstitial injury (Figure 2(e)). The Venn diagram displays 15 overlapping genes between the magenta module and NRGs (Figure 2(D)).

### 3.4. LASSO and Random Forest Approaches Were Employed to Select Optimal Feature Genes

**Figure 3:**
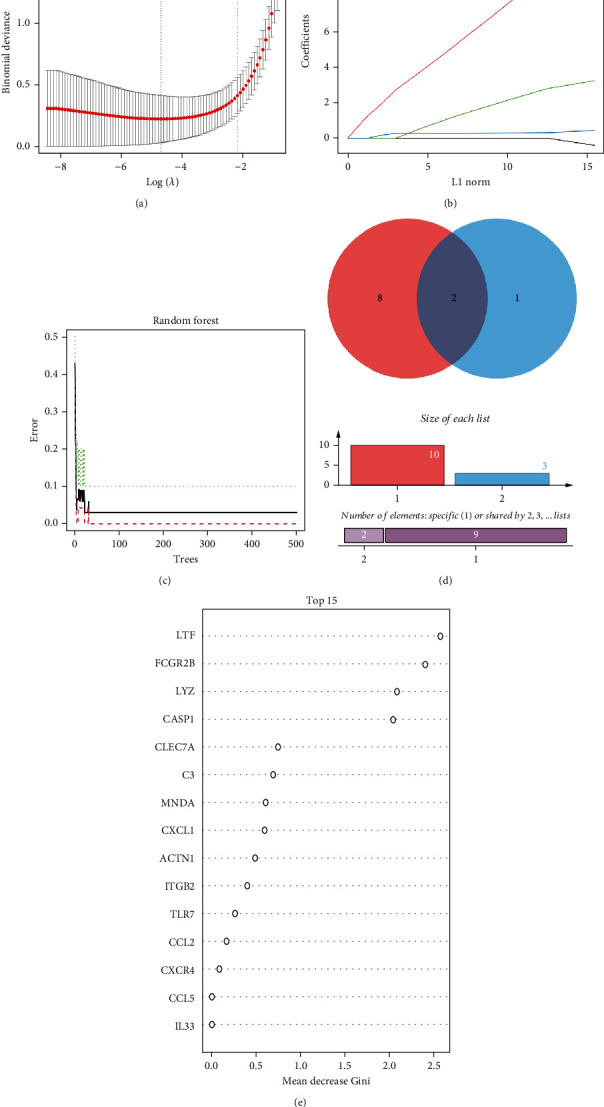
Machine learning algorithms applied to signature gene identification. (a) Penalty parameter selection in the LASSO model depicted with error bars representing standard errors. (b) LASSO coefficient profiles demonstrating shrinkage as the penalty parameter *k* increases. (c) Confidence intervals for error rates in the random forest model. (d) Interplay between LASSO and random forest models. (e) Genes with a relative importance exceeding 0.15 in the random forest model.

Feature genes were selected via LASSO regression and random forest models. LASSO regression enabled automated feature selection and optimization of the regularization parameter by cross-validation to minimize error, identifying 10 feature genes (Figures 3(a) and 3(b)). Increasing the number of decision trees led to a gradual decline in the prediction error of the random forest model, which stabilized at approximately 300 trees (Figure 3(c)). The top 15 features from the random forest model ranked LTF, FCGR2B, LYZ, and CASP1 as the most important variables (Figure 3(f)). A Venn diagram shows CASP1 and LYZ as the two feature genes commonly selected by LASSO regression and random forest models (Figure 3(d)). Feature genes identified through machine learning are shown in Table S5.

### 3.5. The Diagnostic Efficacy Evaluation and Validation of Feature Genes

**Figure 4:**
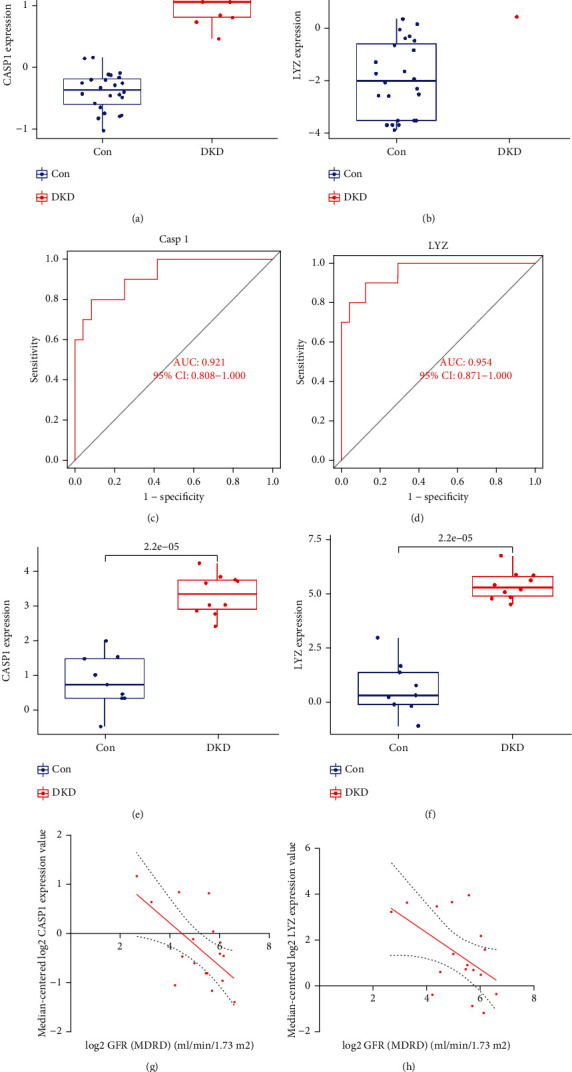
Evaluating the performance of signature genes within GSE30122. (a, b) Boxplots illustrating expression levels of signature genes in DKD tubulointerstitial injury and healthy cohort. (c, d) ROC curve analysis of the diagnostic performance of signature genes CASP1 and LYZ. (e, f) Expression levels of CASP1 and LYZ in tubulointerstitial injury patients versus healthy controls based on the Nephroseq v5 dataset. (g, h) Correlations between CASP1/LYZ expression and GFR in tubulointerstitial injury patients compared to healthy controls using the Nephroseq v5 dataset.

Boxplots showed upregulated CASP1 and LYZ mRNA expression (*p* < 0.01) in patient samples (Figures 4(a) and 4(b)). Receiver operating characteristic analysis demonstrated high area under the curve (AUC) values of 0.921 and 0.954 for CASP1 and LYZ, respectively (Figures 4(c) and 4(d)), indicating excellent diagnostic performance. To validate the expression changes, the tubulointerstitial transcriptome of DKD patients was analyzed using Nephroseq v5, confirming elevated CASP1 and LYZ mRNA levels compared to controls (Figures 4(e) and 4(f)). Correlation analysis showed significant negative correlations between estimated GFR and CASP1/LYZ expression (Figures 4(g) and 4(h)), indicating their potential as predictive biomarkers for declining renal function.

### 3.6. Function Enrichment Analysis

**Figure 5:**
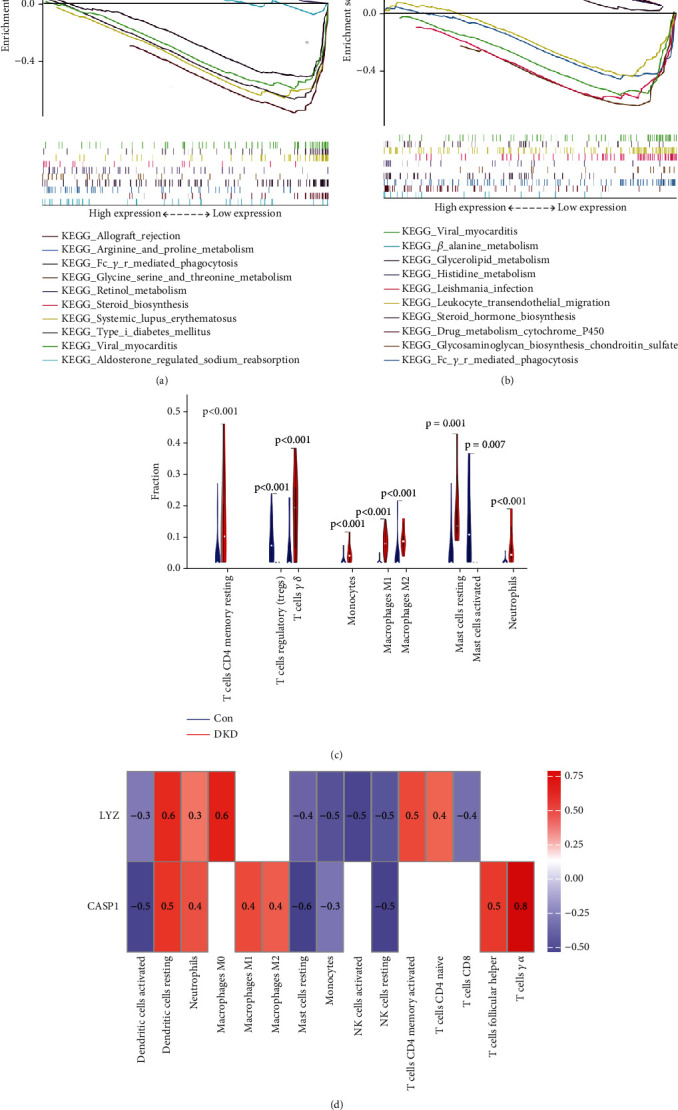
Gene set enrichment analysis (GSEA) of signature genes in DKD tubulointerstitial injury. GSEA results for (a) CASP1 and (b) LYZ in DKD versus healthy controls. Immune infiltration analysis of signature genes. (c) Comparison of immune cell subsets between DKD tubulointerstitial injury and healthy cohorts. (d) Correlations between signature genes and significantly altered immune cell types.

To elucidate the functional roles of the identified feature genes during tubulointerstitial injury, GSEA compared pathway regulation between high and low hub gene expression groups in the GSE30122 dataset. GSEA showed CASP1 and LYZ expression to be associated with phagocytosis, leukocyte migration, amino acid metabolism, and steroid biosynthesis pathways. Figures 5(a) and 5(b) display the top 10 ranked pathways by enrichment score. These functional enrichments implicate the potential involvement of CASP1 and LYZ in regulating immune cell trafficking, metabolic alterations, and inflammatory responses during DKD tubulointerstitial injury.

### 3.7. Immune Cell Infiltration Analysis

Immune cell infiltration analysis was performed to assess immunologic features. Compared to normal controls, DKD tubulointerstitial injury patients showed increased levels of CD4 memory resting T cells, regulatory T cells (Tregs), gamma delta T cells, monocytes, M1/M2 macrophages, resting/activated mast cells, and neutrophils (Figure 5(c)). Furthermore, CASP1 and LYZ expression exhibited positive correlations with resting dendritic cells, M0/M1/M2 macrophages, and neutrophils, while showing negative correlations with activated dendritic cells, resting mast cells, NK cells, and monocytes (Figure 5(d)).

### 3.8. IHC Staining

**Figure 6:**
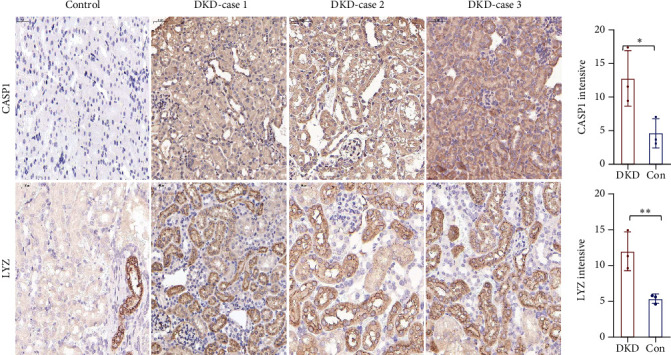
Immunohistochemistry of NRGs in mouse kidney tissues. Representative images of CASP1 and LYZ immunohistochemical staining in renal sections from diabetic kidney disease (DKD) and nondiabetic control mice (200x magnification, scale bar 50 *μ*m). ^∗^*p* < 0.05 and ^∗∗^*p* < 0.01 compared to nondiabetic controls.

We attempted to perform IHC staining for NRGs (CASP1 and LYZ) in the DKD mouse model and nondiabetic controls. The results of IHC staining revealed that CASP1 and LYZ exhibited statistical differences in the DKD group compared with the non-DKD group mainly in the tubular epithelial cells (*p* < 0.05). (Figures 6(a) and 6(b)).

## 4. Discussion

DKD is a complex, heterogeneous disorder characterized by overlapping pathogenetic mechanisms that can contribute to declining renal function [3]. Identifying and exploring potential DKD biomarkers to slow or prevent disease progression is clinically significant. Recent studies [8, 9] indicate that immune-inflammatory injury plays a crucial role in DKD pathogenesis. NETs are an inflammatory neutrophil cell death pathway characterized by the release of nonspecific components [26]. Excessive NET activation and release can elicit uncontrolled inflammation and subsequent tissue damage, owing to the accumulation of extracellular chromatin and inflammatory factors such as tumor necrosis factor-*α* (TNF-*α*) and interleukin-1*β* (IL-1*β*) [11, 13, 27]. Emerging evidence shows that DKD can promote NETosis to release self-antigens, thereby eliciting immune-inflammatory responses that contribute to disease progression [3, 11, 13, 28, 29]. NETs have been demonstrated to play a vital role in the pathogenesis and deleterious effects associated with DKD. However, the potential mechanisms of NETs in mediating tubulointerstitial injury in diabetes remain unclear. The widespread application of microarrays and bioinformatics facilitates the identification of key genes involved in diabetic tubulointerstitial injury. This may yield additional therapeutic targets for DKD.

We evaluated DEGs between DKD patients and healthy controls and explored key modules using WGCNA. NET-related signature genes for DKD tubulointerstitial injury, including CASP1 and LYZ, were identified by LASSO and random forest analysis. Validation was performed in the Neph v5 database and DKD mouse models. GSEA then explored signaling pathways associated with the signature genes. The CIBERSORT algorithm finally analyzed correlations between immune cell infiltration and signature genes in DKD tubulointerstitial injury versus healthy groups.

Caspase-1 (CASP1) is an inflammatory cysteine protease that is pivotal for maturation of the proinflammatory cytokines IL-1*β* and IL-18, which can activate neutrophils and promote NETosis [11, 30]. CASP1 critically regulates inflammation and pyroptosis. Previous studies implicate CASP1 in DKD pathogenesis. Increased renal CASP1 expression and activity are demonstrated in both animal models and human studies of diabetes [31]. These findings suggest that CASP1 plays a role in DKD not limited to a single cell type but may also mediate tubular injury [13]. These findings suggest that the role of CASP1 in DKD is not limited to a single intrinsic cell type but likely also mediates tubular injury. Although direct evidence linking CASP1 to DKD tubulointerstitial injury is currently absent, our integrative bioinformatics analysis identified an association between tubulointerstitial injury and increased CASP1 expression, given the inflammatory and cytotoxic roles of CASP1. Renal tubular CASP1 may thus have utility as an early diagnostic biomarker of tubulointerstitial injury in DKD.

LYZ is an immunomodulatory and antibacterial protease [32]. Prior bioinformatics studies show upregulated renal LYZ expression and activity in DKD patients and models [33]. LYZ has been identified as a key gene in bioinformatics analyses, with increased urinary LYZ mRNA levels correlating with renal pathology severity in DKD [32]. Moreover, increased renal LYZ is confirmed in DKD animal models by IHC [34]. Although direct evidence linking LYZ to diabetic tubulointerstitial injury is currently absent, these findings suggest that LYZ-mediated inflammation may promote interstitial injury in DKD, with utility as a biomarker. Further studies should investigate the mechanisms of LYZ in DKD and validate its diagnostic and monitoring value using in vitro and in vivo models.

Functional enrichment and immune infiltration analyses indicate aberrant immunopathology in DKD interstitial injury. GO analysis implicates neutrophil infiltration and chemotaxis in driving inflammation, while KEGG analysis emphasizes the critical role of NETs, suggesting neutrophil-mediated tissue damage via NET release [29, 35]. Further immune cell analysis reveals intricate crosstalk between innate and adaptive immunity underlying disease progression.

Activated neutrophils release NETs, stimulating macrophage recruitment and antigen presentation to trigger cytotoxic T cell responses [36]. Conversely, IFN-*γ* and TNF-*α* from T cells can reciprocally promote M1 macrophage polarization and neutrophil activation [37], perpetuating a self-amplifying inflammatory loop. CASP1/LYZ correlations show that NETs have immunomodulatory effects on macrophages, neutrophils, and dendritic and mast cells. Macrophage accumulation in DKD kidney correlates with declining renal function [38]. NETs may aggravate inflammation and fibrosis by perturbing macrophage polarization [29, 38]. In summary, infiltrating immune cells contribute to DKD, and targeting CASP1/LYZ may mitigate tubulointerstitial injury by modulating aberrant immunity. Activated macrophages can also secrete TNF-*α* and CCL2 to recruit more neutrophils [8], perpetuating a positive feedback loop exacerbating disease. Moreover, macrophage engulfment of mast cell granules may suppress excess activation [38], consistent with our findings that targeting CASP1/LYZ could ameliorate aberrant immunity and arrest DKD tubulointerstitial injury.

To investigate NRG correlations with renal function, we analyzed NRG expression in Nephroseq, finding negative correlations with GFR in DKD. Using a DKD mouse model, we performed IHC to validate the role of NRGs in tubulointerstitial injury, highlighting their biomarker potential. Our findings reveal the potential of NRGs as noninvasive biomarkers for early tubular injury in DKD, stratifying patients based on tubular damage profile. This enables more personalized treatment by targeting tubulointerstitial inflammation. Longitudinal NRG analysis may complement GFR for monitoring tubular injury progression. Effective biomarkers might lessen the need for invasive kidney biopsies. NRGs have potential as noninvasive pharmacodynamic markers of therapeutic response. Further studies are warranted to validate NRGs' diagnostic accuracy and determine their prognostic and therapeutic monitoring value.

However, limitations exist. Our literature-based NRG selection may have missed newly discovered NET genes, as NET research continues to identify novel-related genes. Additionally, a lack of human validation and potential dataset biases represent limitations. Larger DKD cohorts would help verify these findings and overcome computational biases.

## 5. Conclusion

Our study reveals CASP1 and LYZ as promising diagnostic and therapeutic targets for tubulointerstitial injury in DKD. The aberrant expression of NRGs is closely associated with the pathogenesis of tubulointerstitial lesions in DKD. Future investigations should focus on delineating the molecular mechanisms of NRGs in DKD progression. We envision that these efforts will uncover more targeted therapies to combat DKD.

## Data Availability

The data that support the findings of this study are available from the corresponding author upon reasonable request.

## Supporting Information

Supporting Information Additional supporting information can be found online in the Supporting Information section. Supporting Information S1: R language for DEGs. Table S2: DEGs identified in the gene expression microarray study. Table S3: GO enrichment analysis of DEG. Table S4: KEGG enrichment analysis of DEG. Table S5: DEG related to NETs identified through machine learning.

## References

[1] Forst T., Mathieu C., Giorgino F. (2022). New strategies to improve clinical outcomes for diabetic kidney disease. *BMC Medicine*.

[2] Hanouneh M., Echouffo Tcheugui J. B., Jaar B. G. (2021). Recent advances in diabetic kidney disease. *BMC Medicine*.

[3] Cervantes C. E., Hanouneh M., Jaar B. G. (2022). From screening to treatment: the new landscape of diabetic kidney disease. *BMC Medicine*.

[4] Fu H., Liu S., Bastacky S. I., Wang X., Tian X.-J., Zhou D. (2019). Diabetic kidney diseases revisited: a new perspective for a new era. *Molecular Metabolism*.

[5] Lin J., Cheng A., Cheng K. (2020). New insights into the mechanisms of pyroptosis and implications for diabetic kidney disease. *International Journal of Molecular Sciences*.

[6] Liu J., Duan G., Yang W. (2023). Identification of transcription factors related to diabetic tubulointerstitial injury. *Journal of Translational Medicine*.

[7] Jung S. W., Moon J.-Y. (2021). The role of inflammation in diabetic kidney disease. *The Korean Journal of Internal Medicine*.

[8] Pérez-Morales R. E., Del Pino M. D., Valdivielso J. M., Ortiz A., Mora-Fernández C., Navarro-González J. F. (2019). Inflammation in diabetic kidney disease. *Nephron*.

[9] Alicic R. Z., Johnson E. J., Tuttle K. R. (2018). Inflammatory mechanisms as new biomarkers and therapeutic targets for diabetic kidney disease. *Advances in Chronic Kidney Disease*.

[10] Pan J. T., Yi B. (2023). Research progress of neutrophil extracellular traps in kidney diseases. *Zhonghua Nei Ke Za Zhi*.

[11] Gupta A., Singh K., Fatima S. (2022). Neutrophil extracellular traps promote NLRP3 inflammasome activation and glomerular endothelial dysfunction in diabetic kidney disease. *Nutrients*.

[12] Berezin A. (2019). Neutrophil extracellular traps: the core player in vascular complications of diabetes mellitus. *Diabetes and Metabolic Syndrome: Clinical Research and Reviews*.

[13] Zheng F., Ma L., Li X. (2022). Neutrophil extracellular traps induce glomerular endothelial cell dysfunction and pyroptosis in diabetic kidney disease. *Diabetes*.

[14] Thomas H. Y., Ford Versypt A. N. (2022). Pathophysiology of mesangial expansion in diabetic nephropathy: mesangial structure, glomerular biomechanics, and biochemical signaling and regulation. *Journal of Biological Engineering*.

[15] Castanheira F. V. S., Kubes P. (2019). Neutrophils and NETs in modulating acute and chronic inflammation. *Blood*.

[16] Zhong M., Zhu E., Li N. (2023). Identification of diagnostic markers related to oxidative stress and inflammatory response in diabetic kidney disease by machine learning algorithms: evidence from human transcriptomic data and mouse experiments. *Frontiers in Endocrinology*.

[17] Wu J., Zhang F., Zheng X. (2022). Identification of renal ischemia reperfusion injury subtypes and predictive strategies for delayed graft function and graft survival based on neutrophil extracellular trap-related genes. *Frontiers in Immunology*.

[18] Woroniecka K. I., Park A. S. D., Mohtat D., Thomas D. B., Pullman J. M., Susztak K. (2011). Transcriptome analysis of human diabetic kidney disease. *Diabetes*.

[19] Yu G., Wang L.-G., Han Y., He Q.-Y. (2012). clusterProfiler: an R package for comparing biological themes among gene clusters. *OMICS: A Journal of Integrative Biology.*.

[20] Ashburner M., Ball C. A., Blake J. A. (2000). Gene ontology: tool for the unification of biology. *Nature Genetics*.

[21] Langfelder P., Horvath S. (2008). WGCNA: an R package for weighted correlation network analysis. *BMC Bioinformatics*.

[22] Tibshirani R. (1997). The lasso method for variable selection in the Cox model. *Statistics in Medicine*.

[23] Izmirlian G. (2004). Application of the random forest classification algorithm to a SELDI-TOF proteomics study in the setting of a cancer prevention trial. *Annals of the New York Academy of Sciences*.

[24] Subramanian A., Tamayo P., Mootha V. K. (2005). Gene set enrichment analysis: a knowledge-based approach for interpreting genome-wide expression profiles. *Proceedings of the National Academy of Sciences*.

[25] Newman A. M., Liu C. L., Green M. R. (2015). Robust enumeration of cell subsets from tissue expression profiles. *Nature Methods*.

[26] Lee K. H., Kronbichler A., Park D. D.-Y. (2022). Neutrophils in chronic inflammatory diseases. *Cellular & Molecular Immunology*.

[27] Cui Y., Yang Y., Tao W. (2023). Neutrophil extracellular traps induce alveolar macrophage pyroptosis by regulating NLRP3 deubiquitination, aggravating the development of septic lung injury. *Journal of Inflammation Research*.

[28] Wu T., Ding L., Andoh V., Zhang J., Chen L. (2023). The mechanism of hyperglycemia-induced renal cell injury in diabetic nephropathy disease: an update. *Life (Basel).*.

[29] Josefs T., Barrett T. J., Brown E. J. (2020). Neutrophil extracellular traps promote macrophage inflammation and impair atherosclerosis resolution in diabetic mice. *JCI Insight*.

[30] Gupta S., Kaplan M. J. (2016). The role of neutrophils and NETosis in autoimmune and renal diseases. *Nature Reviews. Nephrology*.

[31] Zhu W., Li Y.-Y., Zeng H.-X. (2021). Carnosine alleviates podocyte injury in diabetic nephropathy by targeting caspase-1-mediated pyroptosis. *International Immunopharmacology*.

[32] Lee Y. H., Seo J.-W., Kim M. (2021). Urinary mRNA signatures as predictors of renal function decline in patients with biopsy-proven diabetic kidney disease. *Frontiers in Endocrinology*.

[33] Wang S., Chen S., Gao Y., Zhou H. (2023). Bioinformatics led discovery of biomarkers related to immune infiltration in diabetes nephropathy. *Medicine (Baltimore)*.

[34] Lin J., Weng M., Zheng J. (2024). Identification and validation of voltage-dependent anion channel 1-related genes and immune cell infiltration in diabetic nephropathy. *Journal of Diabetes Investigation*.

[35] Papayannopoulos V. J. N. R. I. (2018). Neutrophil extracellular traps in immunity and disease. *Nature Reviews. Immunology*.

[36] Nakazawa D., Kumar S. V., Marschner J. (2017). Histones and neutrophil extracellular traps enhance tubular necrosis and remote organ injury in ischemic AKI. *Journal of the American Society of Nephrology*.

[37] Jorch S. K., Kubes P. (2017). An emerging role for neutrophil extracellular traps in noninfectious disease. *Nature Medicine*.

[38] Liu C., Zhou Y., Tu Q., Li J., Yang Z. (2023). Alpha-linolenic acid pretreatment alleviates NETs-induced alveolar macrophage pyroptosis by inhibiting pyrin inflammasome activation in a mouse model of sepsis-induced ALI/ARDS. *Frontiers in Immunology*.

