# Supplementary material for: NET-Related Gene as Potential Diagnostic Biomarkers for Diabetic Tubulointerstitial Injury
Source: J Diabetes Res. 2024 May 10;2024:4815488. doi: 10.1155/2024/4815488 (PMC11101254; doi:10.1155/2024/4815488)
Supplement: Supporting Information — Additional supporting information can be found online in the Supporting Information section. Supporting Information S1: R language for DEGs. Table S2: DEGs identified in the gene expression microarray study. Table S3: GO enrichment analysis of DEG. Table S4: KEGG enrichment analysis of DEG. Table S5: DEG related to NETs identified through machine learning. [file 4815488.f1.zip › Supplementary table S2.docx]

| Supplementary table S2: Differentially expressed genes identified in the gene expression microarray study | | | | | | |
| --- | --- | --- | --- | --- | --- | --- |
| id | logFC | AveExpr | t | P.Value | adj.P.Val | B |
| CXCL6 | 2.789249909 | 0.015036152 | 8.680593955 | 5.33E-10 | 5.68E-06 | 12.67309174 |
| PLK2 | 1.878835586 | 0.154420125 | 8.682320893 | 9.06E-10 | 5.68E-06 | 12.16874099 |
| QPCT | 2.024227085 | -0.079676608 | 8.254291627 | 1.33E-09 | 5.68E-06 | 11.82929618 |
| PTPRC | 1.850297275 | -0.286642869 | 8.285285389 | 1.96E-09 | 6.28E-06 | 11.46251128 |
| LTF | 2.269058132 | 0.622314773 | 8.901783844 | 2.49E-09 | 6.39E-06 | 11.14002808 |
| EVI2B | 1.961555955 | 0.025770263 | 8.236357738 | 3.68E-09 | 7.86E-06 | 10.87042266 |
| CCR2 | 1.136827196 | 0.044571414 | 7.59881908 | 6.92E-09 | 1.27E-05 | 10.28552328 |
| MS4A4A | 1.575446515 | 0.272926262 | 7.379571606 | 2.77E-08 | 2.95E-05 | 8.996031605 |
| CASP1 | 1.855288368 | -0.276154767 | 7.171988631 | 2.85E-08 | 2.95E-05 | 8.961273301 |
| TFAP4 | -1.166509861 | 0.010751106 | -7.103487713 | 2.95E-08 | 2.95E-05 | 8.924325253 |
| GTF2E1 | 1.109323806 | -0.347230949 | 7.06473023 | 3.30E-08 | 3.03E-05 | 8.816680512 |
| TPPP | -1.067865755 | 0.002474505 | -6.907188808 | 5.26E-08 | 4.50E-05 | 8.377575229 |
| KCNQ1DN | -1.333502692 | 0.102675452 | -6.983914717 | 5.78E-08 | 4.63E-05 | 8.265651202 |
| COL4A1 | 2.11338553 | -0.490047587 | 7.137434779 | 6.48E-08 | 4.89E-05 | 8.205903716 |
| GZMA | 1.549415799 | 0.133459738 | 6.9735388 | 1.01E-07 | 7.17E-05 | 7.794508742 |
| CRYBB3 | -1.507863833 | -0.180252011 | -6.62290466 | 1.23E-07 | 8.28E-05 | 7.579394969 |
| COL3A1 | 1.841751566 | -0.072088177 | 7.245736874 | 1.46E-07 | 8.92E-05 | 7.454264933 |
| GAD1 | -1.223755095 | 0.035733859 | -6.511534182 | 1.71E-07 | 9.54E-05 | 7.264877417 |
| MACF1 | 1.004369178 | -0.310570039 | 6.547463367 | 2.03E-07 | 0.000108316 | 7.118649393 |
| EFHC1 | 1.480636665 | -0.526067974 | 6.485168184 | 2.43E-07 | 0.000122087 | 6.94857516 |
| MYOF | 1.633321854 | -0.459238585 | 6.431833283 | 2.48E-07 | 0.000122087 | 6.923148266 |
| JCHAIN | 2.204591602 | 0.270200628 | 6.607297622 | 2.72E-07 | 0.000129009 | 6.863585264 |
| DUSP9 | -1.489880225 | 0.135444285 | -6.380497636 | 2.88E-07 | 0.000131921 | 6.773304059 |
| KLK1 | -1.574451256 | 0.234177706 | -6.272841373 | 3.50E-07 | 0.0001497 | 6.587859107 |
| STRA6 | -1.930659769 | 0.138025785 | -6.487571165 | 3.77E-07 | 0.000153007 | 6.512528114 |
| CORT | -1.142206563 | -0.021624089 | -6.244124068 | 3.82E-07 | 0.000153007 | 6.50616856 |
| PRSS3P2 | -1.209823703 | -0.005698486 | -6.233693844 | 3.94E-07 | 0.000153102 | 6.476486925 |
| MNDA | 2.111895701 | -0.272520458 | 6.366711962 | 4.53E-07 | 0.000170841 | 6.375869008 |
| ZNF358 | -1.508053307 | -0.0222067 | -6.179950529 | 5.22E-07 | 0.000175272 | 6.214671095 |
| PXDN | 2.329876718 | -0.508904352 | 6.305051848 | 5.39E-07 | 0.000175272 | 6.212894027 |
| GRIN2A | -1.742382415 | -0.252217303 | -6.14069359 | 5.21E-07 | 0.000175272 | 6.211580999 |
| TCL6 | -1.011464326 | 0.068775278 | -6.121726051 | 5.52E-07 | 0.000175272 | 6.157500618 |
| HSD17B1 | -1.218995016 | 0.02002188 | -6.087867228 | 6.86E-07 | 0.000205341 | 5.957341097 |
| BRD7 | 1.501460682 | -0.376951119 | 6.048448668 | 6.89E-07 | 0.000205341 | 5.948420626 |
| VCY | -1.213352727 | -0.037476327 | -6.028513678 | 7.31E-07 | 0.000213117 | 5.89150197 |
| HLA-DPA1 | 1.545728465 | 0.066828903 | 6.593143173 | 8.59E-07 | 0.000220276 | 5.820783395 |
| AGR2 | 1.374145673 | 0.178050317 | 6.073321127 | 8.06E-07 | 0.000219779 | 5.816707826 |
| F11 | -2.023545779 | 0.10283028 | -6.036720276 | 7.98E-07 | 0.000219779 | 5.814214329 |
| HSF4 | -1.19952527 | 0.009184501 | -5.984645345 | 8.35E-07 | 0.000220276 | 5.766195178 |
| LIM2 | -1.041738341 | 0.039294905 | -5.91519319 | 1.03E-06 | 0.000244512 | 5.567674385 |
| HOXC8 | -1.351030424 | -0.031158912 | -5.959598097 | 1.12E-06 | 0.000257307 | 5.494241791 |
| PTGER3 | -1.292835485 | 0.152099562 | -5.80901142 | 1.42E-06 | 0.000310279 | 5.263902699 |
| IGFALS | -1.294019619 | -0.022232106 | -5.870950427 | 1.46E-06 | 0.000310279 | 5.250807649 |
| C2ORF72 | -1.352407499 | -0.045072116 | -5.796072742 | 1.48E-06 | 0.000310279 | 5.226869327 |
| PROC | -1.924619673 | 0.486753172 | -6.119485599 | 1.62E-06 | 0.00032681 | 5.157534789 |
| PNMA3 | -1.000924742 | 0.073687044 | -5.763037831 | 1.63E-06 | 0.00032681 | 5.132302218 |
| SCRN1 | 1.66986336 | -0.373455491 | 6.069898679 | 1.84E-06 | 0.000333522 | 5.092012125 |
| SLAMF8 | 1.412869606 | -0.45429813 | 5.746409525 | 1.72E-06 | 0.00032828 | 5.084694596 |
| BTN3A3 | 1.093563656 | -0.249743978 | 5.733456511 | 1.78E-06 | 0.000333522 | 5.04760675 |
| MLX | -1.043719591 | -0.048871857 | -5.721329 | 1.85E-06 | 0.000333522 | 5.012880542 |
| POLR2C | -1.397793713 | -0.181104004 | -5.717381488 | 1.87E-06 | 0.000333522 | 5.001576753 |
| GRAMD1B | -1.432355366 | 0.050322402 | -5.738474767 | 1.94E-06 | 0.000336245 | 4.977452905 |
| CYP11B2 | -1.304026088 | -0.085776156 | -5.71881791 | 2.06E-06 | 0.000342508 | 4.922205556 |
| MID1 | 1.338044112 | -0.222836454 | 5.773244636 | 2.16E-06 | 0.000350948 | 4.892452997 |
| LYZ | 2.274513001 | 0.491319081 | 6.642818546 | 2.55E-06 | 0.000378199 | 4.796961493 |
| DNMT1 | 1.313735493 | -0.320331728 | 5.64507536 | 2.33E-06 | 0.000364466 | 4.794500663 |
| SLC16A7 | -1.540116209 | 0.221294862 | -5.627440525 | 2.46E-06 | 0.000376204 | 4.743991554 |
| PAK4 | -1.204949373 | -0.189564931 | -5.613291749 | 2.57E-06 | 0.000378199 | 4.703466427 |
| CD53 | 1.766950356 | -0.103316708 | 5.719672952 | 2.82E-06 | 0.000405753 | 4.651550057 |
| PRKCB | 1.209356312 | -0.17992604 | 5.564650207 | 2.97E-06 | 0.000418914 | 4.564146148 |
| SETX | 1.230988647 | -0.258851628 | 5.590432383 | 3.02E-06 | 0.000420448 | 4.561195416 |
| CX3CR1 | 2.125974475 | -0.36210835 | 5.631578185 | 3.26E-06 | 0.00042885 | 4.505393087 |
| HLA-DRB5 | 1.761281919 | -0.28268347 | 5.596369991 | 3.26E-06 | 0.00042885 | 4.494374701 |
| LOC100289473 | -1.255541235 | -0.119727854 | -5.53916881 | 3.21E-06 | 0.00042885 | 4.491164217 |
| HDAC9 | 1.698787481 | -0.39306758 | 5.56263059 | 3.28E-06 | 0.00042885 | 4.481029684 |
| FCGR3B | 1.631697057 | -0.143584957 | 5.720235082 | 3.57E-06 | 0.000444383 | 4.451252315 |
| MFAP1 | 1.076698232 | -0.364015876 | 5.523070109 | 3.37E-06 | 0.000436692 | 4.445057424 |
| CLDN8 | -1.664085861 | 0.257761084 | -5.565965308 | 3.57E-06 | 0.000444383 | 4.41099173 |
| MMP7 | 2.052015471 | 0.345315385 | 5.828737018 | 4.00E-06 | 0.000485375 | 4.374437596 |
| EFNB2 | 1.763180498 | -0.340916487 | 5.850276973 | 4.41E-06 | 0.000504693 | 4.294209876 |
| ELK1 | -1.023342446 | -0.048179218 | -5.462320403 | 4.05E-06 | 0.000485573 | 4.271091476 |
| FXR2 | -1.032168601 | -0.065076471 | -5.446756916 | 4.25E-06 | 0.00049913 | 4.226530517 |
| FEV | -1.041861126 | -0.128326633 | -5.441491671 | 4.32E-06 | 0.00049913 | 4.211456054 |
| PRDM14 | -1.184324913 | -0.101900626 | -5.405151839 | 4.82E-06 | 0.00052791 | 4.107427867 |
| PRB4 | -1.449941953 | -0.184787351 | -5.394685917 | 4.97E-06 | 0.000537118 | 4.077472353 |
| ENC1 | 1.293759622 | -0.376688749 | 5.39383057 | 4.98E-06 | 0.000537118 | 4.075024286 |
| CD47 | 1.124884475 | -0.416688733 | 5.38277913 | 5.15E-06 | 0.000546194 | 4.043395667 |
| CSF2RB | 2.230231604 | -0.223696035 | 5.625048109 | 5.93E-06 | 0.000608709 | 3.994315259 |
| ART1 | -1.068262255 | 0.054391879 | -5.359643777 | 5.53E-06 | 0.000576251 | 3.977192738 |
| KLHL21 | -1.980866074 | -0.273225456 | -5.352911597 | 5.64E-06 | 0.000583359 | 3.957930771 |
| NLGN4X | 1.066040509 | 0.134318536 | 5.345151585 | 6.83E-06 | 0.000657203 | 3.795760821 |
| FCN1 | 1.273075644 | -0.245531118 | 5.309760906 | 6.97E-06 | 0.000657203 | 3.767308578 |
| GML | -1.274681551 | -0.231354195 | -5.277132468 | 7.09E-06 | 0.000663923 | 3.741203047 |
| ANXA2 | 1.675208988 | 0.09262542 | 5.873803501 | 8.33E-06 | 0.000728263 | 3.736878506 |
| DLGAP2 | -1.057657187 | 0.026895659 | -5.274415002 | 7.15E-06 | 0.000664547 | 3.73343453 |
| SH2B3 | 1.158776835 | -0.132902042 | 5.346227106 | 7.45E-06 | 0.000672953 | 3.724306274 |
| ADCY7 | 1.752357932 | -0.421895589 | 5.367132304 | 7.71E-06 | 0.000686458 | 3.705515407 |
| MST1 | -1.338797101 | 0.148869573 | -5.264000593 | 7.38E-06 | 0.000671199 | 3.703664905 |
| GPR52 | -1.039152611 | -0.017197455 | -5.267685916 | 7.90E-06 | 0.000698826 | 3.648640939 |
| EVI2A | 1.97147146 | -0.074816846 | 5.438350577 | 8.66E-06 | 0.000730039 | 3.626978645 |
| EGF | -2.934154416 | 0.205346144 | -5.319789807 | 8.82E-06 | 0.000730039 | 3.572767404 |
| SOD2 | 1.183480152 | 0.009661134 | 5.211899705 | 8.64E-06 | 0.000730039 | 3.554796229 |
| OAS1 | 1.114407029 | -0.354284624 | 5.198914266 | 8.98E-06 | 0.000733745 | 3.517710058 |
| CLEC2B | 1.450143791 | -0.153869175 | 5.561479942 | 1.05E-05 | 0.000793754 | 3.490254033 |
| GFRA3 | -1.064391837 | -0.099905781 | -5.178634654 | 9.55E-06 | 0.00076741 | 3.459806839 |
| POU3F1 | -1.06646641 | 0.070029105 | -5.178616053 | 9.55E-06 | 0.00076741 | 3.459753738 |
| PKLR | -1.702121618 | -0.039062465 | -5.243410598 | 1.00E-05 | 0.000775573 | 3.446865983 |
| MARCKSL1 | 1.881251292 | -0.355847235 | 5.210939846 | 1.01E-05 | 0.000778831 | 3.422655765 |
| CAST | 1.319534488 | -0.637173032 | 5.164697107 | 9.96E-06 | 0.000774136 | 3.420022804 |
| AKAP3 | -1.129368025 | -0.170215837 | -5.179834032 | 1.03E-05 | 0.000783724 | 3.406687066 |
| COL15A1 | 1.430965095 | 0.071649895 | 5.220723311 | 1.07E-05 | 0.0007938 | 3.380615814 |
| PON3 | -1.450133034 | 0.105282629 | -5.167668699 | 1.06E-05 | 0.000793754 | 3.372532953 |
| THY1 | -2.252602871 | 0.04744475 | -5.243734467 | 1.10E-05 | 0.000794371 | 3.372194477 |
| HLA-DRB1 | 1.635959351 | -0.299474191 | 5.271813858 | 1.11E-05 | 0.000796676 | 3.36832593 |
| FUZ | -1.321749361 | -0.097680514 | -5.144183848 | 1.06E-05 | 0.000793754 | 3.361485884 |
| ITGB2 | 1.463188522 | -0.121551169 | 5.185256161 | 1.09E-05 | 0.000794371 | 3.351296295 |
| ASNS | 1.847074392 | -0.344000558 | 5.403290512 | 1.21E-05 | 0.000826926 | 3.340671601 |
| TRBC1 | 1.316279651 | -0.040317576 | 5.154776579 | 1.11E-05 | 0.000796676 | 3.330450947 |
| NSMAF | 1.801918348 | -0.710247733 | 5.110254383 | 1.17E-05 | 0.000822579 | 3.264711741 |
| DGCR5 | -1.698910101 | -0.053241623 | -5.102339606 | 1.20E-05 | 0.000826926 | 3.242146029 |
| ANXA2P2 | 1.15191009 | 0.174126946 | 5.567957275 | 1.41E-05 | 0.000897154 | 3.240543301 |
| YIF1B | -1.04274212 | -0.044198222 | -5.101335477 | 1.21E-05 | 0.000826926 | 3.239283419 |
| GRIP2 | -1.095733492 | 0.088771362 | -5.164986095 | 1.26E-05 | 0.000855061 | 3.238974424 |
| JAK1 | 1.591039361 | -0.227723918 | 5.134539224 | 1.38E-05 | 0.000888978 | 3.144708745 |
| HAAO | -1.370406497 | 0.011996658 | -5.081172393 | 1.38E-05 | 0.000888978 | 3.129864966 |
| APOC4 | -1.415502345 | -0.001357071 | -5.056692196 | 1.38E-05 | 0.000888978 | 3.112071798 |
| LCP2 | 1.25983552 | -0.322659314 | 5.053697967 | 1.39E-05 | 0.000889639 | 3.103543987 |
| IFI44 | 1.814222274 | -0.746991428 | 5.06531521 | 1.44E-05 | 0.000907702 | 3.078693806 |
| KRT19 | 1.597941809 | 0.14755014 | 5.123025252 | 1.55E-05 | 0.000950298 | 3.049751979 |
| STUM | -1.527729172 | -0.120256495 | -5.029041203 | 1.50E-05 | 0.000933358 | 3.033341045 |
| INPP5J | -1.219688721 | 0.035505477 | -5.0040373 | 1.62E-05 | 0.00097785 | 2.962190373 |
| INO80B | -1.020708069 | -0.02904034 | -4.99496403 | 1.66E-05 | 0.000991681 | 2.936382154 |
| KCNA2 | -1.344861445 | -0.049082018 | -4.991095916 | 1.68E-05 | 0.000991681 | 2.925381344 |
| LINC01558 | -1.446038194 | -0.105126368 | -4.990097056 | 1.69E-05 | 0.000991681 | 2.922540785 |
| PDE1B | -1.183927178 | -0.127899994 | -4.979198929 | 1.74E-05 | 0.001015421 | 2.891553283 |
| RCN1 | 1.442974941 | -0.014448739 | 5.238456897 | 2.09E-05 | 0.001162812 | 2.839861022 |
| OTUB1 | -1.014104805 | -0.089281947 | -4.959983665 | 1.85E-05 | 0.001061365 | 2.836937955 |
| RPAP3 | 1.092432932 | 0.048619617 | 4.958193639 | 1.86E-05 | 0.00106233 | 2.831851572 |
| NMI | 1.904896572 | -0.519955178 | 4.9769405 | 2.02E-05 | 0.001142642 | 2.773343932 |
| ACSM5 | -1.682540896 | 0.283239174 | -5.069389827 | 2.15E-05 | 0.001177275 | 2.767084298 |
| SYNC | 1.04622085 | -0.161442151 | 4.973526086 | 2.04E-05 | 0.001145723 | 2.763886791 |
| PALM | -1.261735134 | 0.0635354 | -4.956291332 | 2.14E-05 | 0.001177275 | 2.730403003 |
| ESRP2 | -1.426632275 | -0.037438516 | -4.926511412 | 2.18E-05 | 0.001185327 | 2.696898047 |
| PSMB8 | 1.678838563 | -0.217510729 | 5.121436572 | 2.52E-05 | 0.001291792 | 2.649273277 |
| SORD | -1.791634845 | 0.381149856 | -5.053627363 | 2.46E-05 | 0.001278517 | 2.638882577 |
| CSTF3 | 1.2516205 | -0.415965109 | 4.905878144 | 2.32E-05 | 0.001234041 | 2.631088433 |
| PRRX2 | -1.374135273 | 0.038583588 | -4.913923619 | 2.43E-05 | 0.001275011 | 2.61400782 |
| IQGAP1 | 1.580538527 | -0.678044184 | 4.868983778 | 2.43E-05 | 0.001275011 | 2.57868037 |
| CDA | -1.511037664 | -0.213314513 | -4.883488406 | 2.48E-05 | 0.001281571 | 2.576723025 |
| TLR7 | 1.188775339 | -0.00087734 | 4.923964799 | 2.53E-05 | 0.001293827 | 2.569190477 |
| CFHR1 | 1.831141002 | -0.072634449 | 5.363358945 | 3.15E-05 | 0.001473812 | 2.520862062 |
| PXMP2 | -1.622417965 | 0.167325778 | -4.907369764 | 2.86E-05 | 0.00139114 | 2.485208799 |
| ITPKC | -1.25149854 | 0.054043288 | -4.844042765 | 2.79E-05 | 0.001374076 | 2.466659514 |
| TNFRSF17 | 1.09367046 | 0.039695851 | 4.828736146 | 2.74E-05 | 0.001355271 | 2.464681415 |
| LYN | 1.400049717 | -0.297655925 | 4.864303418 | 2.81E-05 | 0.001378428 | 2.461718394 |
| OR7E12P | -1.417447064 | 0.103760879 | -4.851683348 | 2.91E-05 | 0.001408716 | 2.443205624 |
| BEST1 | -1.215725962 | -0.081867686 | -4.845757063 | 2.96E-05 | 0.001417318 | 2.426955146 |
| DMTN | -1.042903866 | -0.134020856 | -4.810213385 | 3.08E-05 | 0.001457258 | 2.37236407 |
| CYP46A1 | -1.901350561 | 0.345991179 | -4.94421904 | 3.32E-05 | 0.001518852 | 2.367568599 |
| LYPD3 | -1.325803272 | -0.131920177 | -4.793772379 | 3.04E-05 | 0.001449018 | 2.365770282 |
| RNF34 | 1.251143062 | -0.310741038 | 4.790349536 | 3.07E-05 | 0.001457258 | 2.356093513 |
| NELL1 | -2.727424901 | -0.707918753 | -4.835288112 | 3.27E-05 | 0.001518852 | 2.350035967 |
| BDKRB2 | -1.215686422 | 0.208156768 | -4.857659272 | 3.30E-05 | 0.001518852 | 2.344929115 |
| ATXN7L1 | -1.169211787 | -0.04631533 | -4.778357582 | 3.18E-05 | 0.001484361 | 2.322200005 |
| CXCL1 | 1.420927036 | -0.065360484 | 4.892118473 | 3.51E-05 | 0.001567425 | 2.302984193 |
| CYP1B1 | 1.002840784 | -0.159045279 | 4.918210824 | 3.56E-05 | 0.001576544 | 2.301388406 |
| HTR1B | -1.221193507 | -0.106415896 | -4.777400851 | 3.39E-05 | 0.001537797 | 2.280994163 |
| CALML3 | -1.417861765 | -0.180901434 | -4.761542051 | 3.35E-05 | 0.001522232 | 2.274697626 |
| CGA | -2.547996449 | -0.149514082 | -4.803194354 | 3.59E-05 | 0.001576544 | 2.263744619 |
| ADGRD2 | -1.115025454 | -0.082008607 | -4.784055911 | 3.55E-05 | 0.001576544 | 2.257907432 |
| EPN1 | -1.302558628 | -0.104379035 | -4.750054062 | 3.46E-05 | 0.001561864 | 2.242261686 |
| PRC1 | 1.274925675 | -0.294636667 | 4.777585615 | 3.62E-05 | 0.001582422 | 2.222373864 |
| CLPTM1 | -1.240626299 | -0.009436952 | -4.738836496 | 3.58E-05 | 0.001576544 | 2.210602524 |
| RBKS | -1.223215586 | -0.092895065 | -4.746851889 | 3.71E-05 | 0.00160928 | 2.19601258 |
| SGK2 | -1.318140677 | -0.001603055 | -4.724657396 | 3.74E-05 | 0.001610105 | 2.170604137 |
| RNF208 | -1.054083222 | -0.045319671 | -4.727955602 | 3.93E-05 | 0.0016624 | 2.143489551 |
| TAP1 | 1.401503741 | -0.441091845 | 4.765403964 | 4.00E-05 | 0.001688763 | 2.137202437 |
| LTB4R2 | -1.352071882 | -0.054289802 | -4.716772327 | 4.06E-05 | 0.001701396 | 2.112421121 |
| HMGXB4 | 1.132631431 | -0.53943599 | 4.693234445 | 4.11E-05 | 0.001703916 | 2.082039749 |
| HMX1 | -1.153100934 | -0.038063394 | -4.717231673 | 4.31E-05 | 0.001755605 | 2.075145002 |
| C1GALT1C1 | 1.836721973 | -0.827699493 | 4.69953244 | 4.27E-05 | 0.001744648 | 2.054591177 |
| MAPKAPK3 | -1.614126724 | -0.183230947 | -4.680289707 | 4.27E-05 | 0.001744648 | 2.045587542 |
| POU2AF1 | 1.824674795 | -0.011058257 | 4.819067768 | 4.66E-05 | 0.001868105 | 2.043052583 |
| SPON2 | 2.071191776 | -0.289314541 | 4.784501568 | 4.72E-05 | 0.00187691 | 2.023650032 |
| ATP2C2 | -1.098469069 | 0.154616477 | -4.665178873 | 4.73E-05 | 0.00187691 | 1.969248665 |
| TNFSF8 | -1.050141386 | -0.08420344 | -4.64551678 | 4.73E-05 | 0.00187691 | 1.947763318 |
| C3ORF18 | -1.007209561 | -0.167087727 | -4.651414881 | 4.92E-05 | 0.001927555 | 1.931099595 |
| AFDN-DT | -1.248579527 | -0.002166348 | -4.631888063 | 4.93E-05 | 0.001927555 | 1.909461745 |
| ATP1B2 | -1.065481793 | -0.143849331 | -4.653276931 | 5.20E-05 | 0.001995161 | 1.900581778 |
| CLEC4A | 1.291845525 | -0.235891465 | 4.63814865 | 5.12E-05 | 0.001977581 | 1.883868093 |
| HLA-B | 2.37029341 | -0.693905706 | 4.756630329 | 5.52E-05 | 0.002052128 | 1.88330408 |
| SSTR2 | -1.083502906 | -0.085479067 | -4.61811802 | 5.14E-05 | 0.001978119 | 1.87078598 |
| PDIA6 | 1.602519726 | -0.473255995 | 4.641549353 | 5.38E-05 | 0.002034044 | 1.848145574 |
| FGF9 | -1.723515621 | -0.122860052 | -4.685779619 | 5.77E-05 | 0.002108928 | 1.840734643 |
| ACSL4 | 1.348986556 | -0.194582023 | 4.61931275 | 5.41E-05 | 0.002041234 | 1.831562636 |
| ME1 | 1.574756073 | -0.604683259 | 4.627340076 | 5.60E-05 | 0.002065138 | 1.809155926 |
| POGLUT2 | 1.897192505 | -0.404982979 | 4.595834047 | 5.49E-05 | 0.002046215 | 1.8082472 |
| EDA2R | -1.094340141 | -0.084889224 | -4.590532947 | 5.89E-05 | 0.002127567 | 1.751721812 |
| TPM1 | 1.650419994 | -0.515635269 | 4.6226137 | 6.04E-05 | 0.002145418 | 1.749678942 |
| PTPRE | 1.892430526 | -0.442905872 | 4.589618711 | 5.91E-05 | 0.002127567 | 1.749187117 |
| OR7E47P | -1.037871974 | 0.198749958 | -4.593836942 | 6.18E-05 | 0.002172119 | 1.738683488 |
| HLA-DPB1 | 1.949353748 | -0.459258289 | 4.653488764 | 6.31E-05 | 0.002188846 | 1.731496979 |
| FHL1 | 1.896588215 | -0.756664478 | 4.566179429 | 6.00E-05 | 0.00213721 | 1.725121509 |
| CES3 | -1.716165991 | 0.441178066 | -4.683286905 | 6.74E-05 | 0.002269734 | 1.720299869 |
| SH3BP1 | -1.311173196 | 0.002649982 | -4.584809278 | 6.34E-05 | 0.002192258 | 1.714125458 |
| ATIC | 1.045909795 | -0.261753219 | 4.573370426 | 6.20E-05 | 0.002172119 | 1.704155729 |
| SDC3 | 1.05719859 | -0.315347764 | 4.573154781 | 6.20E-05 | 0.002172119 | 1.70355829 |
| CASP3 | 1.395572933 | -0.31690858 | 4.557091884 | 6.16E-05 | 0.002172119 | 1.699671015 |
| CD14 | 1.330512427 | -0.185906627 | 4.561130389 | 6.42E-05 | 0.002213983 | 1.681380844 |
| CRACR2B | -1.146509528 | -0.112869495 | -4.53968318 | 6.49E-05 | 0.002224001 | 1.650947246 |
| FUT3 | -1.632245645 | -0.235294558 | -4.533575622 | 6.96E-05 | 0.00231388 | 1.605357157 |
| NENF | -2.134076125 | -0.319144865 | -4.533533431 | 6.97E-05 | 0.00231388 | 1.605240824 |
| SRGN | 1.098100177 | 0.126908293 | 4.747210558 | 8.13E-05 | 0.002482813 | 1.58042923 |
| TG | -1.014806893 | 0.016163128 | -4.513275645 | 7.02E-05 | 0.00231856 | 1.577116505 |
| ARSF | -2.101982169 | 0.110786999 | -4.601431043 | 7.83E-05 | 0.00244684 | 1.575897215 |
| VCAN | 1.674563982 | 0.057212605 | 4.634274576 | 7.70E-05 | 0.002425279 | 1.570583224 |
| KIR2DL5A | -1.111550541 | -0.050380794 | -4.507734628 | 7.13E-05 | 0.002332915 | 1.56163715 |
| MAGED4B | 1.290195852 | -0.148396933 | 4.533867397 | 7.35E-05 | 0.002369481 | 1.55320003 |
| SPTBN4 | -1.01515793 | 0.046920196 | -4.502995001 | 7.23E-05 | 0.002359918 | 1.548399996 |
| CEL | -1.726617676 | 0.035156373 | -4.507969085 | 7.51E-05 | 0.002401398 | 1.534793505 |
| LY75 | 2.062619532 | -0.777459116 | 4.55837334 | 7.72E-05 | 0.002425279 | 1.529589577 |
| NNAT | -1.212234142 | -0.091533129 | -4.490124987 | 7.51E-05 | 0.002401398 | 1.512471899 |
| EVX1 | -1.792349843 | -0.333227387 | -4.485579368 | 7.62E-05 | 0.002417286 | 1.499787983 |
| CYP4F12 | -1.357999646 | 0.109749128 | -4.505844995 | 7.98E-05 | 0.002464884 | 1.499691228 |
| HLA-DRA | 2.249087988 | -0.574297049 | 4.58223024 | 8.26E-05 | 0.002514683 | 1.492075094 |
| SLC25A44 | -1.734822484 | -0.131881629 | -4.510979913 | 7.86E-05 | 0.00244684 | 1.490676204 |
| SECISBP2L | -1.154402145 | -0.22580656 | -4.510044283 | 7.88E-05 | 0.00244684 | 1.488121572 |
| SERPING1 | 1.393132086 | -0.240547491 | 4.525779894 | 7.98E-05 | 0.002464884 | 1.487906452 |
| HTR2B | 1.474648373 | -0.074015774 | 4.555632094 | 8.30E-05 | 0.002522987 | 1.473885524 |
| UQCRQ | -1.73782122 | -0.202460636 | -4.502107322 | 8.54E-05 | 0.002551278 | 1.457779652 |
| LAMA5 | 1.194591729 | -0.409374532 | 4.527294136 | 8.44E-05 | 0.002539163 | 1.446768032 |
| FAM149A | -1.469357618 | -0.003654476 | -4.467091605 | 8.46E-05 | 0.002541704 | 1.422324083 |
| PDIA5 | 1.38448265 | -0.476335698 | 4.49610074 | 8.68E-05 | 0.00257753 | 1.40786589 |
| C1GALT1 | -1.201251875 | -0.053934505 | -4.43772445 | 8.77E-05 | 0.002582388 | 1.366440202 |
| LAMP3 | 1.019256561 | 0.077649614 | 4.437199344 | 8.79E-05 | 0.002582388 | 1.364978903 |
| CPA3 | 1.351283786 | 0.301290066 | 4.514085294 | 9.32E-05 | 0.00263225 | 1.36472949 |
| NFATC4 | -1.340810877 | -0.22923895 | -4.434610888 | 8.86E-05 | 0.002592714 | 1.357776188 |
| KCNK10 | -1.475620482 | -0.040062269 | -4.453169499 | 9.29E-05 | 0.00263225 | 1.357044012 |
| PSD3 | 1.721506652 | -0.534843062 | 4.509924998 | 9.43E-05 | 0.00263225 | 1.353806946 |
| SBSPON | 1.072726458 | -0.341315176 | 4.459130915 | 9.13E-05 | 0.002625985 | 1.349269177 |
| GNG7 | -1.18433022 | -0.065628761 | -4.425229252 | 9.11E-05 | 0.002625985 | 1.331679205 |
| CCDC186 | -1.469306616 | -0.085266408 | -4.43332418 | 9.34E-05 | 0.00263225 | 1.329587307 |
| MICB | 1.340819352 | -0.400849748 | 4.447079931 | 9.46E-05 | 0.00263225 | 1.327923959 |
| PER1 | -1.557623892 | -0.128688288 | -4.483950739 | 0.000101429 | 0.002743982 | 1.325995091 |
| PRKD2 | 1.085981354 | -0.210466108 | 4.418208754 | 9.30E-05 | 0.00263225 | 1.312159132 |
| DPYSL2 | 1.698206813 | -0.805645623 | 4.445382576 | 9.50E-05 | 0.00263225 | 1.311830017 |
| CCL5 | 1.735919084 | -0.409228119 | 4.413143486 | 9.44E-05 | 0.00263225 | 1.29808023 |
| NUP85 | 1.255005561 | -0.376216735 | 4.438926615 | 9.68E-05 | 0.002669879 | 1.294257713 |
| KBTBD4 | 1.468754785 | -0.631473092 | 4.41099201 | 9.50E-05 | 0.00263225 | 1.292101425 |
| LPGAT1 | 1.503288241 | -0.295731191 | 4.403711727 | 9.70E-05 | 0.002669879 | 1.271875423 |
| SPARC | 2.760680016 | -0.434390909 | 4.804496922 | 0.000128346 | 0.003128856 | 1.241509749 |
| KLK11 | -1.121146736 | 0.074358196 | -4.391954914 | 0.000100452 | 0.002734814 | 1.239230537 |
| POU5F1 | -1.016168084 | -0.010806867 | -4.388668964 | 0.000101431 | 0.002743982 | 1.230110465 |
| TIMM10 | -1.038935623 | 0.059279171 | -4.405643144 | 0.000106615 | 0.002828053 | 1.228636515 |
| DSCAM | -1.234890914 | -0.086469507 | -4.391223822 | 0.000105662 | 0.002823556 | 1.214191295 |
| KLRB1 | 1.498643057 | -0.266950976 | 4.377509268 | 0.000104825 | 0.00281208 | 1.199150024 |
| ESYT1 | 1.358720164 | -0.369009959 | 4.615665575 | 0.000124874 | 0.003092556 | 1.19645135 |
| CASP4 | 1.236275262 | -0.433819939 | 4.387734343 | 0.000106744 | 0.002828053 | 1.192094386 |
| HLA-A | 1.24324138 | -0.459765611 | 4.372703984 | 0.000106321 | 0.002828053 | 1.185824916 |
| XRCC5 | 1.009326997 | -0.267401199 | 4.371591138 | 0.00010667 | 0.002828053 | 1.18273952 |
| MMUT | -1.418910082 | -0.134503901 | -4.376854773 | 0.000110188 | 0.002890759 | 1.174865478 |
| PLAC8 | 1.686909057 | -0.353149375 | 4.407645974 | 0.000111831 | 0.002920599 | 1.169907126 |
| STBD1 | -1.737583437 | -0.307772312 | -4.35878247 | 0.000110774 | 0.002898892 | 1.147241808 |
| SFXN1 | -1.479259612 | 0.043975028 | -4.374366773 | 0.000116691 | 0.003004669 | 1.144295277 |
| MEX3C | 1.605801768 | -0.609702618 | 4.382043961 | 0.000114134 | 0.002968637 | 1.139670524 |
| ZNF444 | -1.13103792 | 0.086980019 | -4.354679756 | 0.000117549 | 0.003008111 | 1.114237039 |
| LOXL1 | 1.876034141 | -0.525578061 | 4.417861849 | 0.000121945 | 0.003054096 | 1.112496997 |
| TPSD1 | -1.096347621 | 0.042187992 | -4.342948717 | 0.000116062 | 0.002994498 | 1.10339813 |
| USP2 | -2.721804327 | -0.28706216 | -4.356804763 | 0.000122754 | 0.00306837 | 1.096995357 |
| GZMB | 1.000640655 | -0.220301744 | 4.336694922 | 0.000118219 | 0.003008111 | 1.086092984 |
| ZNF264 | 1.109167489 | 0.040570408 | 4.361224224 | 0.0001212 | 0.003047996 | 1.083201309 |
| C11ORF16 | -1.508647705 | -0.148071332 | -4.331520238 | 0.000120032 | 0.00304185 | 1.071778913 |
| TXNIP | 1.164361881 | -0.130754531 | 4.413650359 | 0.000131288 | 0.00317535 | 1.056123731 |
| AVP | -1.666935828 | -0.243261686 | -4.330809089 | 0.000126015 | 0.003100208 | 1.04905726 |
| CCL2 | 1.500106403 | -0.144691309 | 4.391770742 | 0.000131131 | 0.00317535 | 1.044255836 |
| WASF2 | 1.419944148 | -0.550002353 | 4.31727512 | 0.000125169 | 0.003092556 | 1.032398145 |
| ARAP2 | 1.490547055 | -0.358022558 | 4.338504587 | 0.000129402 | 0.003148625 | 1.021649974 |
| CASP8AP2 | 1.334988049 | -0.409927475 | 4.311327977 | 0.000127376 | 0.003111133 | 1.01596758 |
| ACO1 | -2.06958936 | -0.121462417 | -4.351549803 | 0.000138544 | 0.003296001 | 1.003558461 |
| COPG2IT1 | -1.194503811 | 0.001542119 | -4.304373409 | 0.000130006 | 0.003157331 | 0.996761557 |
| CCND2 | 1.432461439 | -0.524351274 | 4.29854431 | 0.000132252 | 0.003187717 | 0.980670209 |
| SRPK3 | -1.122476452 | -0.02443542 | -4.295868709 | 0.000139499 | 0.003305477 | 0.953814296 |
| FOXH1 | -1.162233095 | -0.043860936 | -4.288091125 | 0.000136375 | 0.003250429 | 0.951829015 |
| SPRY1 | 1.76396349 | -0.576032826 | 4.354351358 | 0.000145512 | 0.003398731 | 0.946508763 |
| MYO1D | 1.78061112 | -0.491195882 | 4.323907183 | 0.000141987 | 0.003335601 | 0.945529164 |
| PRODH | -1.447439766 | 0.091910186 | -4.318743218 | 0.000151939 | 0.003472937 | 0.944657725 |
| CASC3 | 1.011687347 | -0.468063816 | 4.295334848 | 0.000139715 | 0.003305477 | 0.939082466 |
| SLC12A3 | -1.74073593 | -0.105765834 | -4.306165168 | 0.000142029 | 0.003335601 | 0.934168556 |
| DCTD | 1.131939097 | -0.420435206 | 4.32494006 | 0.000149315 | 0.003464432 | 0.909949071 |
| RNASE6 | 1.196639742 | 0.028615108 | 4.352078895 | 0.000155454 | 0.003511273 | 0.897478808 |
| SLC66A1 | -1.158633444 | -0.104347666 | -4.26803723 | 0.00014464 | 0.003384528 | 0.896553382 |
| KDELR3 | 1.324287395 | -0.348302145 | 4.369288109 | 0.000158021 | 0.003542495 | 0.895871942 |
| AGMAT | -1.298354293 | -0.109535996 | -4.300420499 | 0.000151798 | 0.003472937 | 0.894735245 |
| ASB9 | -2.660079376 | -0.075898394 | -4.299448972 | 0.000160406 | 0.003576352 | 0.894672058 |
| ZGPAT | -2.521664069 | 0.034228883 | -4.320761372 | 0.000159743 | 0.003568619 | 0.894316864 |
| SLC4A1 | -1.919724153 | -0.093039359 | -4.276986191 | 0.000154458 | 0.003505513 | 0.882577999 |
| HLA-F | 1.343277081 | -0.335020048 | 4.343991122 | 0.000158939 | 0.003556858 | 0.876666394 |
| CISD1 | -1.104878375 | -0.033120149 | -4.252142785 | 0.000151539 | 0.003472937 | 0.852794533 |
| CLDN6 | -1.042630386 | -0.091499733 | -4.258349756 | 0.000155558 | 0.003511273 | 0.851765757 |
| ACSL5 | 1.901516108 | -0.780532179 | 4.261000681 | 0.000154366 | 0.003505513 | 0.845421059 |
| MAP4K4 | 1.164873345 | -0.296184028 | 4.249111949 | 0.000152891 | 0.003482272 | 0.844455639 |
| QTRT2 | 1.705492005 | -0.698987776 | 4.242661672 | 0.000155807 | 0.003511273 | 0.826714332 |
| LY96 | 1.605445424 | 0.11167129 | 4.414791153 | 0.000188504 | 0.003924009 | 0.79394015 |
| TNFAIP8 | 1.246526719 | 0.223981092 | 4.386507384 | 0.000186934 | 0.003910367 | 0.784600167 |
| HLA-G | 1.141497827 | -0.281616572 | 4.23575631 | 0.000166091 | 0.003659421 | 0.776684902 |
| HLA-E | 2.012910637 | -0.797026175 | 4.287446132 | 0.000175214 | 0.003763432 | 0.772124803 |
| RIMS1 | -1.156201334 | -0.095151754 | -4.225884147 | 0.00017091 | 0.003720852 | 0.763655439 |
| YWHAH | 1.315788315 | -0.105687493 | 4.218040592 | 0.000167449 | 0.003672519 | 0.759066399 |
| AMT | -1.167664246 | 0.034489873 | -4.217305838 | 0.000175209 | 0.003763432 | 0.740405063 |
| GJD2 | -1.195413766 | -0.036612974 | -4.207971237 | 0.000172452 | 0.003748061 | 0.731433377 |
| UBD | 1.267277983 | -0.012902318 | 4.242656412 | 0.000178861 | 0.003816197 | 0.728742534 |
| ELK2AP | -1.753361908 | -0.185876431 | -4.212935057 | 0.00017744 | 0.003798528 | 0.728563666 |
| FAM184A | -1.352622087 | 0.150150518 | -4.219714001 | 0.000190881 | 0.003928843 | 0.710266902 |
| RPS6KA2 | 1.220915931 | -0.368560581 | 4.202951193 | 0.000182642 | 0.00385289 | 0.687530443 |
| MOSPD3 | -1.284950615 | -0.250962731 | -4.188625712 | 0.00018248 | 0.00385289 | 0.678398971 |
| RAB31 | 2.061463848 | -0.604696579 | 4.364821563 | 0.000213896 | 0.004199958 | 0.675802167 |
| SNTA1 | -1.469981482 | -0.367230117 | -4.19013534 | 0.000189539 | 0.003927605 | 0.666849946 |
| CYFIP2 | -1.915776897 | 0.029438839 | -4.188358358 | 0.000190515 | 0.003927605 | 0.662044017 |
| ZBTB7B | -1.213557578 | 0.017199948 | -4.185972142 | 0.000200506 | 0.004075964 | 0.639287359 |
| BHLHE41 | 1.596437508 | -0.272792233 | 4.245200606 | 0.000208286 | 0.004147283 | 0.633087851 |
| FSTL1 | 2.522598182 | -0.809220237 | 4.300800742 | 0.000216968 | 0.004229602 | 0.628745955 |
| C3 | 1.684182051 | 0.456632639 | 4.26083902 | 0.000211782 | 0.004194047 | 0.621232378 |
| ELL2 | -1.274146746 | -0.154406361 | -4.160054265 | 0.000206746 | 0.004123015 | 0.585572927 |
| FAS | 1.056346438 | -0.122289736 | 4.151326632 | 0.000203452 | 0.004095546 | 0.576354323 |
| SLC2A4RG | -1.13631486 | 0.033768124 | -4.159148007 | 0.000226563 | 0.00431578 | 0.551364966 |
| CD48 | 2.074722088 | -0.455877866 | 4.171116953 | 0.000219027 | 0.004255431 | 0.538694312 |
| DPF1 | -1.388697897 | -0.256830525 | -4.137264622 | 0.000211957 | 0.004194047 | 0.537955498 |
| PON2 | 1.950372783 | -0.276551263 | 4.389082553 | 0.000263782 | 0.004678396 | 0.537847891 |
| UFD1 | 1.356966808 | -0.556398532 | 4.135992313 | 0.000212743 | 0.004194047 | 0.534483227 |
| EAF2 | -1.663647451 | 0.11534796 | -4.172113387 | 0.000229189 | 0.00433464 | 0.534210725 |
| TXLNA | 1.033840363 | -0.287640236 | 4.135746739 | 0.000212895 | 0.004194047 | 0.533813067 |
| ACADS | -1.03228665 | 0.207565838 | -4.134741508 | 0.000213519 | 0.004199317 | 0.531069968 |
| RASSF2 | 1.565576113 | -0.209957757 | 4.146968356 | 0.000224146 | 0.004307643 | 0.519754714 |
| CDH18 | -1.126764158 | -0.104838834 | -4.127653825 | 0.000217969 | 0.004241292 | 0.511734833 |
| NME5 | -1.069824413 | -0.061012973 | -4.12440785 | 0.000220037 | 0.004261429 | 0.502883313 |
| SULT2B1 | -1.06606472 | -0.117199559 | -4.123945316 | 0.000220333 | 0.004261429 | 0.501622198 |
| FKBP11 | 1.161924626 | -0.160704821 | 4.13035118 | 0.000225236 | 0.004310754 | 0.490931608 |
| ADGRL3 | -1.083352462 | 0.066305685 | -4.117922512 | 0.000224226 | 0.004307643 | 0.485204864 |
| GNPTAB | 1.20687999 | -0.692103203 | 4.11696478 | 0.000224851 | 0.004309817 | 0.482594916 |
| PHACTR2 | 1.09501004 | -0.425284157 | 4.126823288 | 0.000227537 | 0.004322536 | 0.481403839 |
| LHPP | -1.115335226 | 0.058563245 | -4.12506125 | 0.000238602 | 0.004426784 | 0.477243401 |
| SLC12A6 | -1.240731727 | 0.157491235 | -4.119442299 | 0.000232426 | 0.004346519 | 0.476113224 |
| CCDC103 | -1.12459823 | 0.025595294 | -4.11149687 | 0.000228454 | 0.004327124 | 0.467697807 |
| SHPK | -1.124034796 | -0.046405386 | -4.105452999 | 0.000232502 | 0.004346519 | 0.451238822 |
| CHST2 | 1.033479511 | -0.414685059 | 4.105413177 | 0.000232528 | 0.004346519 | 0.451130403 |
| LOC100129503 | -1.147170981 | 0.187777505 | -4.119523412 | 0.000253373 | 0.004585973 | 0.44772035 |
| RGS10 | 1.679102236 | -0.629217681 | 4.171625801 | 0.000254603 | 0.004591806 | 0.434783481 |
| SCNN1D | -1.039395748 | -0.077205867 | -4.098780186 | 0.000237052 | 0.004422185 | 0.433076038 |
| PIPOX | -1.10377169 | 0.292425534 | -4.147922899 | 0.000271586 | 0.004738152 | 0.427688377 |
| TCF15 | -1.568711486 | -0.17709554 | -4.10371596 | 0.000253564 | 0.004585973 | 0.420614213 |
| BPI | -1.354800843 | 0.129028504 | -4.095476397 | 0.000249021 | 0.004542246 | 0.411669536 |
| ECT2 | 1.585209915 | -0.326061056 | 4.122018964 | 0.000251596 | 0.004569717 | 0.408746365 |
| C1ORF105 | -1.139498834 | 0.126880286 | -4.09040079 | 0.00026336 | 0.004677374 | 0.385331497 |
| MAP1B | 1.154999567 | -0.3142234 | 4.200390059 | 0.000282101 | 0.004842541 | 0.383018645 |
| AP1S2 | 1.416105989 | -0.326084935 | 4.123651714 | 0.000262389 | 0.004673076 | 0.381083102 |
| C1QB | 1.130323837 | 0.25820369 | 4.134304015 | 0.000267588 | 0.004719782 | 0.375075089 |
| BORCS8-MEF2B | -1.329191213 | -0.33030643 | -4.077491408 | 0.000262231 | 0.004673076 | 0.363383695 |
| PRRG2 | -1.09421759 | 0.119947221 | -4.063070189 | 0.000262925 | 0.004676131 | 0.336038857 |
| HLA-DQA1 | 1.896539404 | -0.321324456 | 4.066151193 | 0.000270911 | 0.004736286 | 0.317935434 |
| ESPL1 | -1.646536309 | 0.185069302 | -4.093923001 | 0.000299063 | 0.005026058 | 0.31296665 |
| AGXT | -1.413107972 | 0.113759874 | -4.053854598 | 0.000270038 | 0.004732708 | 0.311041736 |
| TSC22D4 | -1.091958072 | -0.081730831 | -4.053691127 | 0.000270166 | 0.004732708 | 0.310598493 |
| CD1C | 1.107311973 | -0.014212092 | 4.06266781 | 0.000273633 | 0.004760917 | 0.30857294 |
| FURIN | -1.112120976 | -0.066973449 | -4.056223034 | 0.00027874 | 0.004823594 | 0.30636766 |
| LINC02249 | -1.163663482 | -0.115566265 | -4.04509621 | 0.000276973 | 0.004805986 | 0.287302158 |
| ZNF146 | 1.145545778 | -0.389198424 | 4.05280485 | 0.000281486 | 0.004838472 | 0.28207739 |
| FNDC3B | 1.509331016 | -0.522020958 | 4.111794493 | 0.000316224 | 0.005211926 | 0.255805722 |
| PVALB | -2.044810331 | 0.061102059 | -4.042732906 | 0.000301563 | 0.005046118 | 0.242591095 |
| ADM2 | -1.118189927 | 0.053636861 | -4.027804069 | 0.000291179 | 0.004951967 | 0.240482128 |
| GNRH2 | -1.122550983 | -0.230426149 | -4.022387415 | 0.000295773 | 0.004983835 | 0.225829893 |
| PRSS3 | -1.464403161 | -0.20370581 | -4.025867462 | 0.000304076 | 0.005063851 | 0.225153704 |
| HIST1H4E | -1.183283746 | -0.228582441 | -4.021538044 | 0.0002965 | 0.004989526 | 0.223532918 |
| SDC1 | -1.290461232 | 0.183852295 | -4.01575723 | 0.000301493 | 0.005046118 | 0.207904081 |
| PGAM2 | -1.811936987 | -0.328653795 | -4.015564124 | 0.000313177 | 0.005168427 | 0.182220551 |
| RAI2 | 1.362862024 | -0.453596744 | 4.053569385 | 0.000334146 | 0.005376097 | 0.167097978 |
| CRLF3 | 1.269158358 | -0.332179987 | 4.008870669 | 0.000331947 | 0.005360894 | 0.138199168 |
| PADI2 | -1.02608394 | 0.071562038 | -3.983894605 | 0.000330525 | 0.005344668 | 0.121899067 |
| SLC2A5 | -1.847564967 | -0.069563618 | -4.001416429 | 0.000339027 | 0.00541647 | 0.118455433 |
| HOXA1 | 1.385142773 | -0.410598239 | 4.047162251 | 0.000357122 | 0.005577807 | 0.118001334 |
| PAPSS1 | 2.108039201 | -0.765380249 | 4.016957744 | 0.000353024 | 0.005520523 | 0.103307111 |
| SLC1A6 | -1.24613005 | -0.085984777 | -3.973120306 | 0.000340946 | 0.005424254 | 0.092870106 |
| CNNM2 | -1.19729079 | 0.078134849 | -3.969465338 | 0.000344553 | 0.005454576 | 0.083028825 |
| PPP1R13L | -1.976332362 | -0.385951877 | -3.968202226 | 0.000345809 | 0.005460964 | 0.079628536 |
| TRAF4 | -1.027723814 | -0.012618506 | -3.961594397 | 0.000365373 | 0.005651604 | 0.053850875 |
| GSTA3 | -1.145120778 | 0.090640743 | -3.978871286 | 0.000376132 | 0.005725102 | 0.046655377 |
| AQP7 | -1.539119469 | -0.195882857 | -3.954892342 | 0.00035931 | 0.005596422 | 0.043821508 |
| SELL | 1.436974724 | -0.150201928 | 4.010237194 | 0.000394696 | 0.005905698 | 0.024449068 |
| SLC28A1 | -1.301904416 | 0.008066956 | -3.948699138 | 0.00037905 | 0.005758958 | 0.019592518 |
| CYP4A11 | -1.470993694 | 0.245316421 | -3.97282184 | 0.00039893 | 0.005934588 | 0.018583968 |
| SYT2 | -1.171060162 | -0.239441521 | -3.935580715 | 0.000379818 | 0.005763799 | -0.008056059 |
| ADAP1 | -1.035545614 | 0.017531832 | -3.930611975 | 0.000385277 | 0.005805411 | -0.021389131 |
| NAV2 | -1.245173385 | 0.211946641 | -3.948169374 | 0.000394077 | 0.00590333 | -0.022237238 |
| PPP1R1A | -1.113490892 | 0.208587469 | -3.929220314 | 0.000386819 | 0.005813169 | -0.02512242 |
| TXNDC15 | 1.251179362 | -0.531855445 | 3.920907895 | 0.000396159 | 0.005920679 | -0.04741153 |
| RAB11FIP3 | -2.252978423 | 0.072454018 | -3.95581814 | 0.000457249 | 0.006387038 | -0.051129995 |
| UPF3B | 1.45518758 | -0.635997266 | 3.943208123 | 0.00041555 | 0.006082879 | -0.060817774 |
| DPYD | 1.209383501 | -0.506036905 | 3.945764723 | 0.000429911 | 0.006215049 | -0.067134345 |
| SNCA | 1.543452488 | -0.620100738 | 3.996478722 | 0.000453817 | 0.006359886 | -0.067423282 |
| TOMM40 | -1.298357726 | -0.108252667 | -3.910900295 | 0.000407694 | 0.006002141 | -0.074223521 |
| TRIM22 | 2.906761006 | -0.111818001 | 4.204797506 | 0.00054984 | 0.007149236 | -0.074291072 |
| OMG | -1.195274438 | -0.165122608 | -3.908382378 | 0.000410648 | 0.00603869 | -0.080965515 |
| PRORY | -1.04234954 | -0.043317145 | -3.911904026 | 0.000436474 | 0.006239581 | -0.084270093 |
| ARF3 | -1.103814776 | -0.402221039 | -3.906107796 | 0.000413334 | 0.006071226 | -0.087054602 |
| FZD2 | 1.744502076 | -0.448655202 | 4.00247919 | 0.000472349 | 0.006473733 | -0.090112061 |
| NR1I3 | -1.26568943 | 0.217815124 | -3.904392888 | 0.00041537 | 0.006082879 | -0.091644576 |
| GUCY1B1 | 1.03816784 | -0.390518692 | 3.919087524 | 0.000427737 | 0.006209244 | -0.098819895 |
| TUBA1B | 1.362668159 | -0.320787311 | 3.901692759 | 0.000433266 | 0.00623543 | -0.121204872 |
| IFI16 | 2.352967646 | -0.512580773 | 4.009065241 | 0.000492763 | 0.006658268 | -0.12134018 |
| CCL19 | 1.48122578 | -0.073255893 | 3.89096018 | 0.000431663 | 0.006226335 | -0.127571915 |
| PNPLA4 | -1.04598894 | -0.106998592 | -3.887709569 | 0.000435698 | 0.006235441 | -0.136259223 |
| ACP5 | -1.720523451 | 0.17918143 | -3.915384159 | 0.000487708 | 0.00661086 | -0.139368292 |
| EFHD1 | -1.179060977 | -0.032046515 | -3.890751925 | 0.000463224 | 0.006410521 | -0.139472802 |
| GIPC1 | -1.420333326 | -0.068721129 | -3.885025606 | 0.000439057 | 0.006255593 | -0.143430138 |
| PCDH9 | -1.216385221 | -0.197413136 | -3.887107279 | 0.000451575 | 0.006348806 | -0.143506 |
| METTL1 | -1.646122512 | 0.024588765 | -3.904978341 | 0.000501737 | 0.006701852 | -0.145555444 |
| ACOT7 | -1.73337113 | -0.292615178 | -3.895731044 | 0.000456787 | 0.006387038 | -0.160188856 |
| IGKC | 1.066441076 | -0.059674006 | 3.878733665 | 0.000447032 | 0.006320057 | -0.160233546 |
| HLA-DMA | 1.729046883 | -0.311159534 | 3.936276148 | 0.000482007 | 0.006554377 | -0.162287858 |
| POSTN | 1.584327935 | -0.386158719 | 3.958827166 | 0.000501094 | 0.006700241 | -0.169726679 |
| SMARCA5 | 1.120795208 | -0.604211929 | 3.873946615 | 0.000453194 | 0.006358097 | -0.173011199 |
| PEAK1 | -1.110096491 | -0.052828681 | -3.875229301 | 0.00046704 | 0.006432711 | -0.174856178 |
| SYT11 | 1.124005336 | -0.268568093 | 3.889902359 | 0.000464332 | 0.006416082 | -0.175484353 |
| CXCL9 | 1.427134859 | -0.323276582 | 3.88863278 | 0.000465991 | 0.006425164 | -0.178814918 |
| FHL2 | 1.056202762 | 0.108097628 | 3.998226331 | 0.000539335 | 0.007042663 | -0.181361538 |
| AXL | 1.332595398 | -0.481016871 | 3.870072952 | 0.00045824 | 0.006393917 | -0.183346537 |
| SLPI | 1.762171533 | -0.12414861 | 3.922145922 | 0.000500724 | 0.006700241 | -0.185646481 |
| CHRM5 | -1.09847633 | -0.196779181 | -3.864530541 | 0.000465555 | 0.006425164 | -0.198127565 |
| DGCR6L | -1.144374219 | -0.181965402 | -3.860702456 | 0.000486656 | 0.006603593 | -0.213151109 |
| MPC1 | -1.126679284 | 0.004250228 | -3.855774419 | 0.000477345 | 0.006504771 | -0.221463069 |
| TXNDC5 | 1.467623741 | -0.419740474 | 3.871405922 | 0.000489087 | 0.006622555 | -0.223970442 |
| HCLS1 | 2.244093377 | -0.581822973 | 3.877108399 | 0.000499563 | 0.006700241 | -0.232780958 |
| GBP2 | 1.99499684 | -0.465354885 | 3.919381576 | 0.000555822 | 0.007183221 | -0.256250474 |
| EVL | 1.52333729 | -0.384727829 | 3.898288321 | 0.000533955 | 0.006993771 | -0.257843401 |
| CSGALNACT1 | 1.451859841 | -0.414850276 | 3.857318079 | 0.000527794 | 0.006941439 | -0.268926981 |
| PTDSS2 | -1.094549726 | -0.202832692 | -3.831288993 | 0.000511874 | 0.006791404 | -0.286611887 |
| ABI1 | 1.108206927 | -0.539104783 | 3.829463655 | 0.000514543 | 0.006816106 | -0.291462281 |
| ZEB2 | 2.389183818 | -0.590078991 | 3.914804462 | 0.000592884 | 0.007490201 | -0.310788492 |
| DIP2C | -1.217656412 | 0.431868696 | -3.848234982 | 0.000610849 | 0.007604779 | -0.31684796 |
| DCAF6 | 1.075351657 | -0.422382002 | 3.818881222 | 0.000530287 | 0.006958777 | -0.319565109 |
| MYOG | -1.082244062 | -0.029012529 | -3.821244416 | 0.000562875 | 0.00721775 | -0.32015304 |
| ARPC5 | 1.015788482 | -0.269826383 | 3.901200514 | 0.000614124 | 0.007630724 | -0.343603691 |
| RSAD2 | 1.002320567 | -0.445892594 | 3.805515073 | 0.000550842 | 0.007149236 | -0.355017634 |
| PJA1 | 1.406733115 | -0.60657878 | 3.844654559 | 0.000591196 | 0.007476244 | -0.365965297 |
| SLC2A10 | 2.687145981 | -0.85970961 | 3.920807467 | 0.000655091 | 0.007917275 | -0.368614981 |
| SLC31A1 | -1.26337485 | 0.005206351 | -3.799674672 | 0.000578196 | 0.007370808 | -0.373456519 |
| HIKESHI | 1.341765896 | -0.333482957 | 3.793906493 | 0.00056932 | 0.007278558 | -0.38576927 |
| KLC1 | 1.343855564 | -0.447470576 | 3.835911777 | 0.000605393 | 0.00758101 | -0.388094462 |
| HAPLN2 | -1.749863114 | -0.224446407 | -3.786049835 | 0.000582167 | 0.007405874 | -0.406561115 |
| MAFB | 1.290708655 | -0.087933136 | 3.983035606 | 0.000749947 | 0.00858622 | -0.408222967 |
| MAP3K19 | -1.103705678 | -0.022958934 | -3.782636891 | 0.000606609 | 0.007585977 | -0.418041278 |
| C11ORF71 | -2.287990767 | -0.280261262 | -3.781399314 | 0.000608725 | 0.007593079 | -0.421276848 |
| MLXIPL | -1.262230633 | -0.124071433 | -3.780991549 | 0.000609424 | 0.007594408 | -0.422342839 |
| CASQ2 | -1.721611781 | -0.262355091 | -3.778403413 | 0.000613877 | 0.007630724 | -0.429107794 |
| GNL3 | 1.40054799 | -0.348329673 | 3.80030709 | 0.000618102 | 0.007657899 | -0.431374324 |
| SHISAL1 | -1.008615694 | -0.230689445 | -3.776255312 | 0.000598576 | 0.007539822 | -0.43245764 |
| PLA2G2D | -1.184253165 | -0.184627397 | -3.776454104 | 0.000617252 | 0.007654764 | -0.434201802 |
| FCHSD2 | 1.147250443 | -0.164048542 | 3.926380674 | 0.000736463 | 0.008500152 | -0.437201035 |
| CEP192 | 1.150772867 | -0.466294337 | 3.773475294 | 0.000622444 | 0.00767482 | -0.441984215 |
| RPA3 | -1.083804727 | -0.034379043 | -3.773853897 | 0.000642482 | 0.007825333 | -0.442682781 |
| PLSCR1 | 1.190999047 | -0.088901905 | 3.828482077 | 0.000673148 | 0.008037036 | -0.459932795 |
| IL10RA | 1.693118 | -0.154755336 | 3.782226306 | 0.000649767 | 0.007882649 | -0.462265904 |
| SLC22A7 | -1.089152607 | 0.136941089 | -3.76397861 | 0.000619779 | 0.00766386 | -0.464879618 |
| TUT7 | 1.242000769 | -0.593403201 | 3.770202504 | 0.000628198 | 0.00772328 | -0.467703291 |
| GLI3 | -1.27037197 | -0.200558741 | -3.76277085 | 0.000621904 | 0.00767482 | -0.468066974 |
| PAXIP1 | 1.103681948 | -0.266018441 | 3.768578615 | 0.000631072 | 0.00773709 | -0.47195476 |
| HLA-C | 2.133297204 | -0.782751849 | 3.771999227 | 0.00064581 | 0.007856946 | -0.483154305 |
| ACKR3 | -1.618705458 | 0.342708748 | -3.798298997 | 0.00072912 | 0.008461095 | -0.489527444 |
| RABGAP1L | 1.162801929 | -0.512083569 | 3.75904913 | 0.000648196 | 0.007878498 | -0.496889545 |
| GJB4 | -1.07575744 | 0.039642776 | -3.747669089 | 0.000669237 | 0.008012722 | -0.509306981 |
| TGFBR2 | 1.169907589 | -0.62378156 | 3.757903088 | 0.000671661 | 0.008026757 | -0.519709268 |
| SSTR4 | -1.055695933 | 0.025701794 | -3.741019662 | 0.000661403 | 0.007963541 | -0.525399845 |
| NECAP2 | 1.24274475 | -0.569412808 | 3.743797546 | 0.000676545 | 0.008046215 | -0.536746047 |
| TRIM5 | 1.754790704 | -0.767761128 | 3.750872553 | 0.000684928 | 0.00810224 | -0.537922283 |
| ARR3 | -1.332681726 | -0.127021653 | -3.733017459 | 0.000676538 | 0.008046215 | -0.54645887 |
| P2RY13 | 1.168117234 | -0.199057047 | 3.726331469 | 0.000689441 | 0.008133123 | -0.564040104 |
| NFE2L3 | 1.429589601 | -0.52661403 | 3.753002791 | 0.000757676 | 0.008643842 | -0.59715964 |
| RESF1 | 1.950173025 | -0.832131008 | 3.733800224 | 0.000742553 | 0.008539694 | -0.602229978 |
| DLEU1 | -1.130672244 | -0.009395473 | -3.711872369 | 0.000739836 | 0.008523737 | -0.602396074 |
| GOLGA3 | 1.117982537 | -0.474998706 | 3.711121753 | 0.000719688 | 0.008396862 | -0.603987267 |
| ITIH2 | -1.010078796 | -0.239300562 | -3.710815093 | 0.000720311 | 0.008396862 | -0.604792 |
| TIMP1 | 1.888830754 | -0.139796311 | 3.832832703 | 0.000866644 | 0.009401839 | -0.609318609 |
| SLC24A3 | 1.591740691 | -0.674332382 | 3.722482889 | 0.000741137 | 0.008531054 | -0.611337084 |
| C1S | 2.782525055 | -0.328709752 | 3.865736623 | 0.00091411 | 0.009793108 | -0.615158858 |
| ITGAV | 1.630023348 | -0.328917135 | 3.846410359 | 0.000893609 | 0.009637296 | -0.616695962 |
| KIN | 1.146371588 | -0.616132508 | 3.705315688 | 0.00073157 | 0.008481849 | -0.619218846 |
| CTSS | 2.253264206 | -0.573604988 | 3.69702682 | 0.000748863 | 0.008581478 | -0.640946808 |
| CTDSP1 | -1.34050908 | -0.220959285 | -3.696988436 | 0.000771271 | 0.008721349 | -0.640998114 |
| ALOX5 | 1.288531928 | -0.284595177 | 3.70300947 | 0.000758402 | 0.008644432 | -0.643024543 |
| MSL1 | 1.500843663 | -0.382046219 | 3.709270677 | 0.000768799 | 0.008701068 | -0.645431305 |
| HLA-J | 1.448039843 | -0.429248788 | 3.698303452 | 0.000768442 | 0.008701068 | -0.655257016 |
| DIPK2B | -1.011227322 | -0.197951726 | -3.689490261 | 0.00076493 | 0.008687951 | -0.660685283 |
| SLC25A15 | -1.318594996 | -0.077610228 | -3.688165038 | 0.000790514 | 0.008826729 | -0.663852788 |
| DDX6 | -1.349788234 | -0.024098891 | -3.687982853 | 0.000790916 | 0.008826729 | -0.664324461 |
| OPCML | -1.402980829 | -0.0459964 | -3.684908445 | 0.000822457 | 0.009076046 | -0.671127076 |
| PAQR5 | -1.312223079 | 0.064589473 | -3.691213813 | 0.000783813 | 0.00878569 | -0.673673561 |
| MALT1 | 1.333975323 | -0.407221813 | 3.682387377 | 0.000780377 | 0.008774451 | -0.679272675 |
| SLC26A2 | 2.038925674 | -0.572333501 | 3.711154178 | 0.000817933 | 0.009057307 | -0.680653635 |
| PC | -2.066456242 | -0.25076296 | -3.694182228 | 0.00088775 | 0.00959833 | -0.691677889 |
| CDC25B | 1.525769337 | -0.484581199 | 3.733380094 | 0.00086538 | 0.009396623 | -0.693840987 |
| CEBPA-DT | -1.388863533 | 0.004419396 | -3.674569211 | 0.000797729 | 0.008879585 | -0.699714642 |
| MYL10 | -1.31528804 | -0.117431471 | -3.673323621 | 0.000823934 | 0.009084494 | -0.702246145 |
| TUBAL3 | -1.508550671 | 0.224607959 | -3.6832694 | 0.000914166 | 0.009793108 | -0.739465111 |
| NR3C1 | 1.170524065 | -0.430765848 | 3.655097857 | 0.000842584 | 0.009218814 | -0.750546791 |
| ZDHHC13 | 1.049154388 | -0.221041842 | 3.660638844 | 0.00085358 | 0.009307361 | -0.752933917 |
| FGL2 | 1.711806684 | -0.438684287 | 3.703041699 | 0.000937305 | 0.009908538 | -0.755561222 |
| DNAJC10 | 1.298692183 | -0.51836849 | 3.662558971 | 0.000874864 | 0.009482997 | -0.765595172 |
| DSE | 2.142387085 | -0.765786107 | 3.656516091 | 0.000917904 | 0.009801321 | -0.799384481 |
| TMEM248 | 1.10954924 | -0.207640488 | 3.631675929 | 0.000899776 | 0.009695655 | -0.811540949 |
| HYAL1 | -1.631575556 | 0.257042309 | -3.625760697 | 0.000998338 | 0.010272673 | -0.818002929 |
| RNF219 | 1.805104819 | -0.743973337 | 3.641760202 | 0.000926534 | 0.009856359 | -0.818906473 |
| KLKB1 | -1.454459516 | 0.008535006 | -3.627697936 | 0.000935471 | 0.009905238 | -0.819880723 |
| PLCB4 | 1.056325384 | -0.259858329 | 3.628451914 | 0.000907937 | 0.009750814 | -0.819923672 |
| BTNL8 | -1.410099854 | -0.236410244 | -3.623976921 | 0.000945186 | 0.009965645 | -0.829447749 |
| HCP5 | 1.762261366 | -0.534773622 | 3.643255335 | 0.000951783 | 0.009995671 | -0.833056592 |
| ANXA1 | 1.929066709 | 0.274620623 | 3.801540265 | 0.00123375 | 0.011771113 | -0.841708541 |
| SERPINF2 | -1.126414075 | 0.107479752 | -3.618864537 | 0.000958692 | 0.010033912 | -0.842585438 |
| STC1 | -1.887182532 | -0.007460883 | -3.625325205 | 0.000969442 | 0.010098418 | -0.843492491 |
| MXI1 | -1.370355383 | -0.044366866 | -3.617682896 | 0.000961841 | 0.0100519 | -0.845620896 |
| FOLR3 | -1.254382626 | 0.062323539 | -3.614123624 | 0.000971384 | 0.010102238 | -0.854761628 |
| TPBG | 2.657063295 | -0.626814967 | 3.737842001 | 0.001161237 | 0.011366827 | -0.855612392 |
| CALM2 | 1.307427957 | -0.827511383 | 3.61355086 | 0.000946593 | 0.009965645 | -0.858626178 |
| AGPAT3 | -1.117762153 | 0.150180766 | -3.605309432 | 0.00096865 | 0.010098375 | -0.88000212 |
| IRF9 | 1.279536242 | -0.396516671 | 3.624690801 | 0.001001256 | 0.010272673 | -0.880116907 |
| LAPTM5 | 2.423804757 | -0.588432383 | 3.645800507 | 0.001048213 | 0.010591988 | -0.885945575 |
| CD80 | -1.029278321 | -0.039658432 | -3.601491831 | 0.000979035 | 0.010148358 | -0.889896732 |
| EML1 | 1.32823869 | -0.566522158 | 3.603166752 | 0.001001342 | 0.010272673 | -0.901185704 |
| TM9SF1 | 1.016902894 | -0.333878055 | 3.609151806 | 0.001044575 | 0.010563549 | -0.902637872 |
| CES1P1 | -1.013862501 | -0.040600293 | -3.593008661 | 0.001002498 | 0.010275808 | -0.911867421 |
| GLIPR1 | 1.145673647 | -0.176306751 | 3.598837606 | 0.001013422 | 0.010326139 | -0.912313197 |
| DNASE1 | -1.143578609 | 0.124066434 | -3.591944177 | 0.001005481 | 0.010281156 | -0.91462275 |
| SLC39A2 | -1.219615556 | -0.304551661 | -3.590645747 | 0.00100913 | 0.010294408 | -0.917983145 |
| AKAP12 | 1.12234018 | -0.089296373 | 3.61794846 | 0.001128412 | 0.011163336 | -0.940335971 |
| PDLIM1 | 2.036335261 | -0.408119637 | 3.62199408 | 0.001116403 | 0.011088795 | -0.94446907 |
| FNTA | 1.126831011 | -0.486573324 | 3.578610364 | 0.00104357 | 0.010561716 | -0.94910592 |
| SRSF4 | 1.322734175 | -0.474038884 | 3.581279961 | 0.001063879 | 0.010697873 | -0.957384629 |
| SLC12A5 | -1.160323698 | -0.008797406 | -3.570467867 | 0.001096148 | 0.010949244 | -0.966567276 |
| ZNHIT6 | 2.058611106 | -1.13868347 | 3.580533889 | 0.001096376 | 0.010949244 | -0.975129447 |
| PLEKHA1 | 1.049335688 | -0.074945459 | 3.571651343 | 0.001092571 | 0.01094232 | -0.982061899 |
| ABAT | -1.089804672 | 0.09936569 | -3.576875016 | 0.001176228 | 0.011452372 | -0.982489509 |
| COX7B | -1.015353377 | -0.071284211 | -3.565258012 | 0.001083105 | 0.010876007 | -0.983580456 |
| C1ORF216 | 1.299061854 | -0.593763074 | 3.558174648 | 0.001165613 | 0.011378622 | -1.031908544 |
| C1RL | 1.619889723 | -0.39180842 | 3.570884976 | 0.001195336 | 0.01155071 | -1.033054354 |
| SLC39A4 | -1.442525499 | 0.199148518 | -3.541887374 | 0.001218688 | 0.011694976 | -1.033955392 |
| PEA15 | 1.285298402 | -0.461678472 | 3.602458655 | 0.001268836 | 0.011963442 | -1.03445746 |
| STIMATE | -1.150791553 | -0.015515537 | -3.54465228 | 0.001146967 | 0.011304813 | -1.036670237 |
| ISG20 | 1.43201884 | -0.508424805 | 3.547447199 | 0.001168034 | 0.011389888 | -1.043968521 |
| TPGS2 | 1.106182504 | -0.518484383 | 3.570539336 | 0.00123585 | 0.011782387 | -1.051783777 |
| PSMB9 | 2.072775056 | -0.100006251 | 3.676948385 | 0.001525118 | 0.0134502 | -1.063442939 |
| SP140L | 1.185215881 | -0.218888492 | 3.558649883 | 0.001275584 | 0.012018232 | -1.065735892 |
| BAZ1A | 1.099466168 | -0.203149047 | 3.532212687 | 0.001187262 | 0.011507375 | -1.06865364 |
| GYG1 | 1.814208351 | -0.715801216 | 3.54837826 | 0.001232095 | 0.011771113 | -1.072566003 |
| NR4A3 | -1.050072555 | 0.006960226 | -3.523349963 | 0.001281936 | 0.012047182 | -1.080549518 |
| ACTB | 1.112124726 | -0.340625432 | 3.526524153 | 0.001206139 | 0.011611355 | -1.083262543 |
| DYNC1H1 | 1.523969202 | -0.523008522 | 3.524183594 | 0.00121399 | 0.011678168 | -1.089270318 |
| IRF8 | 1.015984194 | 0.133797529 | 3.554596455 | 0.001289411 | 0.012091776 | -1.091117699 |
| MNS1 | 1.224764292 | -0.267580097 | 3.540505205 | 0.00125865 | 0.011914691 | -1.09232669 |
| TUBB | 1.119338804 | -0.201839979 | 3.552323149 | 0.00129723 | 0.012115349 | -1.096720954 |
| GNG4 | -1.102985516 | 0.053303179 | -3.519548981 | 0.001229682 | 0.011758544 | -1.101161168 |
| CKLF | 1.275440927 | -0.268790829 | 3.53975119 | 0.001299593 | 0.0121271 | -1.110570014 |
| CKAP4 | 2.140736682 | -0.924421208 | 3.534590705 | 0.001359811 | 0.012467069 | -1.124850362 |
| BTN3A2 | 1.073755391 | -0.35216685 | 3.509668104 | 0.00126379 | 0.011933418 | -1.126488433 |
| PALLD | 1.512749138 | -0.619990193 | 3.562861114 | 0.001405157 | 0.012778956 | -1.129074487 |
| ZBTB38 | 1.239997352 | -0.531822424 | 3.517912863 | 0.001337962 | 0.012342938 | -1.131555839 |
| CYP3A7 | -1.631723832 | 0.223687228 | -3.501342989 | 0.00136114 | 0.012467069 | -1.13572943 |
| VCL | 1.140472379 | -0.526549559 | 3.504380626 | 0.001282415 | 0.012047182 | -1.14002829 |
| APOBEC3B | 1.11210063 | -0.0557803 | 3.514174726 | 0.001314402 | 0.012195787 | -1.143203569 |
| MSN | 1.3317077 | -0.417430266 | 3.50843031 | 0.001300379 | 0.0121271 | -1.143374219 |
| CD163 | 1.199620665 | -0.066984313 | 3.508116316 | 0.001301501 | 0.012128739 | -1.144172233 |
| TMSB10 | 1.140815727 | -0.100907478 | 3.573569359 | 0.001486625 | 0.013265823 | -1.148173404 |
| SLC27A3 | 1.110380907 | -0.5253074 | 3.500809515 | 0.001295143 | 0.012113505 | -1.149167701 |
| NXN | 1.921084627 | -0.299124954 | 3.610962531 | 0.001669326 | 0.014242028 | -1.171924939 |
| MYOM2 | -1.623448268 | -0.306400185 | -3.487750291 | 0.001342732 | 0.012368912 | -1.182553267 |
| SMARCE1 | 1.141715073 | -0.421873966 | 3.492312533 | 0.00135917 | 0.012467069 | -1.184296347 |
| GPNMB | 1.084652859 | -0.014126766 | 3.617780971 | 0.001748675 | 0.014617514 | -1.189446289 |
| FOSB | -1.961518637 | 0.805875798 | -3.542788452 | 0.001762184 | 0.014625556 | -1.194810412 |
| PNP | -1.466537323 | 0.17014797 | -3.496570292 | 0.001504003 | 0.013332085 | -1.198693536 |
| HIGD1B | -1.254370983 | -0.047240481 | -3.473717271 | 0.001395743 | 0.012711374 | -1.218364143 |
| NCK2 | 1.583200145 | -0.858082792 | 3.482858115 | 0.001431296 | 0.012915909 | -1.222054307 |
| TNK1 | -1.087675156 | -0.149666553 | -3.464842072 | 0.001465319 | 0.013121357 | -1.234633514 |
| CDHR5 | -1.580148837 | -0.023011048 | -3.466960542 | 0.001534944 | 0.01350898 | -1.237218115 |
| GLI1 | -1.59536039 | -0.244683198 | -3.465000921 | 0.001429678 | 0.012911513 | -1.24057367 |
| EBP | -1.099547898 | 0.05668613 | -3.463397038 | 0.001436008 | 0.01294312 | -1.244657581 |
| FZD7 | 2.096807541 | -0.626362376 | 3.513349493 | 0.001595775 | 0.013882372 | -1.246899717 |
| SETD3 | -1.085075526 | -0.205114173 | -3.459311403 | 0.001452255 | 0.013049938 | -1.255056705 |
| CD74 | 1.53913894 | -0.418443885 | 3.467017704 | 0.001494177 | 0.01330544 | -1.261820817 |
| SOX15 | -1.341613374 | -0.120716395 | -3.44972785 | 0.001565849 | 0.01370572 | -1.264560337 |
| SLC22A18 | -1.770163381 | -0.053008236 | -3.468341705 | 0.001620519 | 0.01399321 | -1.267342426 |
| MPHOSPH8 | 2.268335023 | -0.774639649 | 3.487080944 | 0.001592258 | 0.013874276 | -1.273542988 |
| RHOA | 1.851894672 | -0.624272044 | 3.500322467 | 0.001649967 | 0.01414273 | -1.277808891 |
| LY86 | 1.519706873 | -0.351026303 | 3.453424693 | 0.001511745 | 0.013366043 | -1.282680868 |
| IFIT5 | 1.15700057 | -0.324591835 | 3.458010987 | 0.001531109 | 0.013484486 | -1.284396071 |
| DCK | 2.020194248 | -0.606980002 | 3.502150011 | 0.001706577 | 0.014415969 | -1.293077352 |
| BIRC3 | 1.35053372 | -0.278445049 | 3.469985137 | 0.001613503 | 0.013960829 | -1.298734938 |
| PCLAF | 1.048861862 | -0.201178926 | 3.439195362 | 0.001571589 | 0.01373721 | -1.318555002 |
| RAB5C | -1.361095103 | -0.08149462 | -3.433063391 | 0.001560923 | 0.013680498 | -1.321727952 |
| IFI44L | 1.550290337 | -0.400452211 | 3.452220128 | 0.001642027 | 0.014121873 | -1.327036508 |
| KNG1 | -1.286059851 | 0.349773289 | -3.409296239 | 0.001893099 | 0.015403048 | -1.327695137 |
| FABP4 | 1.314705474 | -0.288883962 | 3.444350794 | 0.001631055 | 0.014055795 | -1.332180998 |
| HIST1H2BD | -1.482747161 | -0.134111102 | -3.427449382 | 0.001585159 | 0.01383696 | -1.335956671 |
| FUT6 | -1.066569255 | -0.019498091 | -3.426994111 | 0.00158714 | 0.013844829 | -1.337110072 |
| DLST | -1.730352588 | -0.077218539 | -3.413117115 | 0.001773412 | 0.014695189 | -1.344631819 |
| DEFB1 | -1.788613523 | -0.257180241 | -3.403669296 | 0.001867956 | 0.01531509 | -1.354963245 |
| MAPK8IP2 | -1.578509967 | -0.182059738 | -3.418450869 | 0.001624761 | 0.014020399 | -1.358740331 |
| ADRB2 | 1.042810887 | -0.060231759 | 3.420672667 | 0.001693806 | 0.014383892 | -1.358940468 |
| ABCG2 | -1.386355557 | -0.004240327 | -3.414678537 | 0.00168008 | 0.014295729 | -1.360671532 |
| CDKN1B | 1.740503823 | -0.521533936 | 3.486035399 | 0.001931916 | 0.015638783 | -1.363787215 |
| SRPX | 1.147753159 | -0.087409847 | 3.429094999 | 0.001699155 | 0.014404197 | -1.369976251 |
| CDC7 | 1.372568368 | -0.311138381 | 3.417753135 | 0.00166609 | 0.014236378 | -1.37248477 |
| NECAB2 | -1.09181945 | -0.00916684 | -3.409804106 | 0.001702491 | 0.014404197 | -1.372873497 |
| FNBP4 | 1.259527416 | -0.536318527 | 3.41876242 | 0.001702562 | 0.014404197 | -1.382466134 |
| ARHGEF6 | 1.411336936 | -0.458402889 | 3.40825009 | 0.001670803 | 0.014245151 | -1.384533471 |
| IL7R | 1.339260577 | -0.219511344 | 3.415868571 | 0.001760406 | 0.014625556 | -1.384679755 |
| RAPGEF6 | 1.104172256 | -0.561399605 | 3.41287768 | 0.001688326 | 0.014346859 | -1.384725273 |
| A2M | 1.326018593 | -0.046233709 | 3.4580706 | 0.001984366 | 0.015943311 | -1.405382615 |
| ZNF432 | 1.223764453 | -0.451848062 | 3.410853281 | 0.001832732 | 0.015074482 | -1.41137323 |
| EMC6 | -1.275963155 | -0.375707151 | -3.397514268 | 0.001720611 | 0.0144868 | -1.411639556 |
| ALAD | -1.683244498 | -0.043478503 | -3.405539539 | 0.001764377 | 0.014634284 | -1.415392289 |
| CPLX3 | -1.654904642 | -0.288167323 | -3.393949902 | 0.00173746 | 0.014561736 | -1.420629856 |
| LHFPL6 | 1.435998336 | -0.042766242 | 3.508465277 | 0.002250167 | 0.01739234 | -1.421419818 |
| ARHGEF40 | 1.412960028 | -0.517592456 | 3.438799735 | 0.001931 | 0.015638783 | -1.423245392 |
| GAB2 | 1.473982909 | -0.635662466 | 3.424029023 | 0.001877155 | 0.015331345 | -1.425728819 |
| TERF2IP | 1.018044396 | -0.590259646 | 3.389281354 | 0.001759769 | 0.014625556 | -1.432398313 |
| CHN1 | 1.459013764 | -0.435572033 | 3.457134706 | 0.002169231 | 0.016950672 | -1.458916489 |
| SPX | -1.197688918 | -0.105678392 | -3.371643157 | 0.001888141 | 0.015372514 | -1.468117469 |
| HSP90B1 | 1.091698675 | -0.39554532 | 3.374598006 | 0.001873099 | 0.015325186 | -1.480546332 |
| RBP4 | -1.88639932 | 0.178438407 | -3.362174647 | 0.002084527 | 0.016459288 | -1.48362955 |
| TAX1BP3 | 1.436949662 | -0.371591577 | 3.385611325 | 0.001959401 | 0.015792208 | -1.490242804 |
| FCER1A | 1.598729322 | -0.107771531 | 3.37211784 | 0.001930383 | 0.015638783 | -1.49835466 |
| SLC49A3 | -1.305655271 | -0.215469125 | -3.356962068 | 0.001921908 | 0.015588001 | -1.513652427 |
| MCUB | 1.528748621 | -0.16909078 | 3.430045268 | 0.002316129 | 0.01773797 | -1.519351257 |
| NR0B2 | -1.381185228 | -0.109992473 | -3.349862811 | 0.00200265 | 0.01603996 | -1.522249912 |
| PPT1 | 1.228979367 | -0.523353484 | 3.350433047 | 0.001999569 | 0.016035314 | -1.540771158 |
| VEGFC | 1.150919472 | -0.508727489 | 3.344297644 | 0.002032959 | 0.016205941 | -1.556028969 |
| ZNF428 | -1.437261337 | -0.41087472 | -3.336481625 | 0.00203196 | 0.016205941 | -1.564944498 |
| SPAG5 | -2.594485206 | -0.171796535 | -3.330782482 | 0.002324765 | 0.017745741 | -1.574017725 |
| XPOT | 1.653799588 | -0.71904882 | 3.329965885 | 0.00206822 | 0.016380964 | -1.581230192 |
| ALLC | -1.043872779 | -0.015754361 | -3.327340198 | 0.002083006 | 0.016459288 | -1.587788463 |
| RAI14 | 1.944651362 | -1.148646914 | 3.361062341 | 0.002278577 | 0.017555047 | -1.59081252 |
| PGPEP1 | -1.060722002 | -0.038946568 | -3.321879378 | 0.002159565 | 0.016902517 | -1.591551495 |
| GIMAP6 | 1.053863423 | -0.200266422 | 3.376509442 | 0.002430057 | 0.018222585 | -1.592027954 |
| DDX60 | 1.456165554 | -0.373443094 | 3.39503116 | 0.002520449 | 0.018606629 | -1.597267247 |
| B2M | 2.250174796 | -0.59299449 | 3.384031609 | 0.002481259 | 0.01847688 | -1.602972044 |
| IFT52 | 1.351259789 | -0.655194491 | 3.319766511 | 0.002126224 | 0.016737 | -1.606691071 |
| MX1 | 1.392217069 | -0.299447943 | 3.359704267 | 0.002361118 | 0.017851777 | -1.608853435 |
| RTP4 | 1.408408971 | -0.364799383 | 3.365498741 | 0.002407802 | 0.018140571 | -1.611004718 |
| CAPN6 | 1.195489595 | -0.226646194 | 3.317988052 | 0.002136496 | 0.016797237 | -1.611126677 |
| ANXA5 | 1.123440893 | -0.273612069 | 3.364704375 | 0.0024126 | 0.018159129 | -1.612839975 |
| BDKRB1 | -1.31294935 | -0.040698709 | -3.312987017 | 0.002165637 | 0.016932905 | -1.623593246 |
| RIPOR2 | 1.573591407 | -0.565657471 | 3.316711753 | 0.002349503 | 0.017776074 | -1.639870923 |
| TSPYL2 | -1.250817862 | -0.270740118 | -3.311268562 | 0.002325466 | 0.017745741 | -1.640718654 |
| PPP1R3C | 1.62282899 | -0.572421684 | 3.328320208 | 0.002405758 | 0.018140571 | -1.654612797 |
| SSB | 1.08731188 | -0.62882515 | 3.294243666 | 0.00227827 | 0.017555047 | -1.670232481 |
| MCM6 | 1.337445734 | -0.124202639 | 3.378324566 | 0.002880525 | 0.020295038 | -1.67430699 |
| ZNF574 | -1.263810902 | -0.253264783 | -3.282951426 | 0.002449856 | 0.018317496 | -1.674867259 |
| APOM | -1.421243119 | 0.32205043 | -3.276775591 | 0.002747137 | 0.019635755 | -1.687781977 |
| VNN2 | 1.100628369 | -0.29388277 | 3.292619242 | 0.002567386 | 0.018833862 | -1.689517318 |
| HMG20A | 1.306206334 | -0.248477116 | 3.286098532 | 0.002429435 | 0.018222585 | -1.690523703 |
| CEP170 | 1.88746873 | -0.654433824 | 3.312326574 | 0.002507058 | 0.018571355 | -1.692599168 |
| SIRPB1 | -1.055862017 | -0.116550873 | -3.285171695 | 0.002334776 | 0.017745741 | -1.692758313 |
| FMO5 | -1.391689016 | 0.191123452 | -3.267765229 | 0.002608522 | 0.019023383 | -1.696617761 |
| HDGF | -1.003794198 | 0.019786362 | -3.282918261 | 0.002349018 | 0.017776074 | -1.698348735 |
| GMDS | 2.251510057 | -0.879681464 | 3.329028544 | 0.002732778 | 0.019554916 | -1.710491712 |
| XYLT1 | 1.480632369 | -0.545016601 | 3.298857031 | 0.002526131 | 0.018627129 | -1.712366727 |
| APMAP | 1.217568956 | -0.443383861 | 3.289317374 | 0.002464399 | 0.018372669 | -1.712746825 |
| TM4SF5 | -1.38078603 | 0.084690867 | -3.265840164 | 0.002684323 | 0.019360716 | -1.714169265 |
| PLOD2 | 1.255516947 | -0.434583228 | 3.278574904 | 0.002425811 | 0.018212045 | -1.718613751 |
| LILRB1 | -1.048183984 | -0.155260415 | -3.27205798 | 0.002418827 | 0.01817025 | -1.725264016 |
| ENO3 | -1.19747816 | -0.25018298 | -3.264619375 | 0.002518104 | 0.018600029 | -1.732468148 |
| DDX58 | 1.000565077 | -0.268831395 | 3.268354608 | 0.002443082 | 0.01828817 | -1.734431759 |
| TRAPPC6A | -1.092373016 | 0.007055703 | -3.256443226 | 0.002522677 | 0.018612366 | -1.763882441 |
| HSPB8 | 1.25481197 | -0.454831013 | 3.273998521 | 0.002766795 | 0.019722394 | -1.76732586 |
| MELK | 1.101031916 | -0.136780642 | 3.26659216 | 0.002616594 | 0.019023383 | -1.767849784 |
| FKBP9 | 1.20283108 | -0.62258414 | 3.26161729 | 0.002592685 | 0.018951536 | -1.76989428 |
| NIBAN1 | 1.217643292 | -0.266701187 | 3.288825516 | 0.002824156 | 0.020007821 | -1.773784837 |
| EIF3M | 1.406313281 | -0.866780253 | 3.254205779 | 0.002589149 | 0.018939336 | -1.778491178 |
| VOPP1 | 1.930138554 | -0.711539715 | 3.26855508 | 0.002732685 | 0.019554916 | -1.784666708 |
| RAB3IL1 | -1.121483368 | -0.070455851 | -3.241403897 | 0.002679112 | 0.019355043 | -1.789253218 |
| APOL1 | 1.22976401 | -0.392634868 | 3.26527961 | 0.002755959 | 0.019687834 | -1.792464064 |
| SNRNP25 | -1.215616569 | -0.063972643 | -3.237915307 | 0.00270414 | 0.019425231 | -1.797768646 |
| CD69 | 1.927059778 | -0.435885772 | 3.257957433 | 0.002740135 | 0.019596623 | -1.799087842 |
| GPR137B | -1.269294135 | 0.200536174 | -3.232885883 | 0.002996611 | 0.020874665 | -1.805266118 |
| DNMT3L | -4.222104194 | -0.288919709 | -3.192626064 | 0.003594546 | 0.023540788 | -1.805267343 |
| MTX1 | -1.391739645 | -0.251280115 | -3.234576852 | 0.002728299 | 0.019554916 | -1.805913256 |
| PIEZO1 | 1.619432613 | -0.725445943 | 3.254011694 | 0.002768487 | 0.019722394 | -1.808547736 |
| NUP155 | 1.046517329 | -0.256928878 | 3.238437337 | 0.00270038 | 0.019420626 | -1.817115952 |
| MPZ | -1.095574272 | -0.206497302 | -3.23119268 | 0.002699668 | 0.019420626 | -1.826130406 |
| SERPINB9 | 1.351591516 | -0.410906253 | 3.265217675 | 0.002996979 | 0.020874665 | -1.828411267 |
| CLU | 1.111022046 | 0.165483322 | 3.264675179 | 0.003001068 | 0.020891796 | -1.829664696 |
| MCUR1 | -1.036476612 | 0.08365249 | -3.221664722 | 0.003084574 | 0.021244726 | -1.831575321 |
| SUGCT | -1.146440136 | 0.268247441 | -3.221623585 | 0.003084901 | 0.021244726 | -1.831671694 |
| ABCC1 | 1.136620404 | -0.489955389 | 3.255576656 | 0.002981839 | 0.020836945 | -1.838456002 |
| HMGCL | -1.075481517 | -0.175357737 | -3.226543466 | 0.002787282 | 0.01979032 | -1.846186969 |
| CCL20 | 1.142000776 | -0.076390932 | 3.225070184 | 0.00279823 | 0.019846078 | -1.849784173 |
| RPA1 | 1.500749708 | -0.593379947 | 3.220143622 | 0.002780783 | 0.019755115 | -1.853289474 |
| VIM | 1.742326898 | 0.462354663 | 3.348549351 | 0.004168114 | 0.025970711 | -1.853610376 |
| TSPAN13 | 1.131908063 | -0.211316092 | 3.22182452 | 0.002943132 | 0.020645395 | -1.875864191 |
| STAT1 | 2.057906032 | -0.915110021 | 3.215822735 | 0.002926625 | 0.020574622 | -1.881135432 |
| SERPINA3 | 1.006356231 | 0.638308852 | 3.234385005 | 0.003238084 | 0.022001535 | -1.885714147 |
| P4HA2 | -1.651742175 | -0.168036649 | -3.203143459 | 0.003090609 | 0.021249801 | -1.901463374 |
| ZNF22 | 1.039803086 | -0.301219445 | 3.199982593 | 0.002934812 | 0.020620872 | -1.9027205 |
| ASGR2 | -1.08076524 | -0.106276107 | -3.193319924 | 0.003105395 | 0.021317177 | -1.915375568 |
| PPP1R2C | -1.126963946 | -0.202573238 | -3.194406289 | 0.002978828 | 0.020827431 | -1.916363673 |
| YBX2 | -1.220818281 | -0.12573156 | -3.193356318 | 0.002987186 | 0.020851759 | -1.918931159 |
| SLC7A5 | -1.09262358 | 0.250316245 | -3.19161583 | 0.00333247 | 0.022363092 | -1.927822526 |
| STC2 | -1.408755861 | -0.257505182 | -3.185633335 | 0.00304935 | 0.021111552 | -1.937802386 |
| MYL12A | 2.328345904 | -1.312572893 | 3.193289674 | 0.003318174 | 0.022363092 | -1.962935457 |
| FN1 | 1.260948681 | -0.212683096 | 3.183662294 | 0.003323685 | 0.022363092 | -1.976298723 |
| SLC9A3R1 | -1.604370749 | -0.268218171 | -3.166065641 | 0.003478362 | 0.023044495 | -1.976745809 |
| EFCC1 | -1.269929887 | -0.188750081 | -3.166200762 | 0.003211258 | 0.021868277 | -1.985178581 |
| FAM50A | 1.261432571 | -0.414267888 | 3.172339748 | 0.003281411 | 0.022181092 | -1.986029924 |
| COL4A2 | 1.377134871 | -0.408727706 | 3.16007585 | 0.003263961 | 0.022121447 | -2.000078866 |
| CDK6 | -1.427133931 | -0.214687305 | -3.159000344 | 0.0032733 | 0.022163793 | -2.002693688 |
| RHOQ | 1.195135075 | -0.602271093 | 3.158560912 | 0.003277123 | 0.022163793 | -2.003761919 |
| ELF1 | 1.386703098 | -0.713422979 | 3.159022521 | 0.003333406 | 0.022363092 | -2.010174908 |
| IFNGR1 | 1.613490879 | -0.645646463 | 3.168852831 | 0.003453413 | 0.022920864 | -2.011388058 |
| NDN | 1.077460233 | -0.216864025 | 3.218059042 | 0.004179781 | 0.026030761 | -2.015647987 |
| SOD3 | -1.407467109 | -0.067804304 | -3.147573774 | 0.003435564 | 0.022826028 | -2.016640254 |
| NAP1L1 | 1.520431471 | -0.573193276 | 3.155837139 | 0.003361535 | 0.022485311 | -2.01786653 |
| IGLL3P | 1.052262012 | 0.187661355 | 3.164206781 | 0.003495097 | 0.023071738 | -2.02237993 |
| ZEB1 | 1.010860899 | -0.517119065 | 3.150621526 | 0.00334693 | 0.022434751 | -2.023048298 |
| UBA1 | 1.632371499 | -0.534857156 | 3.144029638 | 0.003681764 | 0.023940801 | -2.028504272 |
| RABL2B | 1.202552262 | -0.308409324 | 3.145696645 | 0.003518658 | 0.023150208 | -2.049940664 |
| LCN2 | 1.041483178 | -0.044822929 | 3.139919777 | 0.003720934 | 0.024085582 | -2.060986882 |
| RUBCNL | 1.155566575 | -0.324550785 | 3.132643239 | 0.003510259 | 0.023130548 | -2.066624298 |
| FANCL | 1.743208924 | -0.842439694 | 3.161029621 | 0.003889005 | 0.024737731 | -2.067549693 |
| SQOR | 1.567465954 | -0.906031867 | 3.148724012 | 0.003904796 | 0.024799996 | -2.070393457 |
| SOCS5 | 1.174020838 | -0.69109854 | 3.130766739 | 0.003527736 | 0.023186138 | -2.071164793 |
| NIPSNAP1 | -1.2321569 | -0.084484325 | -3.124221148 | 0.003653241 | 0.023829113 | -2.072688958 |
| VCAM1 | 1.499264164 | -0.30673624 | 3.14246095 | 0.003779545 | 0.024317667 | -2.082225427 |
| CFH | 1.636815662 | 0.012088054 | 3.179767477 | 0.00438359 | 0.026972543 | -2.083435193 |
| KCNN2 | -1.42377247 | 0.366263187 | -3.116206475 | 0.003730922 | 0.024113025 | -2.091873781 |
| CLC | 1.11479293 | -0.13375151 | 3.115341146 | 0.003963606 | 0.025098925 | -2.095621397 |
| TMEM246 | 1.083962802 | -0.43521728 | 3.134835635 | 0.003853837 | 0.024573721 | -2.10004239 |
| RIDA | -1.492899272 | 0.468681042 | -3.137465923 | 0.004665805 | 0.028062675 | -2.100336456 |
| UBE2E1 | 1.463046642 | -0.744728126 | 3.129742245 | 0.003904235 | 0.024799996 | -2.111931738 |
| REG1A | 1.291083085 | -0.018718556 | 3.110257325 | 0.003724215 | 0.024094658 | -2.12069391 |
| AMMECR1 | 1.09977864 | -0.401899848 | 3.111722884 | 0.00377506 | 0.024300999 | -2.123963811 |
| GRINA | -1.112746319 | -0.004654432 | -3.105702813 | 0.003835103 | 0.024483327 | -2.138380488 |
| CHST8 | -1.330041901 | -0.176882411 | -3.093380346 | 0.003960843 | 0.025093821 | -2.146368026 |
| PHF21A | 1.428485565 | -0.731685757 | 3.082112169 | 0.004152522 | 0.025936576 | -2.180556073 |
| MYC | 1.169234821 | -0.060616977 | 3.089944607 | 0.004146612 | 0.025912284 | -2.189743943 |
| GLB1L2 | -1.122695382 | 0.239099778 | -3.054315535 | 0.004831135 | 0.02880039 | -2.197201464 |
| SGCE | 1.190173794 | -0.368481671 | 3.121344583 | 0.004847232 | 0.028829338 | -2.197506393 |
| SETD5 | 1.503213693 | -0.611170295 | 3.076300351 | 0.00407251 | 0.025609177 | -2.202304344 |
| SERPINA4 | -1.073695693 | 0.031027046 | -3.075961233 | 0.004076139 | 0.025609177 | -2.203116861 |
| PROM1 | 1.999392287 | 0.078562919 | 3.113311059 | 0.004778382 | 0.028540532 | -2.204655625 |
| CSTA | 1.579184802 | -0.222843762 | 3.100616058 | 0.00451765 | 0.027467912 | -2.204686654 |
| SEPTIN7 | 1.098515152 | -0.596490028 | 3.074332406 | 0.004237135 | 0.026324024 | -2.219737163 |
| DOCK4 | 1.451345998 | -0.476227181 | 3.089644931 | 0.004905092 | 0.029079053 | -2.235655035 |
| COL6A3 | 1.030378255 | 0.275318886 | 3.076142111 | 0.004681624 | 0.028105088 | -2.251819385 |
| MED17 | 1.384904506 | -0.461797792 | 3.065396397 | 0.004503927 | 0.027410465 | -2.254245379 |
| TUBA1A | 1.762967083 | -0.43160462 | 3.079584812 | 0.004886021 | 0.029006226 | -2.260594424 |
| SPG7 | 1.089916548 | -0.084557987 | 3.040975524 | 0.004540563 | 0.027538729 | -2.270652352 |
| RNASE1 | 1.107530888 | -0.377721879 | 3.047863968 | 0.004460017 | 0.027300496 | -2.27611712 |
| RAB27A | 1.502975142 | -0.519899991 | 3.047773605 | 0.004538476 | 0.027538729 | -2.282398345 |
| METRN | -1.243624562 | -0.061866035 | -3.041266059 | 0.004464045 | 0.02730185 | -2.285980634 |
| ARG2 | -1.129658281 | 0.169885469 | -3.03344847 | 0.004630149 | 0.027861287 | -2.288407754 |
| PJA2 | 1.047174518 | -0.301696803 | 3.029498104 | 0.004677832 | 0.028105088 | -2.29771643 |
| WNT10B | -1.025847404 | 0.055278675 | -2.999480696 | 0.00532476 | 0.030563741 | -2.300804094 |
| ARHGAP25 | 1.7207351 | -0.838083865 | 3.036931489 | 0.004588489 | 0.027727706 | -2.301991745 |
| CHST15 | 1.521291465 | -0.333278857 | 3.050346082 | 0.004879416 | 0.028980431 | -2.303075143 |
| AHCY | -1.674682477 | 0.099180136 | -3.030338727 | 0.005129842 | 0.029954444 | -2.309741505 |
| PLTP | 1.27906377 | -0.076607083 | 3.057276654 | 0.005303594 | 0.030490215 | -2.318440248 |
| CHP2 | -1.454613415 | -0.190706885 | -3.014076736 | 0.005037581 | 0.029525358 | -2.322330009 |
| PHF11 | 1.088726126 | -0.530885052 | 3.030729698 | 0.004742438 | 0.02839042 | -2.322455741 |
| MGAT4B | 1.049099207 | -0.311891994 | 3.01881906 | 0.004733332 | 0.028349144 | -2.339309189 |
| TAC1 | 2.280137656 | -0.044636457 | 3.034200295 | 0.005080575 | 0.029707346 | -2.339920075 |
| SPOUT1 | -1.174516538 | -0.127433734 | -3.003064569 | 0.005181079 | 0.03014081 | -2.347758621 |
| NUAK1 | 1.021783614 | -0.370458384 | 3.01968749 | 0.004879177 | 0.028980431 | -2.348342306 |
| TSC22D1 | 1.149482189 | -0.582639697 | 3.023169039 | 0.005014672 | 0.029429352 | -2.35218905 |
| NCL | 1.000194179 | -0.385340986 | 3.00336558 | 0.004927558 | 0.029160764 | -2.375892172 |
| SLC37A4 | -1.306814273 | -0.071240456 | -3.004328901 | 0.004992713 | 0.029340769 | -2.378846873 |
| LBH | 1.264860974 | -0.444668746 | 3.01675235 | 0.005306815 | 0.030490215 | -2.379627725 |
| AASS | -1.218059816 | -0.130268828 | -2.999169483 | 0.005232755 | 0.030339197 | -2.381437632 |
| FAM98A | 1.135835574 | -0.512178213 | 3.008476243 | 0.00530709 | 0.030490215 | -2.392162395 |
| BACE2 | -1.497084948 | 0.094717395 | -2.988438027 | 0.005286644 | 0.030484262 | -2.400029097 |
| MYO1B | 1.445235897 | -0.743618015 | 3.001131729 | 0.005405879 | 0.030853597 | -2.408954504 |
| TFPI2 | 1.63116924 | -0.037317452 | 3.00831519 | 0.005669133 | 0.0317201 | -2.411890607 |
| CRISPLD2 | 1.244367922 | -0.116489235 | 3.011029486 | 0.005772714 | 0.03211432 | -2.412730094 |
| TUSC3 | 1.736227779 | -0.412977104 | 3.023454949 | 0.006307696 | 0.034128094 | -2.415572762 |
| BTN2A1 | 1.560389917 | -0.760845144 | 2.995044171 | 0.00548908 | 0.03113068 | -2.422856718 |
| NXPH3 | -1.362061331 | -0.367341571 | -2.965358208 | 0.005608002 | 0.03151245 | -2.427814479 |
| PRDM1 | -1.472867522 | -0.292093396 | -2.980446781 | 0.005229504 | 0.030339197 | -2.429949382 |
| HAO1 | -2.275254325 | -0.312930248 | -2.975953929 | 0.005290705 | 0.03048557 | -2.440518407 |
| UBE2L6 | 1.30066221 | -0.350400216 | 2.990208082 | 0.005925695 | 0.032673476 | -2.452195816 |
| LIMK2 | 1.312117851 | -0.662330345 | 2.969589722 | 0.005460318 | 0.031036198 | -2.460224207 |
| TNFAIP6 | 1.085342569 | -0.183918159 | 2.967556225 | 0.00540691 | 0.030853597 | -2.460248472 |
| TRAFD1 | 1.011241139 | -0.505458303 | 2.966036121 | 0.0054282 | 0.030922172 | -2.463816436 |
| LPAR6 | 1.483747586 | -0.672163779 | 2.969506989 | 0.005548908 | 0.031338336 | -2.465324607 |
| FLII | 1.004583591 | -0.281222737 | 2.966218909 | 0.005507826 | 0.031181834 | -2.468091792 |
| ANXA3 | 1.904548784 | -0.254537315 | 2.980439529 | 0.006216145 | 0.033904565 | -2.480113368 |
| TFCP2L1 | -1.158550194 | 0.031384048 | -2.956316931 | 0.005649646 | 0.031663205 | -2.491173785 |
| FZD5 | -1.269635516 | -0.0104259 | -2.940634751 | 0.006175508 | 0.033769098 | -2.497504634 |
| KPNA2 | 1.40755492 | -0.520102137 | 2.955327774 | 0.005753334 | 0.032034305 | -2.49818153 |
| LMO2 | 1.158902092 | -0.520688763 | 2.9625744 | 0.006199773 | 0.033844055 | -2.507767182 |
| SCG5 | 1.070893769 | -0.245781668 | 2.938642792 | 0.005911371 | 0.032630868 | -2.509918918 |
| TBPL1 | 1.746395592 | -0.838052101 | 2.950405177 | 0.006257568 | 0.034029175 | -2.511932397 |
| ERAP2 | 1.206022541 | -0.329948405 | 2.943262071 | 0.005756767 | 0.032039504 | -2.517143678 |
| CLCNKA | -1.090991056 | 0.059882404 | -2.938811732 | 0.00582312 | 0.032279027 | -2.527536401 |
| TPPP3 | -2.183858291 | -0.747251521 | -2.964337174 | 0.007008248 | 0.036427548 | -2.533546138 |
| DKK3 | 1.389613145 | -0.513278324 | 2.941252597 | 0.006061009 | 0.033327634 | -2.535354409 |
| KCTD12 | 1.312602944 | -0.386240776 | 2.944138246 | 0.006233914 | 0.033972582 | -2.538533385 |
| IP6K2 | 2.726948261 | -1.458511918 | 2.937051648 | 0.006125667 | 0.033553794 | -2.54498376 |
| COL1A2 | 1.131969685 | 0.134156363 | 2.945832342 | 0.006459747 | 0.034571508 | -2.545097954 |
| PRRC2A | -1.046092807 | -0.116130702 | -2.895619359 | 0.006799155 | 0.03579046 | -2.56275386 |
| PLEKHO1 | 1.030004187 | -0.34287505 | 2.928070055 | 0.006266094 | 0.034061096 | -2.565545528 |
| SEMA6C | -1.682343813 | -0.230946899 | -2.921199958 | 0.006092829 | 0.033431042 | -2.568573761 |
| BET1 | 1.189853764 | -0.286574166 | 2.909573424 | 0.006366806 | 0.034265192 | -2.577031036 |
| ITM2C | 1.920913025 | -0.54218117 | 2.924596967 | 0.006669307 | 0.035266193 | -2.587423529 |
| ACPP | -1.956593132 | -0.001802593 | -2.911034024 | 0.006650521 | 0.03522496 | -2.588834893 |
| ENOSF1 | -1.28181779 | -0.171375646 | -2.908184984 | 0.006299637 | 0.034111545 | -2.598806334 |
| LEPROT | 1.466678101 | -0.727132363 | 2.91332229 | 0.006612605 | 0.035126193 | -2.603583291 |
| PIK3C2G | -1.682218781 | -0.171771218 | -2.893108103 | 0.0066392 | 0.035205263 | -2.61487181 |
| BNIP3L | 1.229965814 | -0.33702528 | 2.882299587 | 0.006923496 | 0.036207172 | -2.617826165 |
| YEATS2 | 1.504190302 | -0.85270466 | 2.901361331 | 0.006501323 | 0.034691306 | -2.618466736 |
| FZD6 | 1.065426756 | -0.146877639 | 2.899049072 | 0.006972546 | 0.036355209 | -2.621129511 |
| IDO1 | 1.062835438 | -0.268037029 | 2.898467243 | 0.006458331 | 0.034571508 | -2.621327423 |
| FMO4 | -1.299340286 | 0.246472064 | -2.874676281 | 0.007405453 | 0.037802599 | -2.622812194 |
| P4HA1 | -1.058553342 | 0.172508486 | -2.870961143 | 0.007473625 | 0.037954177 | -2.631030191 |
| AMIGO2 | 1.105232497 | -0.039327581 | 2.923979402 | 0.008481004 | 0.041382009 | -2.632659152 |
| C2ORF68 | 1.033216082 | -0.570243905 | 2.889064966 | 0.006615438 | 0.035126193 | -2.643074603 |
| ACAA1 | -1.196966025 | 0.066562787 | -2.862639376 | 0.007628479 | 0.038467355 | -2.649417156 |
| NUP37 | 1.838377458 | -0.939129255 | 2.882874828 | 0.006913461 | 0.036184207 | -2.664675589 |
| IGSF6 | 1.472846438 | -0.24558507 | 2.874324149 | 0.006868982 | 0.03602493 | -2.677084418 |
| MICAL2 | 1.921226011 | -0.855862944 | 2.878685542 | 0.007093996 | 0.036661236 | -2.677969318 |
| SLCO3A1 | 1.716928283 | -0.722399078 | 2.881981197 | 0.007273115 | 0.037305262 | -2.67834164 |
| NPR3 | -2.263578837 | -0.193650339 | -2.839904869 | 0.008524525 | 0.041515375 | -2.687302689 |
| IL10RB | 1.180337576 | -0.400355625 | 2.88442915 | 0.008012416 | 0.03986931 | -2.693690359 |
| EYA2 | -1.302173959 | -0.122540162 | -2.853162544 | 0.007346192 | 0.037574879 | -2.706148583 |
| ZKSCAN7 | 1.122317199 | -0.097373336 | 2.866784134 | 0.007425273 | 0.037858556 | -2.708552788 |
| MICALL2 | 1.094928407 | -0.280900875 | 2.858842345 | 0.007454783 | 0.037919417 | -2.722835956 |
| TNNI2 | -1.082956077 | -0.352215738 | -2.854377716 | 0.007226584 | 0.037096271 | -2.722937562 |
| ZNF280D | 1.321348223 | -0.418203843 | 2.851272372 | 0.007716412 | 0.038787752 | -2.723097648 |
| EGR1 | -1.338098579 | 0.221103524 | -2.865180667 | 0.008022734 | 0.039889692 | -2.727245859 |
| SNX5 | -1.339981483 | -0.306751228 | -2.851165433 | 0.007285779 | 0.037340344 | -2.73030393 |
| MAP7 | -1.010924085 | -0.169844246 | -2.848151034 | 0.007341739 | 0.037574879 | -2.737211935 |
| IGLC1 | 1.34128047 | -0.039170188 | 2.855574832 | 0.007762304 | 0.038942108 | -2.737245222 |
| MATN2 | 1.737053279 | -0.64504167 | 2.843687548 | 0.007992487 | 0.039785584 | -2.744412315 |
| CHODL | 1.287307305 | -0.556523763 | 2.847778835 | 0.007551366 | 0.038242958 | -2.744464748 |
| PRKX | 1.695366297 | -0.267027592 | 2.845442902 | 0.007708084 | 0.038761084 | -2.753031977 |
| ZCCHC24 | 1.038365523 | -0.436466761 | 2.842291094 | 0.007550482 | 0.038242958 | -2.753722765 |
| KHK | -1.068539131 | 0.119881657 | -2.814895148 | 0.008576901 | 0.041642544 | -2.754339161 |
| HEATR1 | 1.137786326 | -0.364986691 | 2.841119791 | 0.007572808 | 0.038311804 | -2.756387994 |
| CD2 | 1.173065739 | -0.204042894 | 2.835039711 | 0.007796271 | 0.039051398 | -2.773284632 |
| CBX1 | 1.467021911 | -0.805658053 | 2.837124386 | 0.007991281 | 0.039785584 | -2.77495673 |
| ST3GAL1 | -1.758039748 | -0.347845261 | -2.815502397 | 0.008429176 | 0.041237011 | -2.777606867 |
| CD81 | 1.639885083 | -0.43819697 | 2.839152156 | 0.008719267 | 0.042064392 | -2.787074114 |
| ARPC2 | 1.297993966 | -0.65286602 | 2.825056185 | 0.007784019 | 0.039035776 | -2.78998982 |
| INTS12 | 1.144934477 | -0.548216782 | 2.827913433 | 0.008772821 | 0.042227435 | -2.792460584 |
| NPRL2 | -1.05757044 | -0.180292555 | -2.823791909 | 0.007808936 | 0.039099565 | -2.792871439 |
| TNS3 | 2.025780226 | -1.26780399 | 2.832016906 | 0.008520564 | 0.041511853 | -2.795991584 |
| CYFIP1 | 1.142394988 | -0.621710131 | 2.821733468 | 0.008060012 | 0.040002121 | -2.8033059 |
| EOGT | 1.645442219 | -0.649653469 | 2.819729501 | 0.008100449 | 0.040105039 | -2.807819945 |
| ABHD6 | -1.178888623 | -0.092428153 | -2.817079058 | 0.007942488 | 0.039644422 | -2.808158548 |
| KCTD9 | 1.207549659 | -0.549866328 | 2.820179545 | 0.008611235 | 0.041704233 | -2.818758173 |
| MRC1 | 1.086699116 | 0.052180851 | 2.815334655 | 0.008567686 | 0.04161736 | -2.826428619 |
| CDK2AP1 | 1.027790262 | -0.499687694 | 2.807414988 | 0.008138505 | 0.040231321 | -2.830127126 |
| PTPRN2 | 1.176357241 | -0.605666542 | 2.80994308 | 0.008418759 | 0.041219455 | -2.832633457 |
| ZFC3H1 | 1.00501273 | -0.414603065 | 2.805892301 | 0.008273844 | 0.040712014 | -2.836236331 |
| ZNF573 | 2.109665012 | -1.217748778 | 2.807510329 | 0.008469643 | 0.041342305 | -2.838067051 |
| PNMA2 | 1.054062184 | -0.359309883 | 2.815205189 | 0.009430479 | 0.044311847 | -2.841108868 |
| SFXN3 | -1.05556727 | -0.282327477 | -2.798705346 | 0.008319024 | 0.040864168 | -2.849886164 |
| GCDH | -1.521063007 | -0.173331033 | -2.797789694 | 0.008338218 | 0.040903203 | -2.851961244 |
| ECHDC2 | -1.059764722 | -0.114206965 | -2.787686036 | 0.008659498 | 0.041854785 | -2.854116281 |
| SPHK2 | -1.092960647 | -0.096299081 | -2.784373592 | 0.008731444 | 0.042107298 | -2.861546499 |
| DENND5A | 1.00603762 | -0.431225312 | 2.792547451 | 0.008554911 | 0.041608202 | -2.866326506 |
| TLR3 | 1.109645253 | -0.308908646 | 2.790303622 | 0.008837847 | 0.042413068 | -2.876419453 |
| ARPC3 | 1.613221973 | -0.358106971 | 2.79406351 | 0.009335902 | 0.044084049 | -2.878493615 |
| FABP5 | 1.992202425 | -0.404251775 | 2.792092915 | 0.009555782 | 0.044674109 | -2.88526316 |
| PNMA8A | 1.501729461 | -0.960133225 | 2.786219586 | 0.008927414 | 0.042762881 | -2.885502012 |
| EIF3J | 1.245153705 | -0.633146828 | 2.78230504 | 0.008669128 | 0.041885542 | -2.886989236 |
| CPN2 | -1.171924707 | 0.233684515 | -2.766720322 | 0.009241576 | 0.043793321 | -2.904068786 |
| BANK1 | 1.053757879 | -0.524632214 | 2.767672189 | 0.009623049 | 0.044871404 | -2.911523161 |
| CDH6 | 1.075222334 | -0.076270345 | 2.766041781 | 0.009139798 | 0.043439446 | -2.925831247 |
| PLA2G4C | 1.690949236 | -0.589053228 | 2.765684011 | 0.010174681 | 0.046414065 | -2.925949708 |
| HADH | -1.2887771 | -0.156004112 | -2.744940968 | 0.009881582 | 0.045623588 | -2.928728457 |
| SCGB1D2 | -1.143611946 | 0.233021498 | -2.731657738 | 0.010348984 | 0.046850126 | -2.930293802 |
| FABP1 | -1.698996321 | 0.290381344 | -2.746243726 | 0.010295658 | 0.046766286 | -2.937610325 |
| SART3 | 1.289541133 | -0.663145932 | 2.759235017 | 0.009184912 | 0.043605377 | -2.938951216 |
| LTBP3 | 1.414026258 | -0.652952279 | 2.750227525 | 0.010038888 | 0.046056765 | -2.94950908 |
| LOC103344931 | 1.291128128 | -0.789335148 | 2.751918488 | 0.009354368 | 0.044084049 | -2.955374013 |
| GPR182 | -1.005487561 | -0.115983697 | -2.740552551 | 0.009737237 | 0.045269062 | -2.959330116 |
| MED4 | 1.289065779 | -0.573442807 | 2.749047887 | 0.0102265 | 0.046567619 | -2.973702278 |
| CXCR4 | 1.288727917 | -0.271822687 | 2.743551949 | 0.009915288 | 0.045702278 | -2.979916889 |
| TRAF5 | 1.343654896 | -0.26015176 | 2.748984127 | 0.011247076 | 0.049867863 | -2.98161426 |
| FAM13B | 1.316162492 | -0.776996331 | 2.73887769 | 0.010029503 | 0.046056193 | -2.990206962 |
| NRP1 | 1.187078691 | -0.267593567 | 2.734244028 | 0.009775798 | 0.045369185 | -2.994932947 |
| EIF3D | 1.193819842 | -0.238206005 | 2.726692912 | 0.010626575 | 0.047805741 | -3.00053555 |
| PFN1 | 1.136843645 | -0.107558902 | 2.732057921 | 0.010338921 | 0.046850126 | -3.00697224 |
| TM9SF2 | 1.263314671 | -0.495675571 | 2.73011324 | 0.01011539 | 0.046211995 | -3.007705432 |
| ASF1A | 1.119723738 | -0.57809031 | 2.726780684 | 0.010074972 | 0.046172675 | -3.013325886 |
| HLA-DMB | 1.160978749 | -0.409229929 | 2.723026924 | 0.010293193 | 0.046766286 | -3.023343031 |
| APOC1 | -1.458345028 | -0.069787698 | -2.720790201 | 0.010108352 | 0.046210123 | -3.024937037 |
| BCKDHA | -1.265872392 | -0.153493473 | -2.710631879 | 0.010484867 | 0.047323989 | -3.025541541 |
| TENT4A | 1.30105919 | -0.835748157 | 2.721041821 | 0.0102189 | 0.04654954 | -3.02604976 |
| ITGB5 | 1.357877548 | -0.871237378 | 2.721184741 | 0.01033989 | 0.046850126 | -3.02740413 |
| TMEM243 | 1.229467295 | -0.402755662 | 2.718835473 | 0.01053328 | 0.047525776 | -3.034208228 |
| HEXB | 1.492145983 | -0.737164319 | 2.7185212 | 0.01068445 | 0.048005149 | -3.036490207 |
| GRWD1 | -1.562052753 | -0.259952463 | -2.70541181 | 0.010620645 | 0.047803431 | -3.037046315 |
| UGDH | -1.000988381 | -0.195101371 | -2.709206871 | 0.010403058 | 0.047054115 | -3.050694165 |
| ARPC1B | 1.091569202 | -0.155300693 | 2.712685331 | 0.011337665 | 0.049956238 | -3.053331116 |
| OXR1 | 1.557956441 | -0.467479415 | 2.708108996 | 0.010812489 | 0.04846216 | -3.057676752 |
| CAVIN1 | 1.237496734 | -0.622575458 | 2.701003906 | 0.011147578 | 0.049496326 | -3.07455791 |
| UGCG | 1.289819682 | -0.300636833 | 2.688883381 | 0.011330242 | 0.049944207 | -3.07762632 |
| TUFT1 | -1.238764309 | -0.154215189 | -2.682901556 | 0.01110219 | 0.049397427 | -3.108926145 |
| FAM172A | -1.180957004 | -0.424462359 | -2.683453702 | 0.011340763 | 0.049956238 | -3.110206684 |
| LHX5 | -1.331096829 | -0.097177615 | -2.676709252 | 0.011272981 | 0.049867863 | -3.122580853 |
| ITGBL1 | 1.030488495 | -0.604102052 | 2.675377433 | 0.011310031 | 0.049922316 | -3.125514999 |
