# Supplementary material for: NET-Related Gene as Potential Diagnostic Biomarkers for Diabetic Tubulointerstitial Injury
Source: J Diabetes Res. 2024 May 10;2024:4815488. doi: 10.1155/2024/4815488 (PMC11101254; doi:10.1155/2024/4815488)
Supplement: Supporting Information — Additional supporting information can be found online in the Supporting Information section. Supporting Information S1: R language for DEGs. Table S2: DEGs identified in the gene expression microarray study. Table S3: GO enrichment analysis of DEG. Table S4: KEGG enrichment analysis of DEG. Table S5: DEG related to NETs identified through machine learning. [file 4815488.f1.zip › Supplementary table S3.docx]

| Supplementary table S3: GO enrichment analysis of DEG | | | | | | | | | |
| --- | --- | --- | --- | --- | --- | --- | --- | --- | --- |
| ONTOLOGY | ID | Description | GeneRatio | BgRatio | pvalue | p.adjust | qvalue | geneID | Count |
| BP | GO:0031349 | positive regulation of defense response | 8/17 | 441/18614 | 1.88E-09 | 1.78E-06 | 7.83E-07 | CASP1/MNDA/C3/LTF/TLR7/CCL5/CLEC7A/S100A8 | 8 |
| BP | GO:0030593 | neutrophil chemotaxis | 5/17 | 107/18614 | 3.35E-08 | 1.25E-05 | 5.51E-06 | CCL5/CCL2/CXCL1/S100A8/ITGB2 | 5 |
| BP | GO:0030595 | leukocyte chemotaxis | 6/17 | 241/18614 | 4.86E-08 | 1.25E-05 | 5.51E-06 | CCL5/CCL2/CXCL1/CXCR4/S100A8/ITGB2 | 6 |
| BP | GO:1990266 | neutrophil migration | 5/17 | 128/18614 | 8.23E-08 | 1.25E-05 | 5.51E-06 | CCL5/CCL2/CXCL1/S100A8/ITGB2 | 5 |
| BP | GO:0071216 | cellular response to biotic stimulus | 6/17 | 264/18614 | 8.35E-08 | 1.25E-05 | 5.51E-06 | CASP1/LTF/CCL5/CCL2/CXCL1/CLEC7A | 6 |
| BP | GO:0002831 | regulation of response to biotic stimulus | 7/17 | 463/18614 | 8.83E-08 | 1.25E-05 | 5.51E-06 | CASP1/MNDA/LTF/TLR7/CCL5/CLEC7A/FGL2 | 7 |
| BP | GO:0071621 | granulocyte chemotaxis | 5/17 | 131/18614 | 9.25E-08 | 1.25E-05 | 5.51E-06 | CCL5/CCL2/CXCL1/S100A8/ITGB2 | 5 |
| BP | GO:0045089 | positive regulation of innate immune response | 6/17 | 300/18614 | 1.78E-07 | 2.10E-05 | 9.26E-06 | CASP1/MNDA/LTF/TLR7/CCL5/CLEC7A | 6 |
| BP | GO:0097530 | granulocyte migration | 5/17 | 156/18614 | 2.21E-07 | 2.12E-05 | 9.33E-06 | CCL5/CCL2/CXCL1/S100A8/ITGB2 | 5 |
| BP | GO:0060326 | cell chemotaxis | 6/17 | 312/18614 | 2.24E-07 | 2.12E-05 | 9.33E-06 | CCL5/CCL2/CXCL1/CXCR4/S100A8/ITGB2 | 6 |
| BP | GO:0002833 | positive regulation of response to biotic stimulus | 6/17 | 327/18614 | 2.95E-07 | 2.54E-05 | 1.12E-05 | CASP1/MNDA/LTF/TLR7/CCL5/CLEC7A | 6 |
| BP | GO:0052548 | regulation of endopeptidase activity | 6/17 | 336/18614 | 3.46E-07 | 2.73E-05 | 1.20E-05 | CASP1/LTF/TIMP1/CLEC7A/S100A8/F3 | 6 |
| BP | GO:0032496 | response to lipopolysaccharide | 6/17 | 345/18614 | 4.04E-07 | 2.94E-05 | 1.30E-05 | CASP1/LTF/CCL5/CCL2/CXCL1/S100A8 | 6 |
| BP | GO:0002237 | response to molecule of bacterial origin | 6/17 | 366/18614 | 5.72E-07 | 3.86E-05 | 1.70E-05 | CASP1/LTF/CCL5/CCL2/CXCL1/S100A8 | 6 |
| BP | GO:0045088 | regulation of innate immune response | 6/17 | 373/18614 | 6.39E-07 | 4.03E-05 | 1.77E-05 | CASP1/MNDA/LTF/TLR7/CCL5/CLEC7A | 6 |
| BP | GO:0050900 | leukocyte migration | 6/17 | 393/18614 | 8.67E-07 | 5.12E-05 | 2.26E-05 | CCL5/CCL2/CXCL1/CXCR4/S100A8/ITGB2 | 6 |
| BP | GO:0070098 | chemokine-mediated signaling pathway | 4/17 | 91/18614 | 1.21E-06 | 6.74E-05 | 2.97E-05 | CCL5/CCL2/CXCL1/CXCR4 | 4 |
| BP | GO:0071222 | cellular response to lipopolysaccharide | 5/17 | 224/18614 | 1.33E-06 | 6.81E-05 | 3.00E-05 | CASP1/LTF/CCL5/CCL2/CXCL1 | 5 |
| BP | GO:0052547 | regulation of peptidase activity | 6/17 | 425/18614 | 1.37E-06 | 6.81E-05 | 3.00E-05 | CASP1/LTF/TIMP1/CLEC7A/S100A8/F3 | 6 |
| BP | GO:0002218 | activation of innate immune response | 5/17 | 233/18614 | 1.61E-06 | 6.91E-05 | 3.04E-05 | CASP1/MNDA/LTF/TLR7/CLEC7A | 5 |
| BP | GO:2000116 | regulation of cysteine-type endopeptidase activity | 5/17 | 234/18614 | 1.65E-06 | 6.91E-05 | 3.04E-05 | CASP1/LTF/CLEC7A/S100A8/F3 | 5 |
| BP | GO:1990868 | response to chemokine | 4/17 | 99/18614 | 1.70E-06 | 6.91E-05 | 3.04E-05 | CCL5/CCL2/CXCL1/CXCR4 | 4 |
| BP | GO:1990869 | cellular response to chemokine | 4/17 | 99/18614 | 1.70E-06 | 6.91E-05 | 3.04E-05 | CCL5/CCL2/CXCL1/CXCR4 | 4 |
| BP | GO:0071219 | cellular response to molecule of bacterial origin | 5/17 | 237/18614 | 1.75E-06 | 6.91E-05 | 3.04E-05 | CASP1/LTF/CCL5/CCL2/CXCL1 | 5 |
| BP | GO:0097529 | myeloid leukocyte migration | 5/17 | 241/18614 | 1.90E-06 | 7.20E-05 | 3.17E-05 | CCL5/CCL2/CXCL1/S100A8/ITGB2 | 5 |
| BP | GO:0006959 | humoral immune response | 5/17 | 250/18614 | 2.28E-06 | 8.29E-05 | 3.65E-05 | LYZ/C3/LTF/CCL2/CXCL1 | 5 |
| BP | GO:0001819 | positive regulation of cytokine production | 6/17 | 489/18614 | 3.08E-06 | 0.000108056 | 4.76E-05 | CASP1/MNDA/C3/TLR7/CLEC7A/F3 | 6 |
| BP | GO:0002253 | activation of immune response | 6/17 | 495/18614 | 3.31E-06 | 0.000111803 | 4.93E-05 | CASP1/MNDA/C3/LTF/TLR7/CLEC7A | 6 |
| BP | GO:0043280 | positive regulation of cysteine-type endopeptidase activity involved in apoptotic process | 4/17 | 124/18614 | 4.18E-06 | 0.000136193 | 6.00E-05 | CASP1/CLEC7A/S100A8/F3 | 4 |
| BP | GO:2001056 | positive regulation of cysteine-type endopeptidase activity | 4/17 | 142/18614 | 7.15E-06 | 0.000225542 | 9.94E-05 | CASP1/CLEC7A/S100A8/F3 | 4 |
| BP | GO:0050729 | positive regulation of inflammatory response | 4/17 | 154/18614 | 9.86E-06 | 0.000300917 | 0.000132595 | CASP1/C3/TLR7/S100A8 | 4 |
| BP | GO:0031640 | killing of cells of another organism | 3/17 | 49/18614 | 1.14E-05 | 0.000335784 | 0.000147959 | LYZ/LTF/CLEC7A | 3 |
| BP | GO:0010950 | positive regulation of endopeptidase activity | 4/17 | 171/18614 | 1.49E-05 | 0.000427329 | 0.000188297 | CASP1/CLEC7A/S100A8/F3 | 4 |
| BP | GO:0050832 | defense response to fungus | 3/17 | 56/18614 | 1.70E-05 | 0.000473614 | 0.000208692 | LTF/CLEC7A/S100A8 | 3 |
| BP | GO:0002697 | regulation of immune effector process | 5/17 | 384/18614 | 1.84E-05 | 0.000496278 | 0.000218678 | C3/TLR7/CLEC7A/FGL2/ITGB2 | 5 |
| BP | GO:0010952 | positive regulation of peptidase activity | 4/17 | 185/18614 | 2.03E-05 | 0.000533868 | 0.000235242 | CASP1/CLEC7A/S100A8/F3 | 4 |
| BP | GO:0090303 | positive regulation of wound healing | 3/17 | 62/18614 | 2.31E-05 | 0.000573765 | 0.000252822 | CLEC7A/CXCR4/F3 | 3 |
| BP | GO:0032731 | positive regulation of interleukin-1 beta production | 3/17 | 63/18614 | 2.43E-05 | 0.000573765 | 0.000252822 | CASP1/MNDA/CLEC7A | 3 |
| BP | GO:0032757 | positive regulation of interleukin-8 production | 3/17 | 63/18614 | 2.43E-05 | 0.000573765 | 0.000252822 | TLR7/CLEC7A/F3 | 3 |
| BP | GO:0009615 | response to virus | 5/17 | 408/18614 | 2.46E-05 | 0.000573765 | 0.000252822 | CASP1/TLR7/CCL5/CXCR4/FGL2 | 5 |
| BP | GO:0001906 | cell killing | 4/17 | 197/18614 | 2.60E-05 | 0.000573765 | 0.000252822 | LYZ/C3/LTF/CLEC7A | 4 |
| BP | GO:0050727 | regulation of inflammatory response | 5/17 | 414/18614 | 2.64E-05 | 0.000573765 | 0.000252822 | CASP1/C3/TLR7/CCL5/S100A8 | 5 |
| BP | GO:0007159 | leukocyte cell-cell adhesion | 5/17 | 415/18614 | 2.67E-05 | 0.000573765 | 0.000252822 | CCL5/CCL2/FGL2/S100A8/ITGB2 | 5 |
| BP | GO:0031663 | lipopolysaccharide-mediated signaling pathway | 3/17 | 65/18614 | 2.67E-05 | 0.000573765 | 0.000252822 | LTF/CCL5/CCL2 | 3 |
| BP | GO:0002757 | immune response-activating signaling pathway | 5/17 | 423/18614 | 2.92E-05 | 0.000600024 | 0.000264393 | CASP1/MNDA/LTF/TLR7/CLEC7A | 5 |
| BP | GO:0009620 | response to fungus | 3/17 | 67/18614 | 2.92E-05 | 0.000600024 | 0.000264393 | LTF/CLEC7A/S100A8 | 3 |
| BP | GO:0043281 | regulation of cysteine-type endopeptidase activity involved in apoptotic process | 4/17 | 204/18614 | 2.98E-05 | 0.000600024 | 0.000264393 | CASP1/CLEC7A/S100A8/F3 | 4 |
| BP | GO:0002758 | innate immune response-activating signaling pathway | 4/17 | 209/18614 | 3.28E-05 | 0.000645922 | 0.000284617 | CASP1/LTF/TLR7/CLEC7A | 4 |
| BP | GO:0042060 | wound healing | 5/17 | 439/18614 | 3.49E-05 | 0.000652498 | 0.000287514 | TIMP1/CLEC7A/CXCR4/S100A8/F3 | 5 |
| BP | GO:0035747 | natural killer cell chemotaxis | 2/17 | 10/18614 | 3.52E-05 | 0.000652498 | 0.000287514 | CCL5/CCL2 | 2 |
| BP | GO:2000425 | regulation of apoptotic cell clearance | 2/17 | 10/18614 | 3.52E-05 | 0.000652498 | 0.000287514 | C3/CCL2 | 2 |
| BP | GO:0050766 | positive regulation of phagocytosis | 3/17 | 73/18614 | 3.78E-05 | 0.00068818 | 0.000303237 | C3/CCL2/CLEC7A | 3 |
| BP | GO:0002764 | immune response-regulating signaling pathway | 5/17 | 450/18614 | 3.93E-05 | 0.000690303 | 0.000304173 | CASP1/MNDA/LTF/TLR7/CLEC7A | 5 |
| BP | GO:0032732 | positive regulation of interleukin-1 production | 3/17 | 74/18614 | 3.94E-05 | 0.000690303 | 0.000304173 | CASP1/MNDA/CLEC7A | 3 |
| BP | GO:1903036 | positive regulation of response to wounding | 3/17 | 76/18614 | 4.27E-05 | 0.000734176 | 0.000323505 | CLEC7A/CXCR4/F3 | 3 |
| BP | GO:0050920 | regulation of chemotaxis | 4/17 | 230/18614 | 4.76E-05 | 0.000804616 | 0.000354543 | CCL5/CCL2/CXCR4/F3 | 4 |
| BP | GO:0006909 | phagocytosis | 4/17 | 236/18614 | 5.27E-05 | 0.000873909 | 0.000385076 | C3/CCL2/CLEC7A/ITGB2 | 4 |
| BP | GO:0019221 | cytokine-mediated signaling pathway | 5/17 | 492/18614 | 6.01E-05 | 0.000980597 | 0.000432087 | CASP1/CCL5/CCL2/CXCL1/CXCR4 | 5 |
| BP | GO:0002699 | positive regulation of immune effector process | 4/17 | 267/18614 | 8.50E-05 | 0.001363374 | 0.000600752 | C3/TLR7/CLEC7A/ITGB2 | 4 |
| BP | GO:0050764 | regulation of phagocytosis | 3/17 | 101/18614 | 9.98E-05 | 0.00157301 | 0.000693126 | C3/CCL2/CLEC7A | 3 |
| BP | GO:0032677 | regulation of interleukin-8 production | 3/17 | 103/18614 | 0.000105756 | 0.001640079 | 0.000722678 | TLR7/CLEC7A/F3 | 3 |
| BP | GO:0032637 | interleukin-8 production | 3/17 | 104/18614 | 0.000108835 | 0.001660617 | 0.000731729 | TLR7/CLEC7A/F3 | 3 |
| BP | GO:0007249 | I-kappaB kinase/NF-kappaB signaling | 4/17 | 293/18614 | 0.000121766 | 0.001828424 | 0.00080567 | CASP1/LTF/TLR7/CLEC7A | 4 |
| BP | GO:0032930 | positive regulation of superoxide anion generation | 2/17 | 19/18614 | 0.000133028 | 0.001943652 | 0.000856444 | CLEC7A/ITGB2 | 2 |
| BP | GO:0032611 | interleukin-1 beta production | 3/17 | 112/18614 | 0.000135604 | 0.001943652 | 0.000856444 | CASP1/MNDA/CLEC7A | 3 |
| BP | GO:0032651 | regulation of interleukin-1 beta production | 3/17 | 112/18614 | 0.000135604 | 0.001943652 | 0.000856444 | CASP1/MNDA/CLEC7A | 3 |
| BP | GO:0002526 | acute inflammatory response | 3/17 | 114/18614 | 0.000142905 | 0.002017738 | 0.000889089 | C3/S100A8/F3 | 3 |
| BP | GO:0002544 | chronic inflammatory response | 2/17 | 20/18614 | 0.000147729 | 0.002055173 | 0.000905584 | CCL5/S100A8 | 2 |
| BP | GO:0071346 | cellular response to type II interferon | 3/17 | 116/18614 | 0.000150459 | 0.002062811 | 0.00090895 | CASP1/CCL5/CCL2 | 3 |
| BP | GO:0002888 | positive regulation of myeloid leukocyte mediated immunity | 2/17 | 21/18614 | 0.000163192 | 0.002205422 | 0.000971789 | C3/ITGB2 | 2 |
| BP | GO:0019058 | viral life cycle | 4/17 | 320/18614 | 0.000170929 | 0.00227745 | 0.001003527 | LTF/CCL5/CCL2/CXCR4 | 4 |
| BP | GO:0032928 | regulation of superoxide anion generation | 2/17 | 22/18614 | 0.000179415 | 0.002357309 | 0.001038716 | CLEC7A/ITGB2 | 2 |
| BP | GO:0019730 | antimicrobial humoral response | 3/17 | 126/18614 | 0.000192135 | 0.00248986 | 0.001097123 | LYZ/LTF/CXCL1 | 3 |
| BP | GO:0019079 | viral genome replication | 3/17 | 129/18614 | 0.000205955 | 0.002632883 | 0.001160144 | LTF/CCL5/CCL2 | 3 |
| BP | GO:0032612 | interleukin-1 production | 3/17 | 131/18614 | 0.000215518 | 0.002682626 | 0.001182063 | CASP1/MNDA/CLEC7A | 3 |
| BP | GO:0032652 | regulation of interleukin-1 production | 3/17 | 131/18614 | 0.000215518 | 0.002682626 | 0.001182063 | CASP1/MNDA/CLEC7A | 3 |
| BP | GO:0061041 | regulation of wound healing | 3/17 | 137/18614 | 0.000245924 | 0.003016201 | 0.001329048 | CLEC7A/CXCR4/F3 | 3 |
| BP | GO:0002407 | dendritic cell chemotaxis | 2/17 | 26/18614 | 0.000251881 | 0.003016201 | 0.001329048 | CCL5/CXCR4 | 2 |
| BP | GO:1901623 | regulation of lymphocyte chemotaxis | 2/17 | 26/18614 | 0.000251881 | 0.003016201 | 0.001329048 | CCL5/CCL2 | 2 |
| BP | GO:0034341 | response to type II interferon | 3/17 | 139/18614 | 0.000256644 | 0.003034813 | 0.001337249 | CASP1/CCL5/CCL2 | 3 |
| BP | GO:0050921 | positive regulation of chemotaxis | 3/17 | 143/18614 | 0.000278984 | 0.003258254 | 0.001435706 | CCL5/CXCR4/F3 | 3 |
| BP | GO:0045862 | positive regulation of proteolysis | 4/17 | 365/18614 | 0.000282746 | 0.003261928 | 0.001437325 | CASP1/CLEC7A/S100A8/F3 | 4 |
| BP | GO:0002705 | positive regulation of leukocyte mediated immunity | 3/17 | 148/18614 | 0.000308632 | 0.003517661 | 0.00155001 | C3/CLEC7A/ITGB2 | 3 |
| BP | GO:0048245 | eosinophil chemotaxis | 2/17 | 29/18614 | 0.000314152 | 0.003537944 | 0.001558947 | CCL5/CCL2 | 2 |
| BP | GO:0002696 | positive regulation of leukocyte activation | 4/17 | 377/18614 | 0.000319804 | 0.00355347 | 0.001565789 | CCL5/CCL2/CLEC7A/ITGB2 | 4 |
| BP | GO:1903037 | regulation of leukocyte cell-cell adhesion | 4/17 | 378/18614 | 0.000323043 | 0.00355347 | 0.001565789 | CCL5/CCL2/FGL2/ITGB2 | 4 |
| BP | GO:0050867 | positive regulation of cell activation | 4/17 | 394/18614 | 0.000378133 | 0.004094242 | 0.001804072 | CCL5/CCL2/CLEC7A/ITGB2 | 4 |
| BP | GO:0051092 | positive regulation of NF-kappaB transcription factor activity | 3/17 | 159/18614 | 0.00038086 | 0.004094242 | 0.001804072 | LTF/CLEC7A/S100A8 | 3 |
| BP | GO:0036336 | dendritic cell migration | 2/17 | 33/18614 | 0.000407675 | 0.004285118 | 0.001888179 | CCL5/CXCR4 | 2 |
| BP | GO:0072677 | eosinophil migration | 2/17 | 33/18614 | 0.000407675 | 0.004285118 | 0.001888179 | CCL5/CCL2 | 2 |
| BP | GO:0001959 | regulation of cytokine-mediated signaling pathway | 3/17 | 165/18614 | 0.000424478 | 0.004364739 | 0.001923263 | CASP1/CCL5/CXCR4 | 3 |
| BP | GO:0050792 | regulation of viral process | 3/17 | 165/18614 | 0.000424478 | 0.004364739 | 0.001923263 | LTF/CCL5/CXCR4 | 3 |
| BP | GO:0090322 | regulation of superoxide metabolic process | 2/17 | 34/18614 | 0.000432922 | 0.004403704 | 0.001940432 | CLEC7A/ITGB2 | 2 |
| BP | GO:1903034 | regulation of response to wounding | 3/17 | 170/18614 | 0.000463189 | 0.004661454 | 0.002054007 | CLEC7A/CXCR4/F3 | 3 |
| BP | GO:0016032 | viral process | 4/17 | 426/18614 | 0.000508117 | 0.005059779 | 0.002229523 | LTF/CCL5/CCL2/CXCR4 | 4 |
| BP | GO:0060759 | regulation of response to cytokine stimulus | 3/17 | 177/18614 | 0.000521099 | 0.005134996 | 0.002262667 | CASP1/CCL5/CXCR4 | 3 |
| BP | GO:0097242 | amyloid-beta clearance | 2/17 | 39/18614 | 0.000570295 | 0.005561843 | 0.002450751 | C3/ITGB2 | 2 |
| BP | GO:0045766 | positive regulation of angiogenesis | 3/17 | 185/18614 | 0.000592754 | 0.005664095 | 0.002495807 | C3/CXCR4/F3 | 3 |
| BP | GO:1904018 | positive regulation of vasculature development | 3/17 | 185/18614 | 0.000592754 | 0.005664095 | 0.002495807 | C3/CXCR4/F3 | 3 |
| BP | GO:0048246 | macrophage chemotaxis | 2/17 | 41/18614 | 0.000630418 | 0.005908589 | 0.00260354 | CCL5/CCL2 | 2 |
| BP | GO:0002221 | pattern recognition receptor signaling pathway | 3/17 | 189/18614 | 0.000630832 | 0.005908589 | 0.00260354 | CASP1/LTF/TLR7 | 3 |
| BP | GO:0043123 | positive regulation of I-kappaB kinase/NF-kappaB signaling | 3/17 | 192/18614 | 0.000660397 | 0.006076292 | 0.002677436 | CASP1/LTF/CLEC7A | 3 |
| BP | GO:0042119 | neutrophil activation | 2/17 | 42/18614 | 0.000661584 | 0.006076292 | 0.002677436 | CCL5/ITGB2 | 2 |
| BP | GO:0042554 | superoxide anion generation | 2/17 | 43/18614 | 0.000693484 | 0.006308034 | 0.00277955 | CLEC7A/ITGB2 | 2 |
| BP | GO:0071674 | mononuclear cell migration | 3/17 | 202/18614 | 0.000765317 | 0.00689514 | 0.00303825 | CCL5/CCL2/CXCR4 | 3 |
| BP | GO:0043277 | apoptotic cell clearance | 2/17 | 47/18614 | 0.000828403 | 0.007393105 | 0.003257672 | C3/CCL2 | 2 |
| BP | GO:0002683 | negative regulation of immune system process | 4/17 | 487/18614 | 0.000839973 | 0.007426306 | 0.003272301 | MNDA/LTF/CCL2/FGL2 | 4 |
| BP | GO:0036230 | granulocyte activation | 2/17 | 48/18614 | 0.000863957 | 0.007516287 | 0.00331195 | CCL5/ITGB2 | 2 |
| BP | GO:0022407 | regulation of cell-cell adhesion | 4/17 | 491/18614 | 0.000866042 | 0.007516287 | 0.00331195 | CCL5/CCL2/FGL2/ITGB2 | 4 |
| BP | GO:0006911 | phagocytosis, engulfment | 2/17 | 49/18614 | 0.000900237 | 0.007742041 | 0.003411426 | C3/ITGB2 | 2 |
| BP | GO:0009612 | response to mechanical stimulus | 3/17 | 215/18614 | 0.000916868 | 0.007744257 | 0.003412402 | CASP1/TLR7/CXCR4 | 3 |
| BP | GO:0043491 | protein kinase B signaling | 3/17 | 215/18614 | 0.000916868 | 0.007744257 | 0.003412402 | CCL5/CCL2/F3 | 3 |
| BP | GO:0045071 | negative regulation of viral genome replication | 2/17 | 56/18614 | 0.001174462 | 0.009832218 | 0.004332434 | LTF/CCL5 | 2 |
| BP | GO:0071356 | cellular response to tumor necrosis factor | 3/17 | 235/18614 | 0.001185244 | 0.009835449 | 0.004333858 | CASP1/CCL5/CCL2 | 3 |
| BP | GO:0099024 | plasma membrane invagination | 2/17 | 58/18614 | 0.001259287 | 0.010359007 | 0.004564556 | C3/ITGB2 | 2 |
| BP | GO:0001961 | positive regulation of cytokine-mediated signaling pathway | 2/17 | 59/18614 | 0.001302774 | 0.010624343 | 0.004681473 | CASP1/CXCR4 | 2 |
| BP | GO:0022604 | regulation of cell morphogenesis | 3/17 | 245/18614 | 0.001336217 | 0.010747433 | 0.004735711 | CCL2/CXCR4/ITGB2 | 3 |
| BP | GO:0002886 | regulation of myeloid leukocyte mediated immunity | 2/17 | 60/18614 | 0.001346974 | 0.010747433 | 0.004735711 | C3/ITGB2 | 2 |
| BP | GO:0002703 | regulation of leukocyte mediated immunity | 3/17 | 246/18614 | 0.00135195 | 0.010747433 | 0.004735711 | C3/CLEC7A/ITGB2 | 3 |
| BP | GO:0071695 | anatomical structure maturation | 3/17 | 247/18614 | 0.001367799 | 0.010782817 | 0.004751303 | C3/LTF/CLEC7A | 3 |
| BP | GO:1905517 | macrophage migration | 2/17 | 61/18614 | 0.001391887 | 0.010882025 | 0.004795017 | CCL5/CCL2 | 2 |
| BP | GO:0048247 | lymphocyte chemotaxis | 2/17 | 64/18614 | 0.001530891 | 0.011693741 | 0.005152689 | CCL5/CCL2 | 2 |
| BP | GO:0050918 | positive chemotaxis | 2/17 | 64/18614 | 0.001530891 | 0.011693741 | 0.005152689 | CCL5/F3 | 2 |
| BP | GO:0034612 | response to tumor necrosis factor | 3/17 | 257/18614 | 0.001532795 | 0.011693741 | 0.005152689 | CASP1/CCL5/CCL2 | 3 |
| BP | GO:0043122 | regulation of I-kappaB kinase/NF-kappaB signaling | 3/17 | 258/18614 | 0.001549952 | 0.011730035 | 0.005168681 | CASP1/LTF/CLEC7A | 3 |
| BP | GO:0010324 | membrane invagination | 2/17 | 65/18614 | 0.001578644 | 0.011852357 | 0.005222581 | C3/ITGB2 | 2 |
| BP | GO:0060760 | positive regulation of response to cytokine stimulus | 2/17 | 66/18614 | 0.001627103 | 0.012119997 | 0.005340513 | CASP1/CXCR4 | 2 |
| BP | GO:0051924 | regulation of calcium ion transport | 3/17 | 266/18614 | 0.001691586 | 0.012501875 | 0.005508782 | CCL5/CCL2/CXCR4 | 3 |
| BP | GO:2000401 | regulation of lymphocyte migration | 2/17 | 68/18614 | 0.001726138 | 0.012658346 | 0.005577729 | CCL5/CCL2 | 2 |
| BP | GO:0051091 | positive regulation of DNA-binding transcription factor activity | 3/17 | 271/18614 | 0.001784108 | 0.012982815 | 0.005720702 | LTF/CLEC7A/S100A8 | 3 |
| BP | GO:1903039 | positive regulation of leukocyte cell-cell adhesion | 3/17 | 273/18614 | 0.00182199 | 0.01300207 | 0.005729186 | CCL5/CCL2/ITGB2 | 3 |
| BP | GO:0032722 | positive regulation of chemokine production | 2/17 | 70/18614 | 0.001827987 | 0.01300207 | 0.005729186 | TLR7/CLEC7A | 2 |
| BP | GO:2000379 | positive regulation of reactive oxygen species metabolic process | 2/17 | 70/18614 | 0.001827987 | 0.01300207 | 0.005729186 | CLEC7A/ITGB2 | 2 |
| BP | GO:0002548 | monocyte chemotaxis | 2/17 | 71/18614 | 0.001879963 | 0.013173666 | 0.005804798 | CCL5/CCL2 | 2 |
| BP | GO:0072678 | T cell migration | 2/17 | 71/18614 | 0.001879963 | 0.013173666 | 0.005804798 | CCL5/CCL2 | 2 |
| BP | GO:0071260 | cellular response to mechanical stimulus | 2/17 | 73/18614 | 0.001986014 | 0.013814481 | 0.006087164 | CASP1/TLR7 | 2 |
| BP | GO:0006801 | superoxide metabolic process | 2/17 | 74/18614 | 0.002040087 | 0.014087022 | 0.006207256 | CLEC7A/ITGB2 | 2 |
| BP | GO:0006919 | activation of cysteine-type endopeptidase activity involved in apoptotic process | 2/17 | 76/18614 | 0.00215032 | 0.014740597 | 0.006495245 | S100A8/F3 | 2 |
| BP | GO:0032729 | positive regulation of type II interferon production | 2/17 | 77/18614 | 0.002206478 | 0.015016748 | 0.006616927 | TLR7/CLEC7A | 2 |
| BP | GO:0002220 | innate immune response activating cell surface receptor signaling pathway | 2/17 | 78/18614 | 0.002263329 | 0.015293636 | 0.006738934 | LTF/CLEC7A | 2 |
| BP | GO:0001895 | retina homeostasis | 2/17 | 80/18614 | 0.002379105 | 0.015849529 | 0.006983881 | LYZ/LTF | 2 |
| BP | GO:0002720 | positive regulation of cytokine production involved in immune response | 2/17 | 80/18614 | 0.002379105 | 0.015849529 | 0.006983881 | TLR7/CLEC7A | 2 |
| BP | GO:0002429 | immune response-activating cell surface receptor signaling pathway | 3/17 | 302/18614 | 0.00242898 | 0.016068638 | 0.007080428 | MNDA/LTF/CLEC7A | 3 |
| BP | GO:0051607 | defense response to virus | 3/17 | 303/18614 | 0.002451884 | 0.016107517 | 0.007097559 | CASP1/TLR7/FGL2 | 3 |
| BP | GO:0140546 | defense response to symbiont | 3/17 | 304/18614 | 0.002474923 | 0.016146738 | 0.007114842 | CASP1/TLR7/FGL2 | 3 |
| BP | GO:0061844 | antimicrobial humoral immune response mediated by antimicrobial peptide | 2/17 | 82/18614 | 0.002497638 | 0.016183328 | 0.007130965 | LTF/CXCL1 | 2 |
| BP | GO:0051851 | modulation by host of symbiont process | 2/17 | 83/18614 | 0.002557936 | 0.016461278 | 0.007253439 | LTF/CCL5 | 2 |
| BP | GO:0098586 | cellular response to virus | 2/17 | 85/18614 | 0.002680589 | 0.017068456 | 0.007520984 | TLR7/CCL5 | 2 |
| BP | GO:0044403 | biological process involved in symbiotic interaction | 3/17 | 313/18614 | 0.002688372 | 0.017068456 | 0.007520984 | LTF/CCL5/CXCR4 | 3 |
| BP | GO:0042742 | defense response to bacterium | 3/17 | 314/18614 | 0.002712772 | 0.017108546 | 0.007538649 | LYZ/LTF/S100A8 | 3 |
| BP | GO:0045069 | regulation of viral genome replication | 2/17 | 86/18614 | 0.002742942 | 0.017184256 | 0.00757201 | LTF/CCL5 | 2 |
| BP | GO:0033627 | cell adhesion mediated by integrin | 2/17 | 87/18614 | 0.002805976 | 0.017463511 | 0.00769506 | CCL5/ITGB2 | 2 |
| BP | GO:0021700 | developmental maturation | 3/17 | 319/18614 | 0.00283684 | 0.017540197 | 0.007728851 | C3/LTF/CLEC7A | 3 |
| BP | GO:0042063 | gliogenesis | 3/17 | 320/18614 | 0.002862069 | 0.017581281 | 0.007746954 | CCL2/CXCR4/S100A8 | 3 |
| BP | GO:0022409 | positive regulation of cell-cell adhesion | 3/17 | 321/18614 | 0.002887438 | 0.017622684 | 0.007765197 | CCL5/CCL2/ITGB2 | 3 |
| BP | GO:0048525 | negative regulation of viral process | 2/17 | 90/18614 | 0.002999164 | 0.018071399 | 0.007962917 | LTF/CCL5 | 2 |
| BP | GO:0050829 | defense response to Gram-negative bacterium | 2/17 | 90/18614 | 0.002999164 | 0.018071399 | 0.007962917 | LYZ/LTF | 2 |
| BP | GO:0051251 | positive regulation of lymphocyte activation | 3/17 | 327/18614 | 0.003042589 | 0.018102447 | 0.007976598 | CCL5/CCL2/CLEC7A | 3 |
| BP | GO:1901214 | regulation of neuron death | 3/17 | 327/18614 | 0.003042589 | 0.018102447 | 0.007976598 | CCL5/CCL2/ITGB2 | 3 |
| BP | GO:0002768 | immune response-regulating cell surface receptor signaling pathway | 3/17 | 329/18614 | 0.003095432 | 0.018301741 | 0.008064415 | MNDA/LTF/CLEC7A | 3 |
| BP | GO:0071214 | cellular response to abiotic stimulus | 3/17 | 339/18614 | 0.003368186 | 0.019668543 | 0.008666677 | CASP1/TIMP1/TLR7 | 3 |
| BP | GO:0104004 | cellular response to environmental stimulus | 3/17 | 339/18614 | 0.003368186 | 0.019668543 | 0.008666677 | CASP1/TIMP1/TLR7 | 3 |
| BP | GO:0032642 | regulation of chemokine production | 2/17 | 98/18614 | 0.003544076 | 0.020568689 | 0.009063314 | TLR7/CLEC7A | 2 |
| BP | GO:0032602 | chemokine production | 2/17 | 99/18614 | 0.003615212 | 0.0208536 | 0.009188857 | TLR7/CLEC7A | 2 |
| BP | GO:0045765 | regulation of angiogenesis | 3/17 | 349/18614 | 0.003655358 | 0.020957383 | 0.009234587 | C3/CXCR4/F3 | 3 |
| BP | GO:0032755 | positive regulation of interleukin-6 production | 2/17 | 100/18614 | 0.003687015 | 0.021011543 | 0.009258452 | TLR7/CLEC7A | 2 |
| BP | GO:1901342 | regulation of vasculature development | 3/17 | 355/18614 | 0.003834684 | 0.021722222 | 0.009571603 | C3/CXCR4/F3 | 3 |
| BP | GO:0019233 | sensory perception of pain | 2/17 | 103/18614 | 0.003906419 | 0.021996859 | 0.009692618 | CCL2/CXCR4 | 2 |
| BP | GO:1902106 | negative regulation of leukocyte differentiation | 2/17 | 109/18614 | 0.004363101 | 0.024423039 | 0.010761682 | LTF/FGL2 | 2 |
| BP | GO:0070997 | neuron death | 3/17 | 373/18614 | 0.004404854 | 0.024511714 | 0.010800755 | CCL5/CCL2/ITGB2 | 3 |
| BP | GO:0002444 | myeloid leukocyte mediated immunity | 2/17 | 110/18614 | 0.004441519 | 0.024571209 | 0.010826971 | C3/ITGB2 | 2 |
| BP | GO:0071347 | cellular response to interleukin-1 | 2/17 | 111/18614 | 0.004520592 | 0.024815876 | 0.01093478 | CCL5/CCL2 | 2 |
| BP | GO:0050863 | regulation of T cell activation | 3/17 | 377/18614 | 0.00453821 | 0.024815876 | 0.01093478 | CCL5/CCL2/FGL2 | 3 |
| BP | GO:0007229 | integrin-mediated signaling pathway | 2/17 | 112/18614 | 0.004600319 | 0.025010928 | 0.011020727 | TIMP1/ITGB2 | 2 |
| BP | GO:0044344 | cellular response to fibroblast growth factor stimulus | 2/17 | 113/18614 | 0.004680699 | 0.02530252 | 0.011149213 | CCL5/CCL2 | 2 |
| BP | GO:1903707 | negative regulation of hemopoiesis | 2/17 | 115/18614 | 0.004843414 | 0.026033348 | 0.011471243 | LTF/FGL2 | 2 |
| BP | GO:0032609 | type II interferon production | 2/17 | 118/18614 | 0.005092356 | 0.026912674 | 0.011858706 | TLR7/CLEC7A | 2 |
| BP | GO:0032649 | regulation of type II interferon production | 2/17 | 118/18614 | 0.005092356 | 0.026912674 | 0.011858706 | TLR7/CLEC7A | 2 |
| BP | GO:0051702 | biological process involved in interaction with symbiont | 2/17 | 118/18614 | 0.005092356 | 0.026912674 | 0.011858706 | LTF/CCL5 | 2 |
| BP | GO:0002718 | regulation of cytokine production involved in immune response | 2/17 | 119/18614 | 0.005176631 | 0.027206071 | 0.011987987 | TLR7/CLEC7A | 2 |
| BP | GO:0071774 | response to fibroblast growth factor | 2/17 | 120/18614 | 0.005261551 | 0.027499599 | 0.012117326 | CCL5/CCL2 | 2 |
| BP | GO:0002367 | cytokine production involved in immune response | 2/17 | 121/18614 | 0.005347116 | 0.027783371 | 0.012242366 | TLR7/CLEC7A | 2 |
| BP | GO:0002443 | leukocyte mediated immunity | 3/17 | 401/18614 | 0.005390278 | 0.027783371 | 0.012242366 | C3/CLEC7A/ITGB2 | 3 |
| BP | GO:0071675 | regulation of mononuclear cell migration | 2/17 | 122/18614 | 0.005433323 | 0.027783371 | 0.012242366 | CCL5/CCL2 | 2 |
| BP | GO:0072676 | lymphocyte migration | 2/17 | 122/18614 | 0.005433323 | 0.027783371 | 0.012242366 | CCL5/CCL2 | 2 |
| BP | GO:0002824 | positive regulation of adaptive immune response based on somatic recombination of immune receptors built from immunoglobulin superfamily domains | 2/17 | 123/18614 | 0.005520172 | 0.028075716 | 0.012371184 | C3/CLEC7A | 2 |
| BP | GO:0002832 | negative regulation of response to biotic stimulus | 2/17 | 125/18614 | 0.005695793 | 0.02881401 | 0.012696504 | LTF/FGL2 | 2 |
| BP | GO:0002688 | regulation of leukocyte chemotaxis | 2/17 | 126/18614 | 0.005784561 | 0.029107421 | 0.012825791 | CCL5/CCL2 | 2 |
| BP | GO:0002821 | positive regulation of adaptive immune response | 2/17 | 129/18614 | 0.006054688 | 0.030145974 | 0.013283416 | C3/CLEC7A | 2 |
| BP | GO:0051928 | positive regulation of calcium ion transport | 2/17 | 129/18614 | 0.006054688 | 0.030145974 | 0.013283416 | CCL5/CCL2 | 2 |
| BP | GO:0010959 | regulation of metal ion transport | 3/17 | 423/18614 | 0.006251284 | 0.030961858 | 0.013642924 | CCL5/CCL2/CXCR4 | 3 |
| BP | GO:0002702 | positive regulation of production of molecular mediator of immune response | 2/17 | 134/18614 | 0.006517571 | 0.032112614 | 0.014149989 | TLR7/CLEC7A | 2 |
| BP | GO:0008277 | regulation of G protein-coupled receptor signaling pathway | 2/17 | 137/18614 | 0.006802854 | 0.033344561 | 0.01469283 | C3/CCL5 | 2 |
| BP | GO:0008360 | regulation of cell shape | 2/17 | 138/18614 | 0.006899202 | 0.033642499 | 0.014824112 | CCL2/ITGB2 | 2 |
| BP | GO:0070555 | response to interleukin-1 | 2/17 | 141/18614 | 0.007191986 | 0.034890351 | 0.015373961 | CCL5/CCL2 | 2 |
| BP | GO:0032102 | negative regulation of response to external stimulus | 3/17 | 446/18614 | 0.007235474 | 0.034922238 | 0.015388012 | LTF/CCL2/FGL2 | 3 |
| BP | GO:1903900 | regulation of viral life cycle | 2/17 | 143/18614 | 0.007390283 | 0.035488364 | 0.015637468 | LTF/CCL5 | 2 |
| BP | GO:0006816 | calcium ion transport | 3/17 | 455/18614 | 0.007644439 | 0.036523429 | 0.016093555 | CCL5/CCL2/CXCR4 | 3 |
| BP | GO:2000377 | regulation of reactive oxygen species metabolic process | 2/17 | 150/18614 | 0.008103768 | 0.038382983 | 0.016912942 | CLEC7A/ITGB2 | 2 |
| BP | GO:0051090 | regulation of DNA-binding transcription factor activity | 3/17 | 465/18614 | 0.008114795 | 0.038382983 | 0.016912942 | LTF/CLEC7A/S100A8 | 3 |
| BP | GO:0008037 | cell recognition | 2/17 | 155/18614 | 0.008631769 | 0.040625142 | 0.017900919 | CLEC7A/CXCR4 | 2 |
| BP | GO:0045785 | positive regulation of cell adhesion | 3/17 | 482/18614 | 0.008953366 | 0.041930119 | 0.01847594 | CCL5/CCL2/ITGB2 | 3 |
| BP | GO:0002291 | T cell activation via T cell receptor contact with antigen bound to MHC molecule on antigen presenting cell | 1/17 | 10/18614 | 0.009097658 | 0.041982363 | 0.018498961 | FGL2 | 1 |
| BP | GO:0002676 | regulation of chronic inflammatory response | 1/17 | 10/18614 | 0.009097658 | 0.041982363 | 0.018498961 | CCL5 | 1 |
| BP | GO:0070099 | regulation of chemokine-mediated signaling pathway | 1/17 | 10/18614 | 0.009097658 | 0.041982363 | 0.018498961 | CCL5 | 1 |
| BP | GO:0010951 | negative regulation of endopeptidase activity | 2/17 | 160/18614 | 0.009174926 | 0.042133399 | 0.018565513 | LTF/TIMP1 | 2 |
| BP | GO:0030307 | positive regulation of cell growth | 2/17 | 164/18614 | 0.009620276 | 0.043965125 | 0.019372638 | CXCR4/S100A8 | 2 |
| BP | GO:0002604 | regulation of dendritic cell antigen processing and presentation | 1/17 | 11/18614 | 0.010003127 | 0.044219429 | 0.019484693 | FGL2 | 1 |
| BP | GO:0033632 | regulation of cell-cell adhesion mediated by integrin | 1/17 | 11/18614 | 0.010003127 | 0.044219429 | 0.019484693 | CCL5 | 1 |
| BP | GO:0034350 | regulation of glial cell apoptotic process | 1/17 | 11/18614 | 0.010003127 | 0.044219429 | 0.019484693 | CCL2 | 1 |
| BP | GO:0035821 | modulation of process of another organism | 1/17 | 11/18614 | 0.010003127 | 0.044219429 | 0.019484693 | CLEC7A | 1 |
| BP | GO:0071492 | cellular response to UV-A | 1/17 | 11/18614 | 0.010003127 | 0.044219429 | 0.019484693 | TIMP1 | 1 |
| BP | GO:0097278 | complement-dependent cytotoxicity | 1/17 | 11/18614 | 0.010003127 | 0.044219429 | 0.019484693 | C3 | 1 |
| BP | GO:2001204 | regulation of osteoclast development | 1/17 | 11/18614 | 0.010003127 | 0.044219429 | 0.019484693 | LTF | 1 |
| BP | GO:0051250 | negative regulation of lymphocyte activation | 2/17 | 168/18614 | 0.010075173 | 0.044330762 | 0.019533751 | MNDA/FGL2 | 2 |
| BP | GO:0007259 | receptor signaling pathway via JAK-STAT | 2/17 | 173/18614 | 0.01065712 | 0.046481057 | 0.020481249 | CCL5/CCL2 | 2 |
| BP | GO:0002732 | positive regulation of dendritic cell cytokine production | 1/17 | 12/18614 | 0.010907817 | 0.046481057 | 0.020481249 | CLEC7A | 1 |
| BP | GO:0034145 | positive regulation of toll-like receptor 4 signaling pathway | 1/17 | 12/18614 | 0.010907817 | 0.046481057 | 0.020481249 | LTF | 1 |
| BP | GO:0034154 | toll-like receptor 7 signaling pathway | 1/17 | 12/18614 | 0.010907817 | 0.046481057 | 0.020481249 | TLR7 | 1 |
| BP | GO:0043312 | neutrophil degranulation | 1/17 | 12/18614 | 0.010907817 | 0.046481057 | 0.020481249 | ITGB2 | 1 |
| BP | GO:0043380 | regulation of memory T cell differentiation | 1/17 | 12/18614 | 0.010907817 | 0.046481057 | 0.020481249 | FGL2 | 1 |
| BP | GO:0072683 | T cell extravasation | 1/17 | 12/18614 | 0.010907817 | 0.046481057 | 0.020481249 | CCL2 | 1 |
| BP | GO:0032635 | interleukin-6 production | 2/17 | 178/18614 | 0.011253751 | 0.047527 | 0.02094213 | TLR7/CLEC7A | 2 |
| BP | GO:0032675 | regulation of interleukin-6 production | 2/17 | 178/18614 | 0.011253751 | 0.047527 | 0.02094213 | TLR7/CLEC7A | 2 |
| BP | GO:0002524 | hypersensitivity | 1/17 | 13/18614 | 0.011811728 | 0.047843576 | 0.021081624 | C3 | 1 |
| BP | GO:0002863 | positive regulation of inflammatory response to antigenic stimulus | 1/17 | 13/18614 | 0.011811728 | 0.047843576 | 0.021081624 | C3 | 1 |
| BP | GO:0033690 | positive regulation of osteoblast proliferation | 1/17 | 13/18614 | 0.011811728 | 0.047843576 | 0.021081624 | LTF | 1 |
| BP | GO:0043379 | memory T cell differentiation | 1/17 | 13/18614 | 0.011811728 | 0.047843576 | 0.021081624 | FGL2 | 1 |
| BP | GO:0060100 | positive regulation of phagocytosis, engulfment | 1/17 | 13/18614 | 0.011811728 | 0.047843576 | 0.021081624 | C3 | 1 |
| BP | GO:0070234 | positive regulation of T cell apoptotic process | 1/17 | 13/18614 | 0.011811728 | 0.047843576 | 0.021081624 | CCL5 | 1 |
| BP | GO:0070486 | leukocyte aggregation | 1/17 | 13/18614 | 0.011811728 | 0.047843576 | 0.021081624 | S100A8 | 1 |
| BP | GO:1903265 | positive regulation of tumor necrosis factor-mediated signaling pathway | 1/17 | 13/18614 | 0.011811728 | 0.047843576 | 0.021081624 | CASP1 | 1 |
| BP | GO:1905155 | positive regulation of membrane invagination | 1/17 | 13/18614 | 0.011811728 | 0.047843576 | 0.021081624 | C3 | 1 |
| BP | GO:0048469 | cell maturation | 2/17 | 184/18614 | 0.011988907 | 0.047843576 | 0.021081624 | C3/CLEC7A | 2 |
| BP | GO:0097696 | receptor signaling pathway via STAT | 2/17 | 184/18614 | 0.011988907 | 0.047843576 | 0.021081624 | CCL5/CCL2 | 2 |
| BP | GO:0001936 | regulation of endothelial cell proliferation | 2/17 | 185/18614 | 0.012113456 | 0.047843576 | 0.021081624 | CCL2/F3 | 2 |
| BP | GO:0032640 | tumor necrosis factor production | 2/17 | 185/18614 | 0.012113456 | 0.047843576 | 0.021081624 | LTF/CLEC7A | 2 |
| BP | GO:0032680 | regulation of tumor necrosis factor production | 2/17 | 185/18614 | 0.012113456 | 0.047843576 | 0.021081624 | LTF/CLEC7A | 2 |
| BP | GO:0019722 | calcium-mediated signaling | 2/17 | 187/18614 | 0.012364276 | 0.047843576 | 0.021081624 | CLEC7A/CXCR4 | 2 |
| BP | GO:0002700 | regulation of production of molecular mediator of immune response | 2/17 | 189/18614 | 0.012617388 | 0.047843576 | 0.021081624 | TLR7/CLEC7A | 2 |
| BP | GO:0016322 | neuron remodeling | 1/17 | 14/18614 | 0.012714863 | 0.047843576 | 0.021081624 | C3 | 1 |
| BP | GO:0031665 | negative regulation of lipopolysaccharide-mediated signaling pathway | 1/17 | 14/18614 | 0.012714863 | 0.047843576 | 0.021081624 | LTF | 1 |
| BP | GO:0035630 | bone mineralization involved in bone maturation | 1/17 | 14/18614 | 0.012714863 | 0.047843576 | 0.021081624 | LTF | 1 |
| BP | GO:0043922 | negative regulation by host of viral transcription | 1/17 | 14/18614 | 0.012714863 | 0.047843576 | 0.021081624 | CCL5 | 1 |
| BP | GO:0044793 | negative regulation by host of viral process | 1/17 | 14/18614 | 0.012714863 | 0.047843576 | 0.021081624 | LTF | 1 |
| BP | GO:0061043 | regulation of vascular wound healing | 1/17 | 14/18614 | 0.012714863 | 0.047843576 | 0.021081624 | CXCR4 | 1 |
| BP | GO:0070141 | response to UV-A | 1/17 | 14/18614 | 0.012714863 | 0.047843576 | 0.021081624 | TIMP1 | 1 |
| BP | GO:0090715 | immunological memory formation process | 1/17 | 14/18614 | 0.012714863 | 0.047843576 | 0.021081624 | FGL2 | 1 |
| BP | GO:1905049 | negative regulation of metallopeptidase activity | 1/17 | 14/18614 | 0.012714863 | 0.047843576 | 0.021081624 | TIMP1 | 1 |
| BP | GO:0002822 | regulation of adaptive immune response based on somatic recombination of immune receptors built from immunoglobulin superfamily domains | 2/17 | 190/18614 | 0.0127448 | 0.047843576 | 0.021081624 | C3/CLEC7A | 2 |
| BP | GO:0071706 | tumor necrosis factor superfamily cytokine production | 2/17 | 190/18614 | 0.0127448 | 0.047843576 | 0.021081624 | LTF/CLEC7A | 2 |
| BP | GO:1903555 | regulation of tumor necrosis factor superfamily cytokine production | 2/17 | 190/18614 | 0.0127448 | 0.047843576 | 0.021081624 | LTF/CLEC7A | 2 |
| BP | GO:0002791 | regulation of peptide secretion | 2/17 | 195/18614 | 0.013390391 | 0.048372093 | 0.021314508 | CCL5/S100A8 | 2 |
| BP | GO:0002468 | dendritic cell antigen processing and presentation | 1/17 | 15/18614 | 0.01361722 | 0.048372093 | 0.021314508 | FGL2 | 1 |
| BP | GO:0002864 | regulation of acute inflammatory response to antigenic stimulus | 1/17 | 15/18614 | 0.01361722 | 0.048372093 | 0.021314508 | C3 | 1 |
| BP | GO:0010820 | positive regulation of T cell chemotaxis | 1/17 | 15/18614 | 0.01361722 | 0.048372093 | 0.021314508 | CCL5 | 1 |
| BP | GO:0042976 | activation of Janus kinase activity | 1/17 | 15/18614 | 0.01361722 | 0.048372093 | 0.021314508 | CCL5 | 1 |
| BP | GO:0044406 | adhesion of symbiont to host | 1/17 | 15/18614 | 0.01361722 | 0.048372093 | 0.021314508 | LTF | 1 |
| BP | GO:0050966 | detection of mechanical stimulus involved in sensory perception of pain | 1/17 | 15/18614 | 0.01361722 | 0.048372093 | 0.021314508 | CXCR4 | 1 |
| BP | GO:0060099 | regulation of phagocytosis, engulfment | 1/17 | 15/18614 | 0.01361722 | 0.048372093 | 0.021314508 | C3 | 1 |
| BP | GO:0071639 | positive regulation of monocyte chemotactic protein-1 production | 1/17 | 15/18614 | 0.01361722 | 0.048372093 | 0.021314508 | CLEC7A | 1 |
| BP | GO:0090280 | positive regulation of calcium ion import | 1/17 | 15/18614 | 0.01361722 | 0.048372093 | 0.021314508 | CCL2 | 1 |
| BP | GO:0098883 | synapse pruning | 1/17 | 15/18614 | 0.01361722 | 0.048372093 | 0.021314508 | C3 | 1 |
| BP | GO:1902563 | regulation of neutrophil activation | 1/17 | 15/18614 | 0.01361722 | 0.048372093 | 0.021314508 | ITGB2 | 1 |
| BP | GO:1905153 | regulation of membrane invagination | 1/17 | 15/18614 | 0.01361722 | 0.048372093 | 0.021314508 | C3 | 1 |
| BP | GO:2000402 | negative regulation of lymphocyte migration | 1/17 | 15/18614 | 0.01361722 | 0.048372093 | 0.021314508 | CCL2 | 1 |
| BP | GO:0090087 | regulation of peptide transport | 2/17 | 197/18614 | 0.013652588 | 0.048372093 | 0.021314508 | CCL5/S100A8 | 2 |
| BP | GO:0001935 | endothelial cell proliferation | 2/17 | 201/18614 | 0.014183728 | 0.050066441 | 0.0220611 | CCL2/F3 | 2 |
| BP | GO:0002695 | negative regulation of leukocyte activation | 2/17 | 202/18614 | 0.014317912 | 0.050310571 | 0.022168673 | MNDA/FGL2 | 2 |
| BP | GO:0010819 | regulation of T cell chemotaxis | 1/17 | 16/18614 | 0.014518801 | 0.050310571 | 0.022168673 | CCL5 | 1 |
| BP | GO:0034349 | glial cell apoptotic process | 1/17 | 16/18614 | 0.014518801 | 0.050310571 | 0.022168673 | CCL2 | 1 |
| BP | GO:0070230 | positive regulation of lymphocyte apoptotic process | 1/17 | 16/18614 | 0.014518801 | 0.050310571 | 0.022168673 | CCL5 | 1 |
| BP | GO:2000318 | positive regulation of T-helper 17 type immune response | 1/17 | 16/18614 | 0.014518801 | 0.050310571 | 0.022168673 | CLEC7A | 1 |
| BP | GO:0002819 | regulation of adaptive immune response | 2/17 | 205/18614 | 0.014723806 | 0.050834747 | 0.022399644 | C3/CLEC7A | 2 |
| BP | GO:0051701 | biological process involved in interaction with host | 2/17 | 206/18614 | 0.014860215 | 0.051119141 | 0.022524958 | LTF/CXCR4 | 2 |
| BP | GO:0002730 | regulation of dendritic cell cytokine production | 1/17 | 17/18614 | 0.015419607 | 0.051543986 | 0.02271216 | CLEC7A | 1 |
| BP | GO:0017014 | protein nitrosylation | 1/17 | 17/18614 | 0.015419607 | 0.051543986 | 0.02271216 | S100A8 | 1 |
| BP | GO:0018119 | peptidyl-cysteine S-nitrosylation | 1/17 | 17/18614 | 0.015419607 | 0.051543986 | 0.02271216 | S100A8 | 1 |
| BP | GO:0030889 | negative regulation of B cell proliferation | 1/17 | 17/18614 | 0.015419607 | 0.051543986 | 0.02271216 | MNDA | 1 |
| BP | GO:0033631 | cell-cell adhesion mediated by integrin | 1/17 | 17/18614 | 0.015419607 | 0.051543986 | 0.02271216 | CCL5 | 1 |
| BP | GO:0034112 | positive regulation of homotypic cell-cell adhesion | 1/17 | 17/18614 | 0.015419607 | 0.051543986 | 0.02271216 | CCL5 | 1 |
| BP | GO:0090713 | immunological memory process | 1/17 | 17/18614 | 0.015419607 | 0.051543986 | 0.02271216 | FGL2 | 1 |
| BP | GO:1901163 | regulation of trophoblast cell migration | 1/17 | 17/18614 | 0.015419607 | 0.051543986 | 0.02271216 | TIMP1 | 1 |
| BP | GO:0002371 | dendritic cell cytokine production | 1/17 | 18/18614 | 0.016319637 | 0.052511486 | 0.023138476 | CLEC7A | 1 |
| BP | GO:0003159 | morphogenesis of an endothelium | 1/17 | 18/18614 | 0.016319637 | 0.052511486 | 0.023138476 | CXCR4 | 1 |
| BP | GO:0006957 | complement activation, alternative pathway | 1/17 | 18/18614 | 0.016319637 | 0.052511486 | 0.023138476 | C3 | 1 |
| BP | GO:0036035 | osteoclast development | 1/17 | 18/18614 | 0.016319637 | 0.052511486 | 0.023138476 | LTF | 1 |
| BP | GO:0043217 | myelin maintenance | 1/17 | 18/18614 | 0.016319637 | 0.052511486 | 0.023138476 | CXCR4 | 1 |
| BP | GO:0050965 | detection of temperature stimulus involved in sensory perception of pain | 1/17 | 18/18614 | 0.016319637 | 0.052511486 | 0.023138476 | CXCR4 | 1 |
| BP | GO:0051770 | positive regulation of nitric-oxide synthase biosynthetic process | 1/17 | 18/18614 | 0.016319637 | 0.052511486 | 0.023138476 | CCL2 | 1 |
| BP | GO:0060263 | regulation of respiratory burst | 1/17 | 18/18614 | 0.016319637 | 0.052511486 | 0.023138476 | CLEC7A | 1 |
| BP | GO:0061154 | endothelial tube morphogenesis | 1/17 | 18/18614 | 0.016319637 | 0.052511486 | 0.023138476 | CXCR4 | 1 |
| BP | GO:0061450 | trophoblast cell migration | 1/17 | 18/18614 | 0.016319637 | 0.052511486 | 0.023138476 | TIMP1 | 1 |
| BP | GO:0090594 | inflammatory response to wounding | 1/17 | 18/18614 | 0.016319637 | 0.052511486 | 0.023138476 | TIMP1 | 1 |
| BP | GO:0070374 | positive regulation of ERK1 and ERK2 cascade | 2/17 | 222/18614 | 0.017117387 | 0.054478505 | 0.024005216 | CCL5/CCL2 | 2 |
| BP | GO:0002283 | neutrophil activation involved in immune response | 1/17 | 19/18614 | 0.017218893 | 0.054478505 | 0.024005216 | ITGB2 | 1 |
| BP | GO:0002523 | leukocyte migration involved in inflammatory response | 1/17 | 19/18614 | 0.017218893 | 0.054478505 | 0.024005216 | S100A8 | 1 |
| BP | GO:0010759 | positive regulation of macrophage chemotaxis | 1/17 | 19/18614 | 0.017218893 | 0.054478505 | 0.024005216 | CCL5 | 1 |
| BP | GO:0098543 | detection of other organism | 1/17 | 19/18614 | 0.017218893 | 0.054478505 | 0.024005216 | CLEC7A | 1 |
| BP | GO:0050866 | negative regulation of cell activation | 2/17 | 225/18614 | 0.017556058 | 0.055360103 | 0.024393681 | MNDA/FGL2 | 2 |
| BP | GO:0002685 | regulation of leukocyte migration | 2/17 | 227/18614 | 0.017851181 | 0.056009926 | 0.024680016 | CCL5/CCL2 | 2 |
| BP | GO:0002577 | regulation of antigen processing and presentation | 1/17 | 20/18614 | 0.018117376 | 0.056009926 | 0.024680016 | FGL2 | 1 |
| BP | GO:0042053 | regulation of dopamine metabolic process | 1/17 | 20/18614 | 0.018117376 | 0.056009926 | 0.024680016 | ITGB2 | 1 |
| BP | GO:0042069 | regulation of catecholamine metabolic process | 1/17 | 20/18614 | 0.018117376 | 0.056009926 | 0.024680016 | ITGB2 | 1 |
| BP | GO:0097709 | connective tissue replacement | 1/17 | 20/18614 | 0.018117376 | 0.056009926 | 0.024680016 | TIMP1 | 1 |
| BP | GO:0140131 | positive regulation of lymphocyte chemotaxis | 1/17 | 20/18614 | 0.018117376 | 0.056009926 | 0.024680016 | CCL5 | 1 |
| BP | GO:0043270 | positive regulation of monoatomic ion transport | 2/17 | 231/18614 | 0.018447816 | 0.056845714 | 0.025048295 | CCL5/CCL2 | 2 |
| BP | GO:0007252 | I-kappaB phosphorylation | 1/17 | 21/18614 | 0.019015085 | 0.056908678 | 0.025076039 | TLR7 | 1 |
| BP | GO:0071605 | monocyte chemotactic protein-1 production | 1/17 | 21/18614 | 0.019015085 | 0.056908678 | 0.025076039 | CLEC7A | 1 |
| BP | GO:0071637 | regulation of monocyte chemotactic protein-1 production | 1/17 | 21/18614 | 0.019015085 | 0.056908678 | 0.025076039 | CLEC7A | 1 |
| BP | GO:0090026 | positive regulation of monocyte chemotaxis | 1/17 | 21/18614 | 0.019015085 | 0.056908678 | 0.025076039 | CCL5 | 1 |
| BP | GO:1903429 | regulation of cell maturation | 1/17 | 21/18614 | 0.019015085 | 0.056908678 | 0.025076039 | CLEC7A | 1 |
| BP | GO:0010001 | glial cell differentiation | 2/17 | 235/18614 | 0.019052916 | 0.056908678 | 0.025076039 | CXCR4/S100A8 | 2 |
| BP | GO:0034764 | positive regulation of transmembrane transport | 2/17 | 235/18614 | 0.019052916 | 0.056908678 | 0.025076039 | C3/CCL2 | 2 |
| BP | GO:0010466 | negative regulation of peptidase activity | 2/17 | 236/18614 | 0.019205507 | 0.056908678 | 0.025076039 | LTF/TIMP1 | 2 |
| BP | GO:0072593 | reactive oxygen species metabolic process | 2/17 | 237/18614 | 0.019358622 | 0.056908678 | 0.025076039 | CLEC7A/ITGB2 | 2 |
| BP | GO:0007160 | cell-matrix adhesion | 2/17 | 239/18614 | 0.01966642 | 0.056908678 | 0.025076039 | ACTN1/ITGB2 | 2 |
| BP | GO:0050670 | regulation of lymphocyte proliferation | 2/17 | 239/18614 | 0.01966642 | 0.056908678 | 0.025076039 | MNDA/CCL5 | 2 |
| BP | GO:0002274 | myeloid leukocyte activation | 2/17 | 240/18614 | 0.019821102 | 0.056908678 | 0.025076039 | CCL5/ITGB2 | 2 |
| BP | GO:0002223 | stimulatory C-type lectin receptor signaling pathway | 1/17 | 22/18614 | 0.019912021 | 0.056908678 | 0.025076039 | CLEC7A | 1 |
| BP | GO:0030220 | platelet formation | 1/17 | 22/18614 | 0.019912021 | 0.056908678 | 0.025076039 | ACTN1 | 1 |
| BP | GO:0030449 | regulation of complement activation | 1/17 | 22/18614 | 0.019912021 | 0.056908678 | 0.025076039 | C3 | 1 |
| BP | GO:0032780 | negative regulation of ATP-dependent activity | 1/17 | 22/18614 | 0.019912021 | 0.056908678 | 0.025076039 | LTF | 1 |
| BP | GO:0033630 | positive regulation of cell adhesion mediated by integrin | 1/17 | 22/18614 | 0.019912021 | 0.056908678 | 0.025076039 | CCL5 | 1 |
| BP | GO:0050961 | detection of temperature stimulus involved in sensory perception | 1/17 | 22/18614 | 0.019912021 | 0.056908678 | 0.025076039 | CXCR4 | 1 |
| BP | GO:0051767 | nitric-oxide synthase biosynthetic process | 1/17 | 22/18614 | 0.019912021 | 0.056908678 | 0.025076039 | CCL2 | 1 |
| BP | GO:0051769 | regulation of nitric-oxide synthase biosynthetic process | 1/17 | 22/18614 | 0.019912021 | 0.056908678 | 0.025076039 | CCL2 | 1 |
| BP | GO:1990840 | response to lectin | 1/17 | 22/18614 | 0.019912021 | 0.056908678 | 0.025076039 | CLEC7A | 1 |
| BP | GO:1990858 | cellular response to lectin | 1/17 | 22/18614 | 0.019912021 | 0.056908678 | 0.025076039 | CLEC7A | 1 |
| BP | GO:2000310 | regulation of NMDA receptor activity | 1/17 | 22/18614 | 0.019912021 | 0.056908678 | 0.025076039 | CCL2 | 1 |
| BP | GO:2001044 | regulation of integrin-mediated signaling pathway | 1/17 | 22/18614 | 0.019912021 | 0.056908678 | 0.025076039 | TIMP1 | 1 |
| BP | GO:0002790 | peptide secretion | 2/17 | 243/18614 | 0.020288266 | 0.057557147 | 0.025361778 | CCL5/S100A8 | 2 |
| BP | GO:0032944 | regulation of mononuclear cell proliferation | 2/17 | 243/18614 | 0.020288266 | 0.057557147 | 0.025361778 | MNDA/CCL5 | 2 |
| BP | GO:0002689 | negative regulation of leukocyte chemotaxis | 1/17 | 23/18614 | 0.020808186 | 0.057557147 | 0.025361778 | CCL2 | 1 |
| BP | GO:0010866 | regulation of triglyceride biosynthetic process | 1/17 | 23/18614 | 0.020808186 | 0.057557147 | 0.025361778 | C3 | 1 |
| BP | GO:0019835 | cytolysis | 1/17 | 23/18614 | 0.020808186 | 0.057557147 | 0.025361778 | LYZ | 1 |
| BP | GO:0032727 | positive regulation of interferon-alpha production | 1/17 | 23/18614 | 0.020808186 | 0.057557147 | 0.025361778 | TLR7 | 1 |
| BP | GO:0035988 | chondrocyte proliferation | 1/17 | 23/18614 | 0.020808186 | 0.057557147 | 0.025361778 | LTF | 1 |
| BP | GO:0061042 | vascular wound healing | 1/17 | 23/18614 | 0.020808186 | 0.057557147 | 0.025361778 | CXCR4 | 1 |
| BP | GO:0061760 | antifungal innate immune response | 1/17 | 23/18614 | 0.020808186 | 0.057557147 | 0.025361778 | CLEC7A | 1 |
| BP | GO:1903861 | positive regulation of dendrite extension | 1/17 | 23/18614 | 0.020808186 | 0.057557147 | 0.025361778 | CXCR4 | 1 |
| BP | GO:2000353 | positive regulation of endothelial cell apoptotic process | 1/17 | 23/18614 | 0.020808186 | 0.057557147 | 0.025361778 | CCL2 | 1 |
| BP | GO:0050870 | positive regulation of T cell activation | 2/17 | 249/18614 | 0.02123654 | 0.057998832 | 0.025556401 | CCL5/CCL2 | 2 |
| BP | GO:0016485 | protein processing | 2/17 | 250/18614 | 0.021396383 | 0.057998832 | 0.025556401 | CASP1/F3 | 2 |
| BP | GO:0002922 | positive regulation of humoral immune response | 1/17 | 24/18614 | 0.02170358 | 0.057998832 | 0.025556401 | C3 | 1 |
| BP | GO:0010640 | regulation of platelet-derived growth factor receptor signaling pathway | 1/17 | 24/18614 | 0.02170358 | 0.057998832 | 0.025556401 | F3 | 1 |
| BP | GO:0035458 | cellular response to interferon-beta | 1/17 | 24/18614 | 0.02170358 | 0.057998832 | 0.025556401 | MNDA | 1 |
| BP | GO:0036344 | platelet morphogenesis | 1/17 | 24/18614 | 0.02170358 | 0.057998832 | 0.025556401 | ACTN1 | 1 |
| BP | GO:0051043 | regulation of membrane protein ectodomain proteolysis | 1/17 | 24/18614 | 0.02170358 | 0.057998832 | 0.025556401 | TIMP1 | 1 |
| BP | GO:0060907 | positive regulation of macrophage cytokine production | 1/17 | 24/18614 | 0.02170358 | 0.057998832 | 0.025556401 | TLR7 | 1 |
| BP | GO:0070233 | negative regulation of T cell apoptotic process | 1/17 | 24/18614 | 0.02170358 | 0.057998832 | 0.025556401 | CCL5 | 1 |
| BP | GO:0071676 | negative regulation of mononuclear cell migration | 1/17 | 24/18614 | 0.02170358 | 0.057998832 | 0.025556401 | CCL2 | 1 |
| BP | GO:0098581 | detection of external biotic stimulus | 1/17 | 24/18614 | 0.02170358 | 0.057998832 | 0.025556401 | CLEC7A | 1 |
| BP | GO:0098743 | cell aggregation | 1/17 | 24/18614 | 0.02170358 | 0.057998832 | 0.025556401 | LTF | 1 |
| BP | GO:0050730 | regulation of peptidyl-tyrosine phosphorylation | 2/17 | 255/18614 | 0.02220324 | 0.059054972 | 0.026021774 | CCL5/ITGB2 | 2 |
| BP | GO:0006898 | receptor-mediated endocytosis | 2/17 | 256/18614 | 0.022366134 | 0.059054972 | 0.026021774 | C3/ITGB2 | 2 |
| BP | GO:0016048 | detection of temperature stimulus | 1/17 | 25/18614 | 0.022598203 | 0.059054972 | 0.026021774 | CXCR4 | 1 |
| BP | GO:0043302 | positive regulation of leukocyte degranulation | 1/17 | 25/18614 | 0.022598203 | 0.059054972 | 0.026021774 | ITGB2 | 1 |
| BP | GO:0048714 | positive regulation of oligodendrocyte differentiation | 1/17 | 25/18614 | 0.022598203 | 0.059054972 | 0.026021774 | CXCR4 | 1 |
| BP | GO:0050927 | positive regulation of positive chemotaxis | 1/17 | 25/18614 | 0.022598203 | 0.059054972 | 0.026021774 | F3 | 1 |
| BP | GO:1904996 | positive regulation of leukocyte adhesion to vascular endothelial cell | 1/17 | 25/18614 | 0.022598203 | 0.059054972 | 0.026021774 | ITGB2 | 1 |
| BP | GO:1905563 | negative regulation of vascular endothelial cell proliferation | 1/17 | 25/18614 | 0.022598203 | 0.059054972 | 0.026021774 | CCL2 | 1 |
| BP | GO:0045927 | positive regulation of growth | 2/17 | 258/18614 | 0.022693435 | 0.059140468 | 0.026059447 | CXCR4/S100A8 | 2 |
| BP | GO:0015833 | peptide transport | 2/17 | 259/18614 | 0.022857842 | 0.059405269 | 0.026176129 | CCL5/S100A8 | 2 |
| BP | GO:0002438 | acute inflammatory response to antigenic stimulus | 1/17 | 26/18614 | 0.023492055 | 0.059652479 | 0.026285058 | C3 | 1 |
| BP | GO:0043931 | ossification involved in bone maturation | 1/17 | 26/18614 | 0.023492055 | 0.059652479 | 0.026285058 | LTF | 1 |
| BP | GO:0050926 | regulation of positive chemotaxis | 1/17 | 26/18614 | 0.023492055 | 0.059652479 | 0.026285058 | F3 | 1 |
| BP | GO:0051238 | sequestering of metal ion | 1/17 | 26/18614 | 0.023492055 | 0.059652479 | 0.026285058 | S100A8 | 1 |
| BP | GO:1903859 | regulation of dendrite extension | 1/17 | 26/18614 | 0.023492055 | 0.059652479 | 0.026285058 | CXCR4 | 1 |
| BP | GO:1905048 | regulation of metallopeptidase activity | 1/17 | 26/18614 | 0.023492055 | 0.059652479 | 0.026285058 | TIMP1 | 1 |
| BP | GO:1905523 | positive regulation of macrophage migration | 1/17 | 26/18614 | 0.023492055 | 0.059652479 | 0.026285058 | CCL5 | 1 |
| BP | GO:0001894 | tissue homeostasis | 2/17 | 263/18614 | 0.023520481 | 0.059652479 | 0.026285058 | LYZ/LTF | 2 |
| BP | GO:0060249 | anatomical structure homeostasis | 2/17 | 263/18614 | 0.023520481 | 0.059652479 | 0.026285058 | LYZ/LTF | 2 |
| BP | GO:0070663 | regulation of leukocyte proliferation | 2/17 | 268/18614 | 0.024359992 | 0.060537901 | 0.026675208 | MNDA/CCL5 | 2 |
| BP | GO:0010818 | T cell chemotaxis | 1/17 | 27/18614 | 0.024385139 | 0.060537901 | 0.026675208 | CCL5 | 1 |
| BP | GO:0010884 | positive regulation of lipid storage | 1/17 | 27/18614 | 0.024385139 | 0.060537901 | 0.026675208 | C3 | 1 |
| BP | GO:0034143 | regulation of toll-like receptor 4 signaling pathway | 1/17 | 27/18614 | 0.024385139 | 0.060537901 | 0.026675208 | LTF | 1 |
| BP | GO:0042832 | defense response to protozoan | 1/17 | 27/18614 | 0.024385139 | 0.060537901 | 0.026675208 | CLEC7A | 1 |
| BP | GO:0045745 | positive regulation of G protein-coupled receptor signaling pathway | 1/17 | 27/18614 | 0.024385139 | 0.060537901 | 0.026675208 | C3 | 1 |
| BP | GO:0062149 | detection of stimulus involved in sensory perception of pain | 1/17 | 27/18614 | 0.024385139 | 0.060537901 | 0.026675208 | CXCR4 | 1 |
| BP | GO:2000108 | positive regulation of leukocyte apoptotic process | 1/17 | 27/18614 | 0.024385139 | 0.060537901 | 0.026675208 | CCL5 | 1 |
| BP | GO:0043547 | positive regulation of GTPase activity | 2/17 | 272/18614 | 0.025040497 | 0.060537901 | 0.026675208 | CCL5/CCL2 | 2 |
| BP | GO:0002227 | innate immune response in mucosa | 1/17 | 28/18614 | 0.025277454 | 0.060537901 | 0.026675208 | LTF | 1 |
| BP | GO:0002675 | positive regulation of acute inflammatory response | 1/17 | 28/18614 | 0.025277454 | 0.060537901 | 0.026675208 | C3 | 1 |
| BP | GO:0010758 | regulation of macrophage chemotaxis | 1/17 | 28/18614 | 0.025277454 | 0.060537901 | 0.026675208 | CCL5 | 1 |
| BP | GO:0014072 | response to isoquinoline alkaloid | 1/17 | 28/18614 | 0.025277454 | 0.060537901 | 0.026675208 | CXCR4 | 1 |
| BP | GO:0016540 | protein autoprocessing | 1/17 | 28/18614 | 0.025277454 | 0.060537901 | 0.026675208 | CASP1 | 1 |
| BP | GO:0030194 | positive regulation of blood coagulation | 1/17 | 28/18614 | 0.025277454 | 0.060537901 | 0.026675208 | F3 | 1 |
| BP | GO:0031664 | regulation of lipopolysaccharide-mediated signaling pathway | 1/17 | 28/18614 | 0.025277454 | 0.060537901 | 0.026675208 | LTF | 1 |
| BP | GO:0043278 | response to morphine | 1/17 | 28/18614 | 0.025277454 | 0.060537901 | 0.026675208 | CXCR4 | 1 |
| BP | GO:0050951 | sensory perception of temperature stimulus | 1/17 | 28/18614 | 0.025277454 | 0.060537901 | 0.026675208 | CXCR4 | 1 |
| BP | GO:0070269 | pyroptosis | 1/17 | 28/18614 | 0.025277454 | 0.060537901 | 0.026675208 | CASP1 | 1 |
| BP | GO:0090314 | positive regulation of protein targeting to membrane | 1/17 | 28/18614 | 0.025277454 | 0.060537901 | 0.026675208 | ITGB2 | 1 |
| BP | GO:0150146 | cell junction disassembly | 1/17 | 28/18614 | 0.025277454 | 0.060537901 | 0.026675208 | C3 | 1 |
| BP | GO:1900048 | positive regulation of hemostasis | 1/17 | 28/18614 | 0.025277454 | 0.060537901 | 0.026675208 | F3 | 1 |
| BP | GO:0001562 | response to protozoan | 1/17 | 29/18614 | 0.026169 | 0.061428968 | 0.027067844 | CLEC7A | 1 |
| BP | GO:0006910 | phagocytosis, recognition | 1/17 | 29/18614 | 0.026169 | 0.061428968 | 0.027067844 | CLEC7A | 1 |
| BP | GO:0010575 | positive regulation of vascular endothelial growth factor production | 1/17 | 29/18614 | 0.026169 | 0.061428968 | 0.027067844 | C3 | 1 |
| BP | GO:0032607 | interferon-alpha production | 1/17 | 29/18614 | 0.026169 | 0.061428968 | 0.027067844 | TLR7 | 1 |
| BP | GO:0032647 | regulation of interferon-alpha production | 1/17 | 29/18614 | 0.026169 | 0.061428968 | 0.027067844 | TLR7 | 1 |
| BP | GO:0070977 | bone maturation | 1/17 | 29/18614 | 0.026169 | 0.061428968 | 0.027067844 | LTF | 1 |
| BP | GO:0090025 | regulation of monocyte chemotaxis | 1/17 | 29/18614 | 0.026169 | 0.061428968 | 0.027067844 | CCL5 | 1 |
| BP | GO:1900017 | positive regulation of cytokine production involved in inflammatory response | 1/17 | 29/18614 | 0.026169 | 0.061428968 | 0.027067844 | CLEC7A | 1 |
| BP | GO:0006882 | intracellular zinc ion homeostasis | 1/17 | 30/18614 | 0.027059779 | 0.063206299 | 0.027851001 | S100A8 | 1 |
| BP | GO:0050820 | positive regulation of coagulation | 1/17 | 30/18614 | 0.027059779 | 0.063206299 | 0.027851001 | F3 | 1 |
| BP | GO:0010721 | negative regulation of cell development | 2/17 | 286/18614 | 0.027483651 | 0.064038261 | 0.028217593 | LTF/FGL2 | 2 |
| BP | GO:0033688 | regulation of osteoblast proliferation | 1/17 | 31/18614 | 0.027949791 | 0.064646705 | 0.028485696 | LTF | 1 |
| BP | GO:0045948 | positive regulation of translational initiation | 1/17 | 31/18614 | 0.027949791 | 0.064646705 | 0.028485696 | CCL5 | 1 |
| BP | GO:2000316 | regulation of T-helper 17 type immune response | 1/17 | 31/18614 | 0.027949791 | 0.064646705 | 0.028485696 | CLEC7A | 1 |
| BP | GO:0019932 | second-messenger-mediated signaling | 2/17 | 292/18614 | 0.028559508 | 0.065895841 | 0.029036111 | CLEC7A/CXCR4 | 2 |
| BP | GO:0035456 | response to interferon-beta | 1/17 | 32/18614 | 0.028839037 | 0.065897896 | 0.029037016 | MNDA | 1 |
| BP | GO:0045070 | positive regulation of viral genome replication | 1/17 | 32/18614 | 0.028839037 | 0.065897896 | 0.029037016 | CCL5 | 1 |
| BP | GO:0045671 | negative regulation of osteoclast differentiation | 1/17 | 32/18614 | 0.028839037 | 0.065897896 | 0.029037016 | LTF | 1 |
| BP | GO:0060055 | angiogenesis involved in wound healing | 1/17 | 32/18614 | 0.028839037 | 0.065897896 | 0.029037016 | CXCR4 | 1 |
| BP | GO:0002366 | leukocyte activation involved in immune response | 2/17 | 296/18614 | 0.029286198 | 0.066758417 | 0.029416194 | FGL2/ITGB2 | 2 |
| BP | GO:0006958 | complement activation, classical pathway | 1/17 | 33/18614 | 0.029727517 | 0.067117496 | 0.029574417 | C3 | 1 |
| BP | GO:0048799 | animal organ maturation | 1/17 | 33/18614 | 0.029727517 | 0.067117496 | 0.029574417 | LTF | 1 |
| BP | GO:0071404 | cellular response to low-density lipoprotein particle stimulus | 1/17 | 33/18614 | 0.029727517 | 0.067117496 | 0.029574417 | ITGB2 | 1 |
| BP | GO:0090279 | regulation of calcium ion import | 1/17 | 33/18614 | 0.029727517 | 0.067117496 | 0.029574417 | CCL2 | 1 |
| BP | GO:0002263 | cell activation involved in immune response | 2/17 | 300/18614 | 0.030020382 | 0.067617336 | 0.029794664 | FGL2/ITGB2 | 2 |
| BP | GO:0032743 | positive regulation of interleukin-2 production | 1/17 | 34/18614 | 0.030615232 | 0.067985937 | 0.029957084 | CLEC7A | 1 |
| BP | GO:0033238 | regulation of amine metabolic process | 1/17 | 34/18614 | 0.030615232 | 0.067985937 | 0.029957084 | ITGB2 | 1 |
| BP | GO:0050869 | negative regulation of B cell activation | 1/17 | 34/18614 | 0.030615232 | 0.067985937 | 0.029957084 | MNDA | 1 |
| BP | GO:0055094 | response to lipoprotein particle | 1/17 | 34/18614 | 0.030615232 | 0.067985937 | 0.029957084 | ITGB2 | 1 |
| BP | GO:0090313 | regulation of protein targeting to membrane | 1/17 | 34/18614 | 0.030615232 | 0.067985937 | 0.029957084 | ITGB2 | 1 |
| BP | GO:2000406 | positive regulation of T cell migration | 1/17 | 34/18614 | 0.030615232 | 0.067985937 | 0.029957084 | CCL5 | 1 |
| BP | GO:0046651 | lymphocyte proliferation | 2/17 | 306/18614 | 0.031135583 | 0.068979536 | 0.0303949 | MNDA/CCL5 | 2 |
| BP | GO:0002446 | neutrophil mediated immunity | 1/17 | 35/18614 | 0.031502182 | 0.069144001 | 0.030467369 | ITGB2 | 1 |
| BP | GO:0050974 | detection of mechanical stimulus involved in sensory perception | 1/17 | 35/18614 | 0.031502182 | 0.069144001 | 0.030467369 | CXCR4 | 1 |
| BP | GO:0051968 | positive regulation of synaptic transmission, glutamatergic | 1/17 | 35/18614 | 0.031502182 | 0.069144001 | 0.030467369 | CCL2 | 1 |
| BP | GO:0061081 | positive regulation of myeloid leukocyte cytokine production involved in immune response | 1/17 | 35/18614 | 0.031502182 | 0.069144001 | 0.030467369 | TLR7 | 1 |
| BP | GO:0002460 | adaptive immune response based on somatic recombination of immune receptors built from immunoglobulin superfamily domains | 2/17 | 311/18614 | 0.032077561 | 0.070243919 | 0.030952033 | C3/CLEC7A | 2 |
| BP | GO:0033687 | osteoblast proliferation | 1/17 | 36/18614 | 0.032388369 | 0.070423937 | 0.031031355 | LTF | 1 |
| BP | GO:0050687 | negative regulation of defense response to virus | 1/17 | 36/18614 | 0.032388369 | 0.070423937 | 0.031031355 | FGL2 | 1 |
| BP | GO:0097484 | dendrite extension | 1/17 | 36/18614 | 0.032388369 | 0.070423937 | 0.031031355 | CXCR4 | 1 |
| BP | GO:0032943 | mononuclear cell proliferation | 2/17 | 313/18614 | 0.032457544 | 0.070423937 | 0.031031355 | MNDA/CCL5 | 2 |
| BP | GO:0070372 | regulation of ERK1 and ERK2 cascade | 2/17 | 315/18614 | 0.032839339 | 0.071089279 | 0.031324529 | CCL5/CCL2 | 2 |
| BP | GO:0051047 | positive regulation of secretion | 2/17 | 317/18614 | 0.033222941 | 0.07121495 | 0.031379904 | S100A8/ITGB2 | 2 |
| BP | GO:0010934 | macrophage cytokine production | 1/17 | 37/18614 | 0.033273793 | 0.07121495 | 0.031379904 | TLR7 | 1 |
| BP | GO:0010935 | regulation of macrophage cytokine production | 1/17 | 37/18614 | 0.033273793 | 0.07121495 | 0.031379904 | TLR7 | 1 |
| BP | GO:0034110 | regulation of homotypic cell-cell adhesion | 1/17 | 37/18614 | 0.033273793 | 0.07121495 | 0.031379904 | CCL5 | 1 |
| BP | GO:0071402 | cellular response to lipoprotein particle stimulus | 1/17 | 37/18614 | 0.033273793 | 0.07121495 | 0.031379904 | ITGB2 | 1 |
| BP | GO:1902105 | regulation of leukocyte differentiation | 2/17 | 319/18614 | 0.033608342 | 0.071768603 | 0.031623864 | LTF/FGL2 | 2 |
| BP | GO:0009595 | detection of biotic stimulus | 1/17 | 38/18614 | 0.034158454 | 0.072290597 | 0.031853874 | CLEC7A | 1 |
| BP | GO:0032728 | positive regulation of interferon-beta production | 1/17 | 38/18614 | 0.034158454 | 0.072290597 | 0.031853874 | TLR7 | 1 |
| BP | GO:0070229 | negative regulation of lymphocyte apoptotic process | 1/17 | 38/18614 | 0.034158454 | 0.072290597 | 0.031853874 | CCL5 | 1 |
| BP | GO:0140894 | endolysosomal toll-like receptor signaling pathway | 1/17 | 38/18614 | 0.034158454 | 0.072290597 | 0.031853874 | TLR7 | 1 |
| BP | GO:0002385 | mucosal immune response | 1/17 | 39/18614 | 0.035042352 | 0.073666812 | 0.032460284 | LTF | 1 |
| BP | GO:0045730 | respiratory burst | 1/17 | 39/18614 | 0.035042352 | 0.073666812 | 0.032460284 | CLEC7A | 1 |
| BP | GO:0070232 | regulation of T cell apoptotic process | 1/17 | 39/18614 | 0.035042352 | 0.073666812 | 0.032460284 | CCL5 | 1 |
| BP | GO:0071496 | cellular response to external stimulus | 2/17 | 327/18614 | 0.035167795 | 0.073766594 | 0.032504252 | CASP1/TLR7 | 2 |
| BP | GO:0002440 | production of molecular mediator of immune response | 2/17 | 328/18614 | 0.03536472 | 0.074015543 | 0.032613948 | TLR7/CLEC7A | 2 |
| BP | GO:0045861 | negative regulation of proteolysis | 2/17 | 330/18614 | 0.035759891 | 0.07436655 | 0.032768615 | LTF/TIMP1 | 2 |
| BP | GO:0050850 | positive regulation of calcium-mediated signaling | 1/17 | 40/18614 | 0.03592549 | 0.07436655 | 0.032768615 | CLEC7A | 1 |
| BP | GO:0070884 | regulation of calcineurin-NFAT signaling cascade | 1/17 | 40/18614 | 0.03592549 | 0.07436655 | 0.032768615 | CLEC7A | 1 |
| BP | GO:0140448 | signaling receptor ligand precursor processing | 1/17 | 40/18614 | 0.03592549 | 0.07436655 | 0.032768615 | CASP1 | 1 |
| BP | GO:1904994 | regulation of leukocyte adhesion to vascular endothelial cell | 1/17 | 40/18614 | 0.03592549 | 0.07436655 | 0.032768615 | ITGB2 | 1 |
| BP | GO:0051604 | protein maturation | 2/17 | 332/18614 | 0.036156816 | 0.074681982 | 0.032907605 | CASP1/F3 | 2 |
| BP | GO:0019432 | triglyceride biosynthetic process | 1/17 | 41/18614 | 0.036807867 | 0.075205706 | 0.033138377 | C3 | 1 |
| BP | GO:0042417 | dopamine metabolic process | 1/17 | 41/18614 | 0.036807867 | 0.075205706 | 0.033138377 | ITGB2 | 1 |
| BP | GO:0098751 | bone cell development | 1/17 | 41/18614 | 0.036807867 | 0.075205706 | 0.033138377 | LTF | 1 |
| BP | GO:0106056 | regulation of calcineurin-mediated signaling | 1/17 | 41/18614 | 0.036807867 | 0.075205706 | 0.033138377 | CLEC7A | 1 |
| BP | GO:2000403 | positive regulation of lymphocyte migration | 1/17 | 41/18614 | 0.036807867 | 0.075205706 | 0.033138377 | CCL5 | 1 |
| BP | GO:0002714 | positive regulation of B cell mediated immunity | 1/17 | 42/18614 | 0.037689483 | 0.076021858 | 0.033498003 | C3 | 1 |
| BP | GO:0002891 | positive regulation of immunoglobulin mediated immune response | 1/17 | 42/18614 | 0.037689483 | 0.076021858 | 0.033498003 | C3 | 1 |
| BP | GO:0032733 | positive regulation of interleukin-10 production | 1/17 | 42/18614 | 0.037689483 | 0.076021858 | 0.033498003 | CLEC7A | 1 |
| BP | GO:0045429 | positive regulation of nitric oxide biosynthetic process | 1/17 | 42/18614 | 0.037689483 | 0.076021858 | 0.033498003 | CLEC7A | 1 |
| BP | GO:0090207 | regulation of triglyceride metabolic process | 1/17 | 42/18614 | 0.037689483 | 0.076021858 | 0.033498003 | C3 | 1 |
| BP | GO:1904037 | positive regulation of epithelial cell apoptotic process | 1/17 | 42/18614 | 0.037689483 | 0.076021858 | 0.033498003 | CCL2 | 1 |
| BP | GO:0070371 | ERK1 and ERK2 cascade | 2/17 | 341/18614 | 0.037964496 | 0.076413644 | 0.033670639 | CCL5/CCL2 | 2 |
| BP | GO:0002251 | organ or tissue specific immune response | 1/17 | 43/18614 | 0.038570341 | 0.076493799 | 0.033705958 | LTF | 1 |
| BP | GO:0014002 | astrocyte development | 1/17 | 43/18614 | 0.038570341 | 0.076493799 | 0.033705958 | S100A8 | 1 |
| BP | GO:0030501 | positive regulation of bone mineralization | 1/17 | 43/18614 | 0.038570341 | 0.076493799 | 0.033705958 | LTF | 1 |
| BP | GO:0032735 | positive regulation of interleukin-12 production | 1/17 | 43/18614 | 0.038570341 | 0.076493799 | 0.033705958 | CLEC7A | 1 |
| BP | GO:0034142 | toll-like receptor 4 signaling pathway | 1/17 | 43/18614 | 0.038570341 | 0.076493799 | 0.033705958 | LTF | 1 |
| BP | GO:0045687 | positive regulation of glial cell differentiation | 1/17 | 43/18614 | 0.038570341 | 0.076493799 | 0.033705958 | CXCR4 | 1 |
| BP | GO:0048713 | regulation of oligodendrocyte differentiation | 1/17 | 43/18614 | 0.038570341 | 0.076493799 | 0.033705958 | CXCR4 | 1 |
| BP | GO:0051346 | negative regulation of hydrolase activity | 2/17 | 345/18614 | 0.038779101 | 0.076746924 | 0.033817494 | LTF/TIMP1 | 2 |
| BP | GO:0051235 | maintenance of location | 2/17 | 347/18614 | 0.039188961 | 0.076948691 | 0.0339064 | C3/S100A8 | 2 |
| BP | GO:0070661 | leukocyte proliferation | 2/17 | 348/18614 | 0.039394527 | 0.076948691 | 0.0339064 | MNDA/CCL5 | 2 |
| BP | GO:0006509 | membrane protein ectodomain proteolysis | 1/17 | 44/18614 | 0.039450439 | 0.076948691 | 0.0339064 | TIMP1 | 1 |
| BP | GO:0010828 | positive regulation of glucose transmembrane transport | 1/17 | 44/18614 | 0.039450439 | 0.076948691 | 0.0339064 | C3 | 1 |
| BP | GO:0072538 | T-helper 17 type immune response | 1/17 | 44/18614 | 0.039450439 | 0.076948691 | 0.0339064 | CLEC7A | 1 |
| BP | GO:1904407 | positive regulation of nitric oxide metabolic process | 1/17 | 44/18614 | 0.039450439 | 0.076948691 | 0.0339064 | CLEC7A | 1 |
| BP | GO:1905521 | regulation of macrophage migration | 1/17 | 44/18614 | 0.039450439 | 0.076948691 | 0.0339064 | CCL5 | 1 |
| BP | GO:0042886 | amide transport | 2/17 | 349/18614 | 0.039600517 | 0.077082487 | 0.033965355 | CCL5/S100A8 | 2 |
| BP | GO:0006887 | exocytosis | 2/17 | 351/18614 | 0.040013762 | 0.077726937 | 0.034249324 | CCL5/ITGB2 | 2 |
| BP | GO:0033173 | calcineurin-NFAT signaling cascade | 1/17 | 45/18614 | 0.040329779 | 0.077861165 | 0.034308469 | CLEC7A | 1 |
| BP | GO:0062208 | positive regulation of pattern recognition receptor signaling pathway | 1/17 | 45/18614 | 0.040329779 | 0.077861165 | 0.034308469 | LTF | 1 |
| BP | GO:1903307 | positive regulation of regulated secretory pathway | 1/17 | 45/18614 | 0.040329779 | 0.077861165 | 0.034308469 | ITGB2 | 1 |
| BP | GO:0002455 | humoral immune response mediated by circulating immunoglobulin | 1/17 | 46/18614 | 0.041208361 | 0.07843684 | 0.034562133 | C3 | 1 |
| BP | GO:0008038 | neuron recognition | 1/17 | 46/18614 | 0.041208361 | 0.07843684 | 0.034562133 | CXCR4 | 1 |
| BP | GO:0014911 | positive regulation of smooth muscle cell migration | 1/17 | 46/18614 | 0.041208361 | 0.07843684 | 0.034562133 | CCL5 | 1 |
| BP | GO:0042551 | neuron maturation | 1/17 | 46/18614 | 0.041208361 | 0.07843684 | 0.034562133 | C3 | 1 |
| BP | GO:0044788 | modulation by host of viral process | 1/17 | 46/18614 | 0.041208361 | 0.07843684 | 0.034562133 | LTF | 1 |
| BP | GO:0046427 | positive regulation of receptor signaling pathway via JAK-STAT | 1/17 | 46/18614 | 0.041208361 | 0.07843684 | 0.034562133 | CCL5 | 1 |
| BP | GO:2000273 | positive regulation of signaling receptor activity | 1/17 | 46/18614 | 0.041208361 | 0.07843684 | 0.034562133 | CCL2 | 1 |
| BP | GO:0031589 | cell-substrate adhesion | 2/17 | 359/18614 | 0.041683498 | 0.079181905 | 0.034890435 | ACTN1/ITGB2 | 2 |
| BP | GO:0001774 | microglial cell activation | 1/17 | 47/18614 | 0.042086186 | 0.079468128 | 0.035016556 | ITGB2 | 1 |
| BP | GO:0002861 | regulation of inflammatory response to antigenic stimulus | 1/17 | 47/18614 | 0.042086186 | 0.079468128 | 0.035016556 | C3 | 1 |
| BP | GO:0002920 | regulation of humoral immune response | 1/17 | 47/18614 | 0.042086186 | 0.079468128 | 0.035016556 | C3 | 1 |
| BP | GO:0043087 | regulation of GTPase activity | 2/17 | 364/18614 | 0.042740561 | 0.08016418 | 0.035323262 | CCL5/CCL2 | 2 |
| BP | GO:0002673 | regulation of acute inflammatory response | 1/17 | 48/18614 | 0.042963255 | 0.08016418 | 0.035323262 | C3 | 1 |
| BP | GO:0035987 | endodermal cell differentiation | 1/17 | 48/18614 | 0.042963255 | 0.08016418 | 0.035323262 | ITGB2 | 1 |
| BP | GO:0043300 | regulation of leukocyte degranulation | 1/17 | 48/18614 | 0.042963255 | 0.08016418 | 0.035323262 | ITGB2 | 1 |
| BP | GO:0070509 | calcium ion import | 1/17 | 48/18614 | 0.042963255 | 0.08016418 | 0.035323262 | CCL2 | 1 |
| BP | GO:2000404 | regulation of T cell migration | 1/17 | 48/18614 | 0.042963255 | 0.08016418 | 0.035323262 | CCL5 | 1 |
| BP | GO:0045860 | positive regulation of protein kinase activity | 2/17 | 366/18614 | 0.043166261 | 0.080384415 | 0.035420305 | LTF/CCL5 | 2 |
| BP | GO:0010043 | response to zinc ion | 1/17 | 49/18614 | 0.043839568 | 0.080528604 | 0.03548384 | S100A8 | 1 |
| BP | GO:0033628 | regulation of cell adhesion mediated by integrin | 1/17 | 49/18614 | 0.043839568 | 0.080528604 | 0.03548384 | CCL5 | 1 |
| BP | GO:0046460 | neutral lipid biosynthetic process | 1/17 | 49/18614 | 0.043839568 | 0.080528604 | 0.03548384 | C3 | 1 |
| BP | GO:0046463 | acylglycerol biosynthetic process | 1/17 | 49/18614 | 0.043839568 | 0.080528604 | 0.03548384 | C3 | 1 |
| BP | GO:0071470 | cellular response to osmotic stress | 1/17 | 49/18614 | 0.043839568 | 0.080528604 | 0.03548384 | CASP1 | 1 |
| BP | GO:0097720 | calcineurin-mediated signaling | 1/17 | 49/18614 | 0.043839568 | 0.080528604 | 0.03548384 | CLEC7A | 1 |
| BP | GO:0140895 | cell surface toll-like receptor signaling pathway | 1/17 | 49/18614 | 0.043839568 | 0.080528604 | 0.03548384 | LTF | 1 |
| BP | GO:0018108 | peptidyl-tyrosine phosphorylation | 2/17 | 370/18614 | 0.044022551 | 0.08070801 | 0.035562893 | CCL5/ITGB2 | 2 |
| BP | GO:0018212 | peptidyl-tyrosine modification | 2/17 | 372/18614 | 0.044453128 | 0.081190995 | 0.035775714 | CCL5/ITGB2 | 2 |
| BP | GO:0002269 | leukocyte activation involved in inflammatory response | 1/17 | 50/18614 | 0.044715125 | 0.081190995 | 0.035775714 | ITGB2 | 1 |
| BP | GO:0018198 | peptidyl-cysteine modification | 1/17 | 50/18614 | 0.044715125 | 0.081190995 | 0.035775714 | S100A8 | 1 |
| BP | GO:0045581 | negative regulation of T cell differentiation | 1/17 | 50/18614 | 0.044715125 | 0.081190995 | 0.035775714 | FGL2 | 1 |
| BP | GO:1904894 | positive regulation of receptor signaling pathway via STAT | 1/17 | 50/18614 | 0.044715125 | 0.081190995 | 0.035775714 | CCL5 | 1 |
| BP | GO:0030336 | negative regulation of cell migration | 2/17 | 375/18614 | 0.045102015 | 0.081736602 | 0.036016128 | TIMP1/CCL2 | 2 |
| BP | GO:0019083 | viral transcription | 1/17 | 51/18614 | 0.045589928 | 0.081992533 | 0.036128901 | CCL5 | 1 |
| BP | GO:0043113 | receptor clustering | 1/17 | 51/18614 | 0.045589928 | 0.081992533 | 0.036128901 | ITGB2 | 1 |
| BP | GO:0045778 | positive regulation of ossification | 1/17 | 51/18614 | 0.045589928 | 0.081992533 | 0.036128901 | LTF | 1 |
| BP | GO:0061082 | myeloid leukocyte cytokine production | 1/17 | 51/18614 | 0.045589928 | 0.081992533 | 0.036128901 | TLR7 | 1 |
| BP | GO:0002762 | negative regulation of myeloid leukocyte differentiation | 1/17 | 52/18614 | 0.046463977 | 0.082933816 | 0.036543664 | LTF | 1 |
| BP | GO:0006968 | cellular defense response | 1/17 | 52/18614 | 0.046463977 | 0.082933816 | 0.036543664 | MNDA | 1 |
| BP | GO:0101023 | vascular endothelial cell proliferation | 1/17 | 52/18614 | 0.046463977 | 0.082933816 | 0.036543664 | CCL2 | 1 |
| BP | GO:1905562 | regulation of vascular endothelial cell proliferation | 1/17 | 52/18614 | 0.046463977 | 0.082933816 | 0.036543664 | CCL2 | 1 |
| BP | GO:0010883 | regulation of lipid storage | 1/17 | 53/18614 | 0.047337273 | 0.083546754 | 0.036813747 | C3 | 1 |
| BP | GO:0045744 | negative regulation of G protein-coupled receptor signaling pathway | 1/17 | 53/18614 | 0.047337273 | 0.083546754 | 0.036813747 | CCL5 | 1 |
| BP | GO:0048260 | positive regulation of receptor-mediated endocytosis | 1/17 | 53/18614 | 0.047337273 | 0.083546754 | 0.036813747 | C3 | 1 |
| BP | GO:0050982 | detection of mechanical stimulus | 1/17 | 53/18614 | 0.047337273 | 0.083546754 | 0.036813747 | CXCR4 | 1 |
| BP | GO:0070169 | positive regulation of biomineral tissue development | 1/17 | 53/18614 | 0.047337273 | 0.083546754 | 0.036813747 | LTF | 1 |
| BP | GO:0071622 | regulation of granulocyte chemotaxis | 1/17 | 53/18614 | 0.047337273 | 0.083546754 | 0.036813747 | CCL5 | 1 |
| BP | GO:2000146 | negative regulation of cell motility | 2/17 | 390/18614 | 0.048400109 | 0.085263506 | 0.037570211 | TIMP1/CCL2 | 2 |
| BP | GO:0002686 | negative regulation of leukocyte migration | 1/17 | 55/18614 | 0.049081606 | 0.085508655 | 0.037678232 | CCL2 | 1 |
| BP | GO:0006584 | catecholamine metabolic process | 1/17 | 55/18614 | 0.049081606 | 0.085508655 | 0.037678232 | ITGB2 | 1 |
| BP | GO:0009712 | catechol-containing compound metabolic process | 1/17 | 55/18614 | 0.049081606 | 0.085508655 | 0.037678232 | ITGB2 | 1 |
| BP | GO:0010518 | positive regulation of phospholipase activity | 1/17 | 55/18614 | 0.049081606 | 0.085508655 | 0.037678232 | CCL5 | 1 |
| BP | GO:0051703 | biological process involved in intraspecies interaction between organisms | 1/17 | 55/18614 | 0.049081606 | 0.085508655 | 0.037678232 | LTF | 1 |
| BP | GO:0061900 | glial cell activation | 1/17 | 55/18614 | 0.049081606 | 0.085508655 | 0.037678232 | ITGB2 | 1 |
| BP | GO:0002752 | cell surface pattern recognition receptor signaling pathway | 1/17 | 56/18614 | 0.049952645 | 0.08591855 | 0.037858847 | LTF | 1 |
| BP | GO:0007187 | G protein-coupled receptor signaling pathway, coupled to cyclic nucleotide second messenger | 1/17 | 56/18614 | 0.049952645 | 0.08591855 | 0.037858847 | CCL2 | 1 |
| BP | GO:0010803 | regulation of tumor necrosis factor-mediated signaling pathway | 1/17 | 56/18614 | 0.049952645 | 0.08591855 | 0.037858847 | CASP1 | 1 |
| BP | GO:0032608 | interferon-beta production | 1/17 | 56/18614 | 0.049952645 | 0.08591855 | 0.037858847 | TLR7 | 1 |
| BP | GO:0032648 | regulation of interferon-beta production | 1/17 | 56/18614 | 0.049952645 | 0.08591855 | 0.037858847 | TLR7 | 1 |
| BP | GO:0070231 | T cell apoptotic process | 1/17 | 56/18614 | 0.049952645 | 0.08591855 | 0.037858847 | CCL5 | 1 |
| BP | GO:2000107 | negative regulation of leukocyte apoptotic process | 1/17 | 56/18614 | 0.049952645 | 0.08591855 | 0.037858847 | CCL5 | 1 |
| CC | GO:0034774 | secretory granule lumen | 8/17 | 322/19518 | 1.08E-10 | 3.00E-09 | 1.88E-09 | LYZ/MNDA/C3/LTF/TIMP1/CXCL1/ACTN1/S100A8 | 8 |
| CC | GO:0060205 | cytoplasmic vesicle lumen | 8/17 | 325/19518 | 1.16E-10 | 3.00E-09 | 1.88E-09 | LYZ/MNDA/C3/LTF/TIMP1/CXCL1/ACTN1/S100A8 | 8 |
| CC | GO:0031983 | vesicle lumen | 8/17 | 327/19518 | 1.22E-10 | 3.00E-09 | 1.88E-09 | LYZ/MNDA/C3/LTF/TIMP1/CXCL1/ACTN1/S100A8 | 8 |
| CC | GO:0042581 | specific granule | 4/17 | 160/19518 | 9.53E-06 | 0.000155418 | 9.73E-05 | LYZ/LTF/CXCL1/ITGB2 | 4 |
| CC | GO:0070820 | tertiary granule | 4/17 | 164/19518 | 1.05E-05 | 0.000155418 | 9.73E-05 | LYZ/LTF/CXCL1/ITGB2 | 4 |
| CC | GO:1904724 | tertiary granule lumen | 3/17 | 55/19518 | 1.40E-05 | 0.000172674 | 0.000108075 | LYZ/LTF/CXCL1 | 3 |
| CC | GO:0035580 | specific granule lumen | 3/17 | 62/19518 | 2.01E-05 | 0.000212561 | 0.00013304 | LYZ/LTF/CXCL1 | 3 |
| CC | GO:0035578 | azurophil granule lumen | 3/17 | 91/19518 | 6.36E-05 | 0.000588183 | 0.000368137 | LYZ/MNDA/C3 | 3 |
| CC | GO:0005766 | primary lysosome | 3/17 | 155/19518 | 0.000307803 | 0.002277745 | 0.001425616 | LYZ/MNDA/C3 | 3 |
| CC | GO:0042582 | azurophil granule | 3/17 | 155/19518 | 0.000307803 | 0.002277745 | 0.001425616 | LYZ/MNDA/C3 | 3 |
| CC | GO:0062023 | collagen-containing extracellular matrix | 4/17 | 415/19518 | 0.000384876 | 0.002589167 | 0.001620531 | TIMP1/FGL2/S100A8/F3 | 4 |
| CC | GO:0005775 | vacuolar lumen | 3/17 | 176/19518 | 0.000446599 | 0.002754026 | 0.001723715 | LYZ/MNDA/C3 | 3 |
| CC | GO:0101002 | ficolin-1-rich granule | 3/17 | 185/19518 | 0.000516598 | 0.002940637 | 0.001840512 | MNDA/FGL2/ITGB2 | 3 |
| CC | GO:0031093 | platelet alpha granule lumen | 2/17 | 67/19518 | 0.001527049 | 0.008071545 | 0.005051892 | TIMP1/ACTN1 | 2 |
| CC | GO:0031091 | platelet alpha granule | 2/17 | 91/19518 | 0.002793745 | 0.013782474 | 0.0086263 | TIMP1/ACTN1 | 2 |
| CC | GO:1904813 | ficolin-1-rich granule lumen | 2/17 | 124/19518 | 0.005115694 | 0.023660086 | 0.014808589 | MNDA/FGL2 | 2 |
| CC | GO:0045335 | phagocytic vesicle | 2/17 | 141/19518 | 0.00656378 | 0.028571749 | 0.017882744 | LTF/TLR7 | 2 |
| CC | GO:1904090 | peptidase inhibitor complex | 1/17 | 11/19518 | 0.009541718 | 0.039227063 | 0.024551789 | CASP1 | 1 |
| CC | GO:0032009 | early phagosome | 1/17 | 13/19518 | 0.011267343 | 0.043883337 | 0.0274661 | TLR7 | 1 |
| CC | GO:0061702 | inflammasome complex | 1/17 | 16/19518 | 0.013850476 | 0.051246762 | 0.032074787 | CASP1 | 1 |
| CC | GO:0031143 | pseudopodium | 1/17 | 18/19518 | 0.015569035 | 0.052368572 | 0.032776915 | ACTN1 | 1 |
| CC | GO:1905286 | serine-type peptidase complex | 1/17 | 18/19518 | 0.015569035 | 0.052368572 | 0.032776915 | F3 | 1 |
| CC | GO:0036020 | endolysosome membrane | 1/17 | 20/19518 | 0.017284774 | 0.055611883 | 0.034806868 | TLR7 | 1 |
| CC | GO:0071682 | endocytic vesicle lumen | 1/17 | 23/19518 | 0.019853107 | 0.061213748 | 0.038313014 | LTF | 1 |
| CC | GO:0036019 | endolysosome | 1/17 | 29/19518 | 0.024970833 | 0.073913665 | 0.046261753 | TLR7 | 1 |
| CC | GO:0008305 | integrin complex | 1/17 | 31/19518 | 0.026671145 | 0.075910181 | 0.047511351 | ITGB2 | 1 |
| CC | GO:0005788 | endoplasmic reticulum lumen | 2/17 | 312/19518 | 0.029572855 | 0.081051529 | 0.050729264 | C3/TIMP1 | 2 |
| CC | GO:0030139 | endocytic vesicle | 2/17 | 347/19518 | 0.035947197 | 0.095003306 | 0.059461529 | LTF/TLR7 | 2 |
| CC | GO:0014704 | intercalated disc | 1/17 | 50/19518 | 0.042685516 | 0.10892166 | 0.068172874 | ACTN1 | 1 |
| CC | GO:0098636 | protein complex involved in cell adhesion | 1/17 | 57/19518 | 0.048522781 | 0.109758846 | 0.068696859 | ITGB2 | 1 |
| MF | GO:0008009 | chemokine activity | 3/17 | 49/18369 | 1.18E-05 | 0.001276016 | 0.00054722 | CCL5/CCL2/CXCL1 | 3 |
| MF | GO:0042379 | chemokine receptor binding | 3/17 | 74/18369 | 4.10E-05 | 0.001471974 | 0.000631256 | CCL5/CCL2/CXCL1 | 3 |
| MF | GO:0061134 | peptidase regulator activity | 4/17 | 230/18369 | 5.01E-05 | 0.001471974 | 0.000631256 | CASP1/C3/LTF/TIMP1 | 4 |
| MF | GO:0005125 | cytokine activity | 4/17 | 235/18369 | 5.45E-05 | 0.001471974 | 0.000631256 | TIMP1/CCL5/CCL2/CXCL1 | 4 |
| MF | GO:0001664 | G protein-coupled receptor binding | 4/17 | 289/18369 | 0.000121504 | 0.002624483 | 0.001125509 | C3/CCL5/CCL2/CXCL1 | 4 |
| MF | GO:0002020 | protease binding | 3/17 | 138/18369 | 0.000261169 | 0.004306687 | 0.001846922 | CASP1/TIMP1/F3 | 3 |
| MF | GO:0038187 | pattern recognition receptor activity | 2/17 | 27/18369 | 0.000279137 | 0.004306687 | 0.001846922 | TLR7/CLEC7A | 2 |
| MF | GO:0004866 | endopeptidase inhibitor activity | 3/17 | 173/18369 | 0.000506588 | 0.006824508 | 0.00292669 | C3/LTF/TIMP1 | 3 |
| MF | GO:0030414 | peptidase inhibitor activity | 3/17 | 180/18369 | 0.000568709 | 0.006824508 | 0.00292669 | C3/LTF/TIMP1 | 3 |
| MF | GO:0061135 | endopeptidase regulator activity | 3/17 | 187/18369 | 0.000635522 | 0.006863642 | 0.002943472 | C3/LTF/TIMP1 | 3 |
| MF | GO:0048018 | receptor ligand activity | 4/17 | 497/18369 | 0.000952 | 0.008658738 | 0.003713299 | TIMP1/CCL5/CCL2/CXCL1 | 4 |
| MF | GO:0048020 | CCR chemokine receptor binding | 2/17 | 50/18369 | 0.000962082 | 0.008658738 | 0.003713299 | CCL5/CCL2 | 2 |
| MF | GO:0005126 | cytokine receptor binding | 3/17 | 270/18369 | 0.001833248 | 0.014473249 | 0.006206852 | CCL5/CCL2/CXCL1 | 3 |
| MF | GO:0003725 | double-stranded RNA binding | 2/17 | 70/18369 | 0.001876162 | 0.014473249 | 0.006206852 | TLR7/ACTN1 | 2 |
| MF | GO:0004857 | enzyme inhibitor activity | 3/17 | 359/18369 | 0.004106625 | 0.029567699 | 0.012680105 | C3/LTF/TIMP1 | 3 |
| MF | GO:0004175 | endopeptidase activity | 3/17 | 428/18369 | 0.006698229 | 0.043092949 | 0.018480407 | CASP1/LTF/F3 | 3 |
| MF | GO:0030295 | protein kinase activator activity | 2/17 | 135/18369 | 0.006783149 | 0.043092949 | 0.018480407 | LTF/CCL5 | 2 |
| MF | GO:0019209 | kinase activator activity | 2/17 | 141/18369 | 0.007377791 | 0.044266744 | 0.018983789 | LTF/CCL5 | 2 |
| MF | GO:0005178 | integrin binding | 2/17 | 151/18369 | 0.008419608 | 0.045156227 | 0.019365243 | ACTN1/ITGB2 | 2 |
| MF | GO:0003796 | lysozyme activity | 1/17 | 10/18369 | 0.009218524 | 0.045156227 | 0.019365243 | LYZ | 1 |
| MF | GO:0050786 | RAGE receptor binding | 1/17 | 10/18369 | 0.009218524 | 0.045156227 | 0.019365243 | S100A8 | 1 |
| MF | GO:0008083 | growth factor activity | 2/17 | 160/18369 | 0.009410689 | 0.045156227 | 0.019365243 | TIMP1/CXCL1 | 2 |
| MF | GO:0001851 | complement component C3b binding | 1/17 | 11/18369 | 0.010135965 | 0.045156227 | 0.019365243 | ITGB2 | 1 |
| MF | GO:0004252 | serine-type endopeptidase activity | 2/17 | 170/18369 | 0.010570263 | 0.045156227 | 0.019365243 | LTF/F3 | 2 |
| MF | GO:0017166 | vinculin binding | 1/17 | 12/18369 | 0.011052605 | 0.045156227 | 0.019365243 | ACTN1 | 1 |
| MF | GO:0035197 | siRNA binding | 1/17 | 12/18369 | 0.011052605 | 0.045156227 | 0.019365243 | TLR7 | 1 |
| MF | GO:0035325 | Toll-like receptor binding | 1/17 | 13/18369 | 0.011968447 | 0.045156227 | 0.019365243 | S100A8 | 1 |
| MF | GO:0019887 | protein kinase regulator activity | 2/17 | 184/18369 | 0.012294885 | 0.045156227 | 0.019365243 | LTF/CCL5 | 2 |
| MF | GO:0061783 | peptidoglycan muralytic activity | 1/17 | 14/18369 | 0.01288349 | 0.045156227 | 0.019365243 | LYZ | 1 |
| MF | GO:0089720 | caspase binding | 1/17 | 14/18369 | 0.01288349 | 0.045156227 | 0.019365243 | CASP1 | 1 |
| MF | GO:0008236 | serine-type peptidase activity | 2/17 | 190/18369 | 0.013069513 | 0.045156227 | 0.019365243 | LTF/F3 | 2 |
| MF | GO:0017171 | serine hydrolase activity | 2/17 | 194/18369 | 0.013597596 | 0.045156227 | 0.019365243 | LTF/F3 | 2 |
| MF | GO:0050700 | CARD domain binding | 1/17 | 15/18369 | 0.013797736 | 0.045156227 | 0.019365243 | CASP1 | 1 |
| MF | GO:0008191 | metalloendopeptidase inhibitor activity | 1/17 | 16/18369 | 0.014711185 | 0.046729646 | 0.020040004 | TIMP1 | 1 |
| MF | GO:0019207 | kinase regulator activity | 2/17 | 211/18369 | 0.01594437 | 0.049199771 | 0.021099317 | LTF/CCL5 | 2 |
| MF | GO:0045236 | CXCR chemokine receptor binding | 1/17 | 18/18369 | 0.016535694 | 0.049607082 | 0.021273992 | CXCL1 | 1 |
| MF | GO:0036041 | long-chain fatty acid binding | 1/17 | 19/18369 | 0.017446756 | 0.050925665 | 0.021839466 | S100A8 | 1 |
| MF | GO:0001846 | opsonin binding | 1/17 | 21/18369 | 0.019266496 | 0.051412279 | 0.022048151 | ITGB2 | 1 |
| MF | GO:0008656 | cysteine-type endopeptidase activator activity involved in apoptotic process | 1/17 | 21/18369 | 0.019266496 | 0.051412279 | 0.022048151 | CASP1 | 1 |
| MF | GO:0099186 | structural constituent of postsynapse | 1/17 | 21/18369 | 0.019266496 | 0.051412279 | 0.022048151 | ACTN1 | 1 |
| MF | GO:0016004 | phospholipase activator activity | 1/17 | 22/18369 | 0.020175177 | 0.051412279 | 0.022048151 | CCL5 | 1 |
| MF | GO:0019956 | chemokine binding | 1/17 | 22/18369 | 0.020175177 | 0.051412279 | 0.022048151 | CXCR4 | 1 |
| MF | GO:0004435 | phosphatidylinositol phospholipase C activity | 1/17 | 24/18369 | 0.021990161 | 0.051412279 | 0.022048151 | CCL5 | 1 |
| MF | GO:0016505 | peptidase activator activity involved in apoptotic process | 1/17 | 24/18369 | 0.021990161 | 0.051412279 | 0.022048151 | CASP1 | 1 |
| MF | GO:0001637 | G protein-coupled chemoattractant receptor activity | 1/17 | 25/18369 | 0.022896466 | 0.051412279 | 0.022048151 | CXCR4 | 1 |
| MF | GO:0004950 | chemokine receptor activity | 1/17 | 25/18369 | 0.022896466 | 0.051412279 | 0.022048151 | CXCR4 | 1 |
| MF | GO:0030247 | polysaccharide binding | 1/17 | 25/18369 | 0.022896466 | 0.051412279 | 0.022048151 | CLEC7A | 1 |
| MF | GO:0060229 | lipase activator activity | 1/17 | 25/18369 | 0.022896466 | 0.051412279 | 0.022048151 | CCL5 | 1 |
| MF | GO:0001848 | complement binding | 1/17 | 26/18369 | 0.023801981 | 0.051412279 | 0.022048151 | ITGB2 | 1 |
| MF | GO:0098918 | structural constituent of synapse | 1/17 | 26/18369 | 0.023801981 | 0.051412279 | 0.022048151 | ACTN1 | 1 |
| MF | GO:0004629 | phospholipase C activity | 1/17 | 27/18369 | 0.024706706 | 0.052320083 | 0.022437462 | CCL5 | 1 |
| MF | GO:0048019 | receptor antagonist activity | 1/17 | 31/18369 | 0.028317719 | 0.058813724 | 0.02522226 | CCL5 | 1 |
| MF | GO:0030296 | protein tyrosine kinase activator activity | 1/17 | 34/18369 | 0.031017717 | 0.063205915 | 0.02710585 | CCL5 | 1 |
| MF | GO:0001530 | lipopolysaccharide binding | 1/17 | 37/18369 | 0.033710653 | 0.067421307 | 0.028913621 | LTF | 1 |
| MF | GO:0042056 | chemoattractant activity | 1/17 | 39/18369 | 0.035502029 | 0.069713075 | 0.029896446 | CCL5 | 1 |
| MF | GO:0030547 | signaling receptor inhibitor activity | 1/17 | 42/18369 | 0.038183233 | 0.072347179 | 0.03102608 | CCL5 | 1 |
| MF | GO:0042287 | MHC protein binding | 1/17 | 42/18369 | 0.038183233 | 0.072347179 | 0.03102608 | CLEC7A | 1 |
| MF | GO:0043028 | cysteine-type endopeptidase regulator activity involved in apoptotic process | 1/17 | 43/18369 | 0.039075408 | 0.072761104 | 0.031203592 | CASP1 | 1 |
| MF | GO:0015026 | coreceptor activity | 1/17 | 48/18369 | 0.04352461 | 0.079672167 | 0.034167401 | CXCR4 | 1 |
| MF | GO:0005504 | fatty acid binding | 1/17 | 49/18369 | 0.04441212 | 0.079941817 | 0.03428304 | S100A8 | 1 |
| MF | GO:0016504 | peptidase activator activity | 1/17 | 50/18369 | 0.045298856 | 0.080201253 | 0.0343943 | CASP1 | 1 |
| MF | GO:0061980 | regulatory RNA binding | 1/17 | 51/18369 | 0.046184817 | 0.080450971 | 0.034501391 | TLR7 | 1 |
| MF | GO:0004869 | cysteine-type endopeptidase inhibitor activity | 1/17 | 54/18369 | 0.048838059 | 0.083722387 | 0.035904338 | LTF | 1 |
| MF | GO:0030374 | nuclear receptor coactivator activity | 1/17 | 55/18369 | 0.049720929 | 0.083904067 | 0.035982251 | ACTN1 | 1 |
