# Supplementary material for: NET-Related Gene as Potential Diagnostic Biomarkers for Diabetic Tubulointerstitial Injury
Source: J Diabetes Res. 2024 May 10;2024:4815488. doi: 10.1155/2024/4815488 (PMC11101254; doi:10.1155/2024/4815488)
Supplement: Supporting Information — Additional supporting information can be found online in the Supporting Information section. Supporting Information S1: R language for DEGs. Table S2: DEGs identified in the gene expression microarray study. Table S3: GO enrichment analysis of DEG. Table S4: KEGG enrichment analysis of DEG. Table S5: DEG related to NETs identified through machine learning. [file 4815488.f1.zip › Supplementary table S4.docx]

| Supplementary table S4: KEGG enrichment analysis of DEG | | | | | | | |
| --- | --- | --- | --- | --- | --- | --- | --- |
| ID | Description | GeneRatio | BgRatio | pvalue | p.adjust | qvalue | Count |
| hsa05134 | Legionellosis | 4/14 | 56/8622 | 1.52E-06 | 9.29E-05 | 4.17E-05 | 4 |
| hsa04613 | Neutrophil extracellular trap formation | 5/14 | 191/8622 | 8.62E-06 | 0.000237727 | 0.000106659 | 5 |
| hsa05323 | Rheumatoid arthritis | 4/14 | 93/8622 | 1.17E-05 | 0.000237727 | 0.000106659 | 4 |
| hsa04061 | Viral protein interaction with cytokine and cytokine receptor | 4/14 | 100/8622 | 1.56E-05 | 0.000237892 | 0.000106733 | 4 |
| hsa05164 | Influenza A | 4/14 | 171/8622 | 0.000128017 | 0.001561802 | 0.000700722 | 4 |
| hsa04621 | NOD-like receptor signaling pathway | 4/14 | 186/8622 | 0.000177202 | 0.001703661 | 0.000764369 | 4 |
| hsa04062 | Chemokine signaling pathway | 4/14 | 192/8622 | 0.000200271 | 0.001703661 | 0.000764369 | 4 |
| hsa05133 | Pertussis | 3/14 | 76/8622 | 0.000223431 | 0.001703661 | 0.000764369 | 3 |
| hsa05417 | Lipid and atherosclerosis | 4/14 | 215/8622 | 0.000309211 | 0.001965148 | 0.000881688 | 4 |
| hsa04610 | Complement and coagulation cascades | 3/14 | 86/8622 | 0.000322155 | 0.001965148 | 0.000881688 | 3 |
| hsa05171 | Coronavirus disease - COVID-19 | 4/14 | 232/8622 | 0.000413435 | 0.002128535 | 0.000954994 | 4 |
| hsa04657 | IL-17 signaling pathway | 3/14 | 94/8622 | 0.000418728 | 0.002128535 | 0.000954994 | 3 |
| hsa05131 | Shigellosis | 4/14 | 247/8622 | 0.000524567 | 0.002164501 | 0.000971131 | 4 |
| hsa05142 | Chagas disease | 3/14 | 102/8622 | 0.000532254 | 0.002164501 | 0.000971131 | 3 |
| hsa05146 | Amoebiasis | 3/14 | 102/8622 | 0.000532254 | 0.002164501 | 0.000971131 | 3 |
| hsa04668 | TNF signaling pathway | 3/14 | 114/8622 | 0.000736875 | 0.002712297 | 0.001216906 | 3 |
| hsa04670 | Leukocyte transendothelial migration | 3/14 | 115/8622 | 0.000755886 | 0.002712297 | 0.001216906 | 3 |
| hsa04060 | Cytokine-cytokine receptor interaction | 4/14 | 297/8622 | 0.001050406 | 0.003559711 | 0.001597109 | 4 |
| hsa04145 | Phagosome | 3/14 | 152/8622 | 0.001695291 | 0.005442776 | 0.00244197 | 3 |
| hsa05152 | Tuberculosis | 3/14 | 180/8622 | 0.002749046 | 0.00833317 | 0.003738782 | 3 |
| hsa05144 | Malaria | 2/14 | 50/8622 | 0.002868796 | 0.00833317 | 0.003738782 | 2 |
| hsa05163 | Human cytomegalovirus infection | 3/14 | 225/8622 | 0.005158916 | 0.014110704 | 0.006330946 | 3 |
| hsa04810 | Regulation of actin cytoskeleton | 3/14 | 229/8622 | 0.005419335 | 0.014110704 | 0.006330946 | 3 |
| hsa05120 | Epithelial cell signaling in Helicobacter pylori infection | 2/14 | 70/8622 | 0.005551753 | 0.014110704 | 0.006330946 | 2 |
| hsa04623 | Cytosolic DNA-sensing pathway | 2/14 | 75/8622 | 0.006349859 | 0.015493657 | 0.006951425 | 2 |
| hsa05140 | Leishmaniasis | 2/14 | 77/8622 | 0.00668299 | 0.015679322 | 0.007034726 | 2 |
| hsa05150 | Staphylococcus aureus infection | 2/14 | 96/8622 | 0.010233399 | 0.023119901 | 0.010373036 | 2 |
| hsa04933 | AGE-RAGE signaling pathway in diabetic complications | 2/14 | 100/8622 | 0.011067567 | 0.024111485 | 0.010817923 | 2 |
| hsa04625 | C-type lectin receptor signaling pathway | 2/14 | 104/8622 | 0.011931077 | 0.025096404 | 0.011259819 | 2 |
| hsa04620 | Toll-like receptor signaling pathway | 2/14 | 108/8622 | 0.012823571 | 0.026074595 | 0.011698697 | 2 |
| hsa05135 | Yersinia infection | 2/14 | 137/8622 | 0.020128543 | 0.038370035 | 0.017215201 | 2 |
| hsa05322 | Systemic lupus erythematosus | 2/14 | 137/8622 | 0.020128543 | 0.038370035 | 0.017215201 | 2 |
| hsa04936 | Alcoholic liver disease | 2/14 | 142/8622 | 0.021530454 | 0.039798718 | 0.017856198 | 2 |
| hsa05167 | Kaposi sarcoma-associated herpesvirus infection | 2/14 | 194/8622 | 0.038375414 | 0.068850008 | 0.030890426 | 2 |
| hsa05203 | Viral carcinogenesis | 2/14 | 204/8622 | 0.042054762 | 0.073295442 | 0.032884927 | 2 |
