# Supplementary material for: NET-Related Gene as Potential Diagnostic Biomarkers for Diabetic Tubulointerstitial Injury
Source: J Diabetes Res. 2024 May 10;2024:4815488. doi: 10.1155/2024/4815488 (PMC11101254; doi:10.1155/2024/4815488)
Supplement: Supporting Information — Additional supporting information can be found online in the Supporting Information section. Supporting Information S1: R language for DEGs. Table S2: DEGs identified in the gene expression microarray study. Table S3: GO enrichment analysis of DEG. Table S4: KEGG enrichment analysis of DEG. Table S5: DEG related to NETs identified through machine learning. [file 4815488.f1.zip › Supplementary table S5.docx]

| Supplementary table S5:DEG related to NETs identified through machine learning | | | | | |
| --- | --- | --- | --- | --- | --- |
| GEO-magenta | NETs-gene | intersect | Random Foreast | Lasso | intersect |
| A2M | ACTB | ACTN1 | LTF | CASP1 | CASP1 |
| ABHD10 | ACTG1 | C3 | FCGR2B | CCL2 | LYZ |
| ACKR1 | ACTN1 | CASP1 | LYZ | LYZ |  |
| ACSBG2 | ACTN4 | CCL2 | CASP1 |  |  |
| ACTA2 | AKT1 | CCL5 | CLEC7A |  |  |
| ACTN1 | AKT2 | CLEC7A | C3 |  |  |
| ADAMDEC1 | ARPIN | CXCL1 | MNDA |  |  |
| ADCY7 | ATG7 | CXCR4 | CXCL1 |  |  |
| ADRB2 | AZU1 | FCGR2B | ACTN1 |  |  |
| AEBP1 | C3 | IL33 | ITGB2 |  |  |
| AGPAT3 | C3AR1 | ITGB2 |  |  |  |
| AGR2 | C5AR1 | LTF |  |  |  |
| AGXT | CAMP | LYZ |  |  |  |
| AHNAK2 | CARD11 | MNDA |  |  |  |
| AKR1B1 | CASP1 | TLR7 |  |  |  |
| ALOX5 | CAT |  |  |  |  |
| AOC1 | CCDC25 |  |  |  |  |
| APOBEC3B | CCL2 |  |  |  |  |
| ARHGAP15 | CCL3 |  |  |  |  |
| ARHGDIB | CCL4 |  |  |  |  |
| ARL4C | CCL5 |  |  |  |  |
| ARPC1B | CD177 |  |  |  |  |
| ARPC3 | CD274 |  |  |  |  |
| ASB9 | CD44 |  |  |  |  |
| BASP1 | CEBPB |  |  |  |  |
| BHLHE41 | CFTR |  |  |  |  |
| BIRC3 | CLEC4E |  |  |  |  |
| BPI | CLEC6A |  |  |  |  |
| BST2 | CLEC7A |  |  |  |  |
| C11ORF71 | CSF3 |  |  |  |  |
| C1QA | CTSC |  |  |  |  |
| C1QB | CTSG |  |  |  |  |
| C1R | CXCL1 |  |  |  |  |
| C1RL | CXCL2 |  |  |  |  |
| C3 | CXCR4 |  |  |  |  |
| C7 | CYBB |  |  |  |  |
| CARD8 | DEFA3 |  |  |  |  |
| CASP1 | DNAJB1 |  |  |  |  |
| CASP3 | DNASE1 |  |  |  |  |
| CAVIN1 | ELANE |  |  |  |  |
| CCL19 | ENO1 |  |  |  |  |
| CCL2 | ENTPD4 |  |  |  |  |
| CCL5 | F2RL2 |  |  |  |  |
| CCND2 | F3 |  |  |  |  |
| CCNE1 | FCAR |  |  |  |  |
| CCR2 | FCGR2B |  |  |  |  |
| CD163 | FGL2 |  |  |  |  |
| CD1C | GPBAR1 |  |  |  |  |
| CD2 | GSDMD |  |  |  |  |
| CD38 | H2AX |  |  |  |  |
| CD3D | HIF1A |  |  |  |  |
| CD48 | HMGB1 |  |  |  |  |
| CD52 | IL12A |  |  |  |  |
| CD53 | IL17A |  |  |  |  |
| CD69 | IL1B |  |  |  |  |
| CD83 | IL1RL1 |  |  |  |  |
| CDC25B | IL33 |  |  |  |  |
| CDC42SE1 | IL36RN |  |  |  |  |
| CDH9 | IL5 |  |  |  |  |
| CEL | IL6 |  |  |  |  |
| CELF2 | IL8 |  |  |  |  |
| CEP170 | ILK |  |  |  |  |
| CES3 | IRAK4 |  |  |  |  |
| CGA | IRF1 |  |  |  |  |
| CGGBP1 | ITGAM |  |  |  |  |
| CHI3L2 | ITGB1 |  |  |  |  |
| CHODL | ITGB2 |  |  |  |  |
| CKLF | KCNN3 |  |  |  |  |
| CKS1B | KLF2 |  |  |  |  |
| CLC | KRT10 |  |  |  |  |
| CLCN5 | LCP1 |  |  |  |  |
| CLCN6 | LDLR |  |  |  |  |
| CLDN4 | LPAR3 |  |  |  |  |
| CLEC4A | LTF |  |  |  |  |
| CLEC7A | LYZ |  |  |  |  |
| CLU | MAPK1 |  |  |  |  |
| CNNM2 | MAPK14 |  |  |  |  |
| COL15A1 | MAPK3 |  |  |  |  |
| COL16A1 | MAPK7 |  |  |  |  |
| COL1A2 | MCOLN3 |  |  |  |  |
| COL3A1 | MFN1 |  |  |  |  |
| COL4A1 | MFN2 |  |  |  |  |
| COL4A2 | MIR146A |  |  |  |  |
| COL6A3 | MIR21 |  |  |  |  |
| COPG2IT1 | MIR223 |  |  |  |  |
| CORO1A | MMP9 |  |  |  |  |
| CPA3 | MNDA |  |  |  |  |
| CPNE1 | MPO |  |  |  |  |
| CRIP1 | MTOR |  |  |  |  |
| CRISPLD2 | MYD88 |  |  |  |  |
| CRLF3 | MYH9 |  |  |  |  |
| CSF1R | NFE2L2 |  |  |  |  |
| CST6 | NFIL3 |  |  |  |  |
| CSTA | NFKBIA |  |  |  |  |
| CSTB | NLRP3 |  |  |  |  |
| CTSK | NOX4 |  |  |  |  |
| CTSS | OPA1 |  |  |  |  |
| CX3CR1 | ORAI1 |  |  |  |  |
| CXCL1 | P2RX1 |  |  |  |  |
| CXCL13 | PADI4 |  |  |  |  |
| CXCL6 | PARVB |  |  |  |  |
| CXCL8 | PF4 |  |  |  |  |
| CXCL9 | PIK3CA |  |  |  |  |
| CXCR4 | PKM |  |  |  |  |
| CYFIP2 | PROCR |  |  |  |  |
| CYP27B1 | PRTN3 |  |  |  |  |
| CYP3A7 | PTAFR |  |  |  |  |
| CYP3A7-CYP3A51P | RIPK1 |  |  |  |  |
| CYTIP | RIPK3 |  |  |  |  |
| DDX25 | S100A12 |  |  |  |  |
| DEFB1 | S100A8 |  |  |  |  |
| DENND5A | S100A9 |  |  |  |  |
| DEPP1 | S1PR2 |  |  |  |  |
| DHRS9 | SELP |  |  |  |  |
| DIP2C | SELPLG |  |  |  |  |
| DKK3 | SGK1 |  |  |  |  |
| DNMT3L | SGK1 |  |  |  |  |
| DOCK2 | SIGLEC14 |  |  |  |  |
| EAF2 | SOCS3 |  |  |  |  |
| ECT2 | SPP1 |  |  |  |  |
| EFHC1 | SRC |  |  |  |  |
| EFHD1 | STAT3 |  |  |  |  |
| EFNA4 | SUCNR1 |  |  |  |  |
| EGF | SYK |  |  |  |  |
| ELOVL6 | TICAM1 |  |  |  |  |
| EMP3 | TIMP1 |  |  |  |  |
| ENAH | TKT |  |  |  |  |
| ENC1 | TLR2 |  |  |  |  |
| ERAP2 | TLR4 |  |  |  |  |
| ESPL1 | TLR7 |  |  |  |  |
| ESYT1 | TLR8 |  |  |  |  |
| ETNPPL | TLR9 |  |  |  |  |
| EVI2A | TNF |  |  |  |  |
| EVI2B | TNFAIP3 |  |  |  |  |
| EYA2 | WASL |  |  |  |  |
| FABP5 | XIST |  |  |  |  |
| FAM110B | HRG |  |  |  |  |
| FBN1 |  |  |  |  |  |
| FCER1A |  |  |  |  |  |
| FCGR1B |  |  |  |  |  |
| FCGR2A |  |  |  |  |  |
| FCGR2B |  |  |  |  |  |
| FCGR3B |  |  |  |  |  |
| FCN1 |  |  |  |  |  |
| FHL2 |  |  |  |  |  |
| FKBP11 |  |  |  |  |  |
| FMO5 |  |  |  |  |  |
| FN1 |  |  |  |  |  |
| FOLR1 |  |  |  |  |  |
| FOLR2 |  |  |  |  |  |
| FOXO1 |  |  |  |  |  |
| FRZB |  |  |  |  |  |
| FXYD6 |  |  |  |  |  |
| FZD2 |  |  |  |  |  |
| FZD7 |  |  |  |  |  |
| G6PC |  |  |  |  |  |
| GABRP |  |  |  |  |  |
| GBP2 |  |  |  |  |  |
| GHR |  |  |  |  |  |
| GLIPR1 |  |  |  |  |  |
| GPR171 |  |  |  |  |  |
| GPR18 |  |  |  |  |  |
| GPR183 |  |  |  |  |  |
| GPX7 |  |  |  |  |  |
| GRAMD1B |  |  |  |  |  |
| GSTA3 |  |  |  |  |  |
| GZMA |  |  |  |  |  |
| GZMB |  |  |  |  |  |
| GZMK |  |  |  |  |  |
| H1FX |  |  |  |  |  |
| H3F3B |  |  |  |  |  |
| HAAO |  |  |  |  |  |
| HCK |  |  |  |  |  |
| HCLS1 |  |  |  |  |  |
| HDAC9 |  |  |  |  |  |
| HECTD3 |  |  |  |  |  |
| HIKESHI |  |  |  |  |  |
| HLA-DMA |  |  |  |  |  |
| HLA-DMB |  |  |  |  |  |
| HLA-DPA1 |  |  |  |  |  |
| HLA-DPB1 |  |  |  |  |  |
| HLA-DQA1 |  |  |  |  |  |
| HLA-DRB1 |  |  |  |  |  |
| HLA-F |  |  |  |  |  |
| HLA-J |  |  |  |  |  |
| HOPX |  |  |  |  |  |
| HOXA10 |  |  |  |  |  |
| HPGD |  |  |  |  |  |
| HPGDS |  |  |  |  |  |
| HRG |  |  |  |  |  |
| HSD11B2 |  |  |  |  |  |
| HTR2B |  |  |  |  |  |
| HYAL1 |  |  |  |  |  |
| ICAM2 |  |  |  |  |  |
| IFI27 |  |  |  |  |  |
| IFI44L |  |  |  |  |  |
| IFI6 |  |  |  |  |  |
| IFITM1 |  |  |  |  |  |
| IFITM2 |  |  |  |  |  |
| IFITM3 |  |  |  |  |  |
| IGFBP6 |  |  |  |  |  |
| IGHM |  |  |  |  |  |
| IGKC |  |  |  |  |  |
| IGLC1 |  |  |  |  |  |
| IGLL3P |  |  |  |  |  |
| IGLL5 |  |  |  |  |  |
| IGLV1-44 |  |  |  |  |  |
| IGSF6 |  |  |  |  |  |
| IL10RA |  |  |  |  |  |
| IL33 |  |  |  |  |  |
| IL7R |  |  |  |  |  |
| IP6K2 |  |  |  |  |  |
| IRF8 |  |  |  |  |  |
| IRF9 |  |  |  |  |  |
| ISG15 |  |  |  |  |  |
| ISG20 |  |  |  |  |  |
| ITGB2 |  |  |  |  |  |
| ITGBL1 |  |  |  |  |  |
| ITM2A |  |  |  |  |  |
| ITM2C |  |  |  |  |  |
| JCHAIN |  |  |  |  |  |
| JPT1 |  |  |  |  |  |
| KCNJ8 |  |  |  |  |  |
| KLF9 |  |  |  |  |  |
| KLK1 |  |  |  |  |  |
| KLKB1 |  |  |  |  |  |
| KLRB1 |  |  |  |  |  |
| KNG1 |  |  |  |  |  |
| LAMP3 |  |  |  |  |  |
| LAPTM5 |  |  |  |  |  |
| LCN2 |  |  |  |  |  |
| LCP2 |  |  |  |  |  |
| LGALS1 |  |  |  |  |  |
| LGSN |  |  |  |  |  |
| LHX6 |  |  |  |  |  |
| LOC101929272 |  |  |  |  |  |
| LPAR1 |  |  |  |  |  |
| LPCAT1 |  |  |  |  |  |
| LPL |  |  |  |  |  |
| LTB |  |  |  |  |  |
| LTF |  |  |  |  |  |
| LUM |  |  |  |  |  |
| LY75 |  |  |  |  |  |
| LY86 |  |  |  |  |  |
| LY96 |  |  |  |  |  |
| LYN |  |  |  |  |  |
| LYPD1 |  |  |  |  |  |
| LYZ |  |  |  |  |  |
| MAGED4B |  |  |  |  |  |
| MARCKS |  |  |  |  |  |
| MARCKSL1 |  |  |  |  |  |
| MCAM |  |  |  |  |  |
| MCUR1 |  |  |  |  |  |
| MELK |  |  |  |  |  |
| MGP |  |  |  |  |  |
| MID1 |  |  |  |  |  |
| MMP7 |  |  |  |  |  |
| MNDA |  |  |  |  |  |
| MNS1 |  |  |  |  |  |
| MOXD1 |  |  |  |  |  |
| MPC1 |  |  |  |  |  |
| MRC1 |  |  |  |  |  |
| MS4A4A |  |  |  |  |  |
| MS4A6A |  |  |  |  |  |
| MST1 |  |  |  |  |  |
| MTTP |  |  |  |  |  |
| MX1 |  |  |  |  |  |
| MYC |  |  |  |  |  |
| MYL12B |  |  |  |  |  |
| MYOM2 |  |  |  |  |  |
| MZB1 |  |  |  |  |  |
| NCF1 |  |  |  |  |  |
| NCF2 |  |  |  |  |  |
| NELL1 |  |  |  |  |  |
| NFKBIE |  |  |  |  |  |
| NIBAN1 |  |  |  |  |  |
| NINJ2 |  |  |  |  |  |
| NLGN4X |  |  |  |  |  |
| NMI |  |  |  |  |  |
| NNMT |  |  |  |  |  |
| NPDC1 |  |  |  |  |  |
| NPR3 |  |  |  |  |  |
| NREP |  |  |  |  |  |
| NT5DC2 |  |  |  |  |  |
| NUAK1 |  |  |  |  |  |
| NUP85 |  |  |  |  |  |
| OPCML |  |  |  |  |  |
| P2RY13 |  |  |  |  |  |
| P2RY14 |  |  |  |  |  |
| PDLIM1 |  |  |  |  |  |
| PDZD2 |  |  |  |  |  |
| PER1 |  |  |  |  |  |
| PFN1 |  |  |  |  |  |
| PLA2G4A |  |  |  |  |  |
| PLAAT4 |  |  |  |  |  |
| PLAC8 |  |  |  |  |  |
| PLCB4 |  |  |  |  |  |
| PLK2 |  |  |  |  |  |
| PLPP2 |  |  |  |  |  |
| PLSCR1 |  |  |  |  |  |
| PLXND1 |  |  |  |  |  |
| POGLUT2 |  |  |  |  |  |
| POU2AF1 |  |  |  |  |  |
| PPCDC |  |  |  |  |  |
| PPP1R16B |  |  |  |  |  |
| PPP1R3C |  |  |  |  |  |
| PPP2R1B |  |  |  |  |  |
| PPP2R2A |  |  |  |  |  |
| PRC1 |  |  |  |  |  |
| PRF1 |  |  |  |  |  |
| PRKCB |  |  |  |  |  |
| PRKX |  |  |  |  |  |
| PROZ |  |  |  |  |  |
| PRRC1 |  |  |  |  |  |
| PSMB10 |  |  |  |  |  |
| PSMB8 |  |  |  |  |  |
| PSTPIP2 |  |  |  |  |  |
| PTGER3 |  |  |  |  |  |
| PTMA |  |  |  |  |  |
| PTN |  |  |  |  |  |
| PTPRC |  |  |  |  |  |
| PTPRE |  |  |  |  |  |
| PXDN |  |  |  |  |  |
| PXMP2 |  |  |  |  |  |
| PYCARD |  |  |  |  |  |
| QPCT |  |  |  |  |  |
| QSOX1 |  |  |  |  |  |
| RAI2 |  |  |  |  |  |
| RARRES1 |  |  |  |  |  |
| RASSF2 |  |  |  |  |  |
| RBM10 |  |  |  |  |  |
| RBP4 |  |  |  |  |  |
| RCN1 |  |  |  |  |  |
| REG1A |  |  |  |  |  |
| RGS19 |  |  |  |  |  |
| RNASE1 |  |  |  |  |  |
| RNASE6 |  |  |  |  |  |
| RPA3 |  |  |  |  |  |
| RRAS |  |  |  |  |  |
| S100A4 |  |  |  |  |  |
| SALL1 |  |  |  |  |  |
| SAMHD1 |  |  |  |  |  |
| SAMSN1 |  |  |  |  |  |
| SCAMP2 |  |  |  |  |  |
| SCRN1 |  |  |  |  |  |
| SELL |  |  |  |  |  |
| SERPINA3 |  |  |  |  |  |
| SERPINA6 |  |  |  |  |  |
| SERPINB9 |  |  |  |  |  |
| SERPING1 |  |  |  |  |  |
| SLA |  |  |  |  |  |
| SLAMF8 |  |  |  |  |  |
| SLC12A2 |  |  |  |  |  |
| SLC12A6 |  |  |  |  |  |
| SLC16A7 |  |  |  |  |  |
| SLC25A44 |  |  |  |  |  |
| SLC2A5 |  |  |  |  |  |
| SLC46A3 |  |  |  |  |  |
| SLC5A3 |  |  |  |  |  |
| SLCO3A1 |  |  |  |  |  |
| SLIT2 |  |  |  |  |  |
| SLPI |  |  |  |  |  |
| SMARCD3 |  |  |  |  |  |
| SNRPA |  |  |  |  |  |
| SOD2 |  |  |  |  |  |
| SOX4 |  |  |  |  |  |
| SPAG5 |  |  |  |  |  |
| SPON2 |  |  |  |  |  |
| SRGN |  |  |  |  |  |
| SRPX |  |  |  |  |  |
| ST5 |  |  |  |  |  |
| STAB1 |  |  |  |  |  |
| STBD1 |  |  |  |  |  |
| STK10 |  |  |  |  |  |
| SUMO4 |  |  |  |  |  |
| SYNC |  |  |  |  |  |
| SYT11 |  |  |  |  |  |
| TAC1 |  |  |  |  |  |
| TAGLN |  |  |  |  |  |
| TAX1BP3 |  |  |  |  |  |
| TBC1D4 |  |  |  |  |  |
| TCF4 |  |  |  |  |  |
| TDO2 |  |  |  |  |  |
| TENM1 |  |  |  |  |  |
| TES |  |  |  |  |  |
| TGFBI |  |  |  |  |  |
| THBS2 |  |  |  |  |  |
| THY1 |  |  |  |  |  |
| TINF2 |  |  |  |  |  |
| TLR1 |  |  |  |  |  |
| TLR7 |  |  |  |  |  |
| TM7SF3 |  |  |  |  |  |
| TMED3 |  |  |  |  |  |
| TMEM204 |  |  |  |  |  |
| TMLHE |  |  |  |  |  |
| TMSB10 |  |  |  |  |  |
| TMSB4X |  |  |  |  |  |
| TNC |  |  |  |  |  |
| TNFAIP6 |  |  |  |  |  |
| TNFAIP8 |  |  |  |  |  |
| TNFRSF17 |  |  |  |  |  |
| TNFRSF1B |  |  |  |  |  |
| TPM1 |  |  |  |  |  |
| TRBC1 |  |  |  |  |  |
| TRIM5 |  |  |  |  |  |
| TRIOBP |  |  |  |  |  |
| TSPAN13 |  |  |  |  |  |
| TUBA1A |  |  |  |  |  |
| TUBB |  |  |  |  |  |
| TXNIP |  |  |  |  |  |
| TYROBP |  |  |  |  |  |
| TYRP1 |  |  |  |  |  |
| UBD |  |  |  |  |  |
| UBE2L6 |  |  |  |  |  |
| UMOD |  |  |  |  |  |
| USP11 |  |  |  |  |  |
| USP2 |  |  |  |  |  |
| VCAN |  |  |  |  |  |
| VOPP1 |  |  |  |  |  |
